# Comparison Theorems for Stochastic Chemical Reaction Networks

**DOI:** 10.1007/s11538-023-01136-5

**Published:** 2023-03-31

**Authors:** Felipe A. Campos, Simone Bruno, Yi Fu, Domitilla Del Vecchio, Ruth J. Williams

**Affiliations:** 1 Department of Mathematics, University of California, San Diego, 9500 Gilman Drive, La Jolla, CA 92093-0112 USA; 2 Department of Mechanical Engineering, Massachusetts Institute of Technology, 77 Massachusetts Avenue, Cambridge, MA 02139 USA

**Keywords:** Stochastic chemical reaction networks, Monotonicity

## Abstract

Continuous-time Markov chains are frequently used as stochastic models for chemical reaction networks, especially in the growing field of systems biology. A fundamental problem for these Stochastic Chemical Reaction Networks (SCRNs) is to understand the dependence of the stochastic behavior of these systems on the chemical reaction rate parameters. Towards solving this problem, in this paper we develop theoretical tools called comparison theorems that provide stochastic ordering results for SCRNs. These theorems give sufficient conditions for monotonic dependence on parameters in these network models, which allow us to obtain, under suitable conditions, information about transient and steady-state behavior. These theorems exploit structural properties of SCRNs, beyond those of general continuous-time Markov chains. Furthermore, we derive two theorems to compare stationary distributions and mean first passage times for SCRNs with different parameter values, or with the same parameters and different initial conditions. These tools are developed for SCRNs taking values in a generic (finite or countably infinite) state space and can also be applied for non-mass-action kinetics models. When propensity functions are bounded, our method of proof gives an explicit method for coupling two comparable SCRNs, which can be used to simultaneously simulate their sample paths in a comparable manner. We illustrate our results with applications to models of enzymatic kinetics and epigenetic regulation by chromatin modifications.

**Supplementary Information:**

The online version contains supplementary material available at 10.1007/s11538-023-01136-5.

## Introduction

### Overview

Stochastic Chemical Reaction Networks (SCRNs) are a class of continuous-time Markov chain models used to describe the stochastic dynamics of a chemical system undergoing a series of reactions which change the numbers of molecules of a finite set of species over time. These models provide a framework for the theoretical study of biochemical systems in areas such as intracellular viral kinetics (Srivastava et al. (2002) and Haseltine and Rawlings (2002)), enzymatic kinetics (see Kang et al. (2019) for example) and epigenetic regulation by chromatin modifications (see Bruno et al. (2022) for a recently developed model of chromatin regulation).

One of the most interesting questions for biochemical system models is: “What effect does changing reaction rate parameters have on system dynamics?" Indeed, different rate parameters for chemical processes can lead to different stochastic behaviors. One possible approach to evaluate the effect of parameter variations on system dynamics is through comparison theorems for stochastic processes. More precisely, this type of theorem provides inequalities between stochastic processes (see Muller and Stoyan (2002) for a general reference on this topic).

In this paper, we employ uniformization and coupling methods (see Grassmann (1977) and Keilson (1979)) to derive comparison theorems for SCRNs under verifiable sufficient conditions. These theoretical results enable us to develop two novel theorems yielding a direct comparison of mean first passage times and stationary distributions between SCRNs with different rate parameters or initial conditions. We apply these theorems to several examples to illustrate how they can be used to understand how key biological parameters affect stochastic behavior. While a major motivator for our work has been the study of SCRNs, we state our theorems in the context of continuous-time Markov chains, for which the state space is a subset of $\mathbb {Z}_+^d$ (the set of *d*-dimensional vectors with nonnegative integer entries), and the set of all possible transition vectors is a finite set. This thereby allows for other applications that have similar characteristics to SCRNs. In addition, for the case of bounded transition intensities satisfying our conditions, we give an explicit concrete coupling of two comparable Markov chains, which can be used to simultaneously simulate them in such a way that their sample paths are monotonically related.

The paper is structured as follows: in Sect. 2 we introduce some background on stochastic chemical reaction networks needed for this article. We present the main results in Sect. 3, with proofs provided in Sect. 5. In Sect. 4 we apply our theoretical tools to several examples, such as epigenetic regulation by chromatin modifications and enzymatic kinetics. Concluding remarks are presented in Sect. 6. The Supplementary information (SI) file contains some further details and extensions of the main results and examples in the paper.

### Related Work

Due to the growing field of systems biology, the mathematical study of chemical reaction networks has seen a wealth of activity lately. Concerning comparison results, considerable work has been conducted on monotonicity properties for deterministic models of chemical reaction networks, i.e., systems of ordinary differential equations describing the dynamics of species concentrations. For example, Angeli et al. (2006) proposed a graphical method, based on the monotonicity properties of the reaction rates with respect to species concentrations, to determine global stability properties for the models. More recently, Gori et al. (2019) introduced sufficient conditions to verify the existence of a monotonicity property for the concentrations of species for any positive time with respect to their initial concentrations. However, these works do not address how changing parameters affects the behavior of stochastic models.

To the best of our knowledge, no systematic study of stochastic ordering has been conducted for stochastic chemical reaction networks. On a more general level, theorems have been established for stochastic processes and have been specialized for particular classes such as for queueing systems and point processes (see Muller and Stoyan (2002) for an introduction to the topic). For Markov chains, the work of Massey (1987) is of special interest, since he establishes criteria for comparison of continuous-time Markov chains in terms of their infinitesimal generators. For relevant work prior to Massey, there is a nice summary in Massey (1987). In particular, Kamae et al. (1977) showed that for Markov processes, a comparison between transition probability functions, at all fixed times and for all partially ordered starting points, can be realized in a pathwise stochastic comparison between versions of the Markov processes. In relation to Massey’s work, our results provide simplified conditions and extended results for stochastic comparisons, which exploit the structure of stochastic chemical reaction networks. Furthermore, unlike Massey, we do not require a uniform bound on the rates of leaving each state. In addition, under the latter assumption, we explicitly construct versions of the stochastic processes on the same probability space that have comparable sample paths. More detail on the relationship of our work to that of Massey is given in Remark 3.2. In contrast to work on sensitivity analysis of distributions at a finite set of times and which considers only local changes in parameters (see for example Gunawan et al. (2005), Gupta and Khammash (2014) and references therein), our work provides a sample path comparison between stochastic processes for global changes in their parameters.

### Notation and Terminology

Denote by $\mathbb {Z}_+ = \{0,1,2, \ldots \}$ the set of nonnegative integers. For an integer $d \ge 1$ we denote by $\mathbb {Z}_+^d$ the set of *d*-dimensional vectors with entries in $\mathbb {Z}_+$. For any integer $d \ge 1$, let $\mathbb {R}^d$ denote the *d*-dimensional Euclidean space. We usually write $\mathbb {R}$ for $\mathbb {R}^1$. We denote by $\mathbb {R}^d_+$ the set of vectors $x \in \mathbb {R}^d$ such that $x_i \ge 0$ for every $1 \le i \le d$. For $x \in \mathbb {R}^d$, let $\Vert x\Vert _{\infty } = \sup _{1 \le i \le d} |x_i|$ be the supremum norm. In this paper, the sum over the empty set is considered to be 0.

A binary relation $\preccurlyeq$ on a set ${\mathcal {X}}$ will be called reflexive if $x \preccurlyeq x$ for every $x \in {\mathcal {X}}$, transitive if $x \preccurlyeq y$ and $y \preccurlyeq z$ implies $x \preccurlyeq z$ for every $x,y,z \in {\mathcal {X}}$ and antisymmetric if $x \preccurlyeq y$ and $y \preccurlyeq x$ implies $x=y$ for every $x,y \in {\mathcal {X}}$. A preorder is a binary relation that is reflexive and transitive. A partial order is a preorder that is antisymmetric.

In this paper, a probability space $(\Omega ,{\mathcal {F}},\mathbb {P})$ will consist of a sample space $\Omega$, a $\sigma$-algebra of events ${\mathcal {F}}$ and a probability measure $\mathbb {P}$ on $(\Omega , {\mathcal {F}})$. We will say that two real-valued random variables $Y,Y'$ (defined on possibly different probability spaces) are equal in distribution, denoted as $Y' \overset{\text {dist}}{=} Y$, if their cumulative distribution functions agree. All stochastic processes considered in this paper will have right-continuous sample paths that also have finite left-limits.

## Stochastic Chemical Reaction Networks (SCRNs)

In this section, we provide necessary background on Stochastic Chemical Reaction Networks. The reader is referred to Anderson and Kurtz (2015) for an introduction to this subject.

We assume there is a finite non-empty set ${\mathscr {S}}= \{\textrm{S}_1,\ldots ,\textrm{S}_d\}$ of *d* **species**, and a finite non-empty set ${\mathscr {R}}\subseteq \mathbb {Z}_+^d \times \mathbb {Z}_+^d$ that represents chemical **reactions**. We assume that $(w,w) \notin {\mathscr {R}}$ for every $w \in \mathbb {Z}^d_+$. The set ${\mathscr {S}}$ represents *d* different molecular species in a system subject to reactions ${\mathscr {R}}$ which change the number of molecules of each species. For each $(v^{-},v^+) \in {\mathscr {R}}$, the *d*-dimensional vector $v^{-}$ (the **reactant vector**) counts how many molecules of each species are consumed in the reaction, while $v^{+}$ (the **product vector**) counts how many molecules of each species are produced. The reaction is usually written as

1 $$\begin{aligned} \sum _{i=1}^d (v^{-})_{i}\textrm{S}_i \longrightarrow \sum _{i=1}^d (v^{+})_{i}\textrm{S}_i. \end{aligned}$$

To avoid the use of unnecessary symbols, we will assume that for each $1 \le i \le d$, there exists a vector $w=(a_1, \ldots , a_d)^T \in \mathbb {Z}_+^d$ with $a_i >0$ such that (*w*, *v*) or (*v*, *w*) is in ${\mathscr {R}}$ for some $v \in \mathbb {Z}^d_+$, i.e., each species is either a reactant or a product in some reaction.

The net change in the quantity of molecules of each species due to a reaction $(v^{-},v^{+}) \in {\mathscr {R}}$ is described by $v^{+}-v^{-}$ and it is called the associated **reaction vector**. We denote the set of reaction vectors ${\mathcal {V}}:= \{ v \in \mathbb {Z}^d \,|\, v = v^{+}- v^{-} \text { for some } (v^{-},v^{+}) \in {\mathscr {R}}\}$, let $n:= |{\mathcal {V}}|$ the size of ${\mathcal {V}}$ and enumerate the members of ${\mathcal {V}}$ as $\{v_1,\ldots ,v_n\}$. Note that ${\mathcal {V}}$ does not contain the zero vector because ${\mathscr {R}}$ has no elements of the form (*w*, *w*). Different reactions might have the same reaction vector. For each $v_j \in {\mathcal {V}}$ we consider the set ${\mathscr {R}}_{v_j}:= \{(v^{-},v^{+}) \in {\mathscr {R}}\,|\, v_j =v^{+}-v^{-} \}$. The reaction vectors generate the **stoichiometric subspace** ${\mathcal {L}}:= {{\,\textrm{span}\,}}({\mathcal {V}})$. For $z \in \mathbb {R}^d$, we call $z + {\mathcal {L}}$ a **stoichiometric compatibility class**.

Given $({\mathscr {S}},{\mathscr {R}})$ we will consider an associated continuous-time Markov chain $X=(X_1,\ldots ,X_d)$, with a state space ${\mathcal {X}}$ contained in $\mathbb {Z}^d_+$, which tracks the number of molecules of each species over time. Roughly speaking, the dynamics of *X* will be given by the following: given a current state $x=(x_1,\ldots ,x_d) \in {\mathcal {X}}\subseteq \mathbb {Z}_+^{d}$, for each reaction $(v^{-},v^{+}) \in {\mathscr {R}}$, there is a clock which will ring at an exponentially distributed time (with rate $\Lambda _{(v^{-},v^{+})}(x)$). The clocks for distinct reactions are independent of one another. If the clock corresponding to $(v^{-},v^{+})\in {\mathscr {R}}$ rings first, the system moves from *x* to $x+v^{+}- v^{-}$ at that time, and then the process repeats. We now define the Markov chain in more detail.

Consider a set of species ${\mathscr {S}}$ and of reactions ${\mathscr {R}}$, a set ${\mathcal {X}}\subseteq \mathbb {Z}^d_+$ and a collection of functions $\{\Lambda _{(v^{-},v^{+})}:{\mathcal {X}}\longrightarrow \mathbb {R}_+\}_{(v^{-},v^{+}) \in {\mathscr {R}}}$ such that for each $x \in {\mathcal {X}}$ and $(v^{-},v^{+}) \in {\mathscr {R}}$, if $x+v^{+}-v^{-} \notin {\mathcal {X}}$, then $\Lambda _{(v^{-},v^{+})}(x)=0$. Now, for $1 \le j \le n$, $v_j \in {\mathcal {V}}$, define

2 $$\begin{aligned} \Upsilon _j(x):= \sum _{(v^{-},v^{+}) \in {\mathscr {R}}_{v_j}} \Lambda _{(v^{-},v^{+})}(x). \end{aligned}$$

Note that for each $x \in {\mathcal {X}}$ and $1 \le j \le n$, if $x +v_j \notin {\mathcal {X}}$, then $\Upsilon _j(x) = 0$. A **stochastic chemical reaction network (SCRN)** is a Markov chain *X* with state space ${\mathcal {X}}$ and infinitesimal generator^1^*Q* given for $x,y \in {\mathcal {X}}$ by

3 $$\begin{aligned} Q_{x,y} = {\left\{ \begin{array}{ll} \Upsilon _j(x) &{} \text { if } y-x = v_j \text { for some } 1 \le j \le n, \\ - \sum _{j=1}^n\Upsilon _j(x) &{} \text { if } y = x, \\ 0 &{} \text { otherwise.} \end{array}\right. } \end{aligned}$$

The functions $\{\Lambda _{(v^{-},v^{+})}:{\mathcal {X}}\longrightarrow \mathbb {R}_+\}_{(v^{-},v^{+}) \in {\mathscr {R}}}$ are called **propensity** or **intensity** functions. A common form for the propensity functions is the following associated with **mass action kinetics**:

4 $$\begin{aligned} \Lambda _{(v^{-},v^{+})}(x) = \kappa _{(v^{-},v^{+})}\prod _{i=1}^{d}(x_i)_{(v^{-})_i}, \end{aligned}$$

where $\{\kappa _{(v^{-},v^{+})}\}_{(v^{-},v^{+}) \in {\mathscr {R}}}$ are positive constants and for $m,\ell \in \mathbb {Z}_+$, the quantity $(m)_\ell$ is the falling factorial, i.e., $(m)_0:= 1$ and $(m)_\ell := m(m-1)\ldots (m-\ell +1)$.

### Remark 2.1

Our definition of SCRN allows for some model flexibility. Notice that the propensity functions are not necessarily defined on the whole lattice $\mathbb {Z}^d_+$ and they are not necessarily of the form (4). Indeed, in some of our applications, mass-conservation laws restrict the possible values that *X* may take (see Example 4.4). In addition, there may be other types of kinetics, such as those described by Hill functions (see Example 4.5).

A convenient way to represent such a Markov chain is given in Theorem 6.4.1 of Ethier and Kurtz (1986). For this, consider a probability space $(\Omega , {\mathcal {F}}, \mathbb {P})$ equipped with independent unit rate Poisson processes $N_1, \ldots ,N_n$. There is a version of *X* defined on $(\Omega ,{\mathcal {F}},\mathbb {P})$ such that

5 $$\begin{aligned} X(t) = X(0) + \sum _{j=1}^{n} v_jN_j\left( \int _{0}^{t}\Upsilon _j\left( X(s)\right) ds \right) , \end{aligned}$$

for every $0 \le t < \tau$, where $\tau$ is the explosion time for *X* (which may be $+\infty$). From (5), it is easy to see that for a SCRN *X* with initial state $z \in {\mathcal {X}}$, *X*(*t*) will stay in the stoichiometric compatibility class $z +{\mathcal {L}}$ intersected with $\mathbb {Z}^d_+$ for all time $0 \le t < \tau$, with probability one. For this reason, sometimes it will be convenient to choose ${\mathcal {X}}= (z+{\mathcal {L}}) \cap \mathbb {Z}^d_+$, for a fixed $z \in \mathbb {Z}^d_+$.

While our work was initially motivated by questions for SCRNs, we will first develop our results in a more general context of continuous-time Markov chains, for which the state space is contained in $\mathbb {Z}^d_+$ and the set of all possible transition vectors is a finite set, and then illustrate them for SCRNs.

## Main Results

The general stochastic ordering results provided in this paper are relative to a preorder relation on a state space ${\mathcal {X}}\subseteq \mathbb {Z}^d_+ \subseteq \mathbb {R}^d$. We will define the preorder on all of $\mathbb {R}^d$ and then restrict it to various subsets. We introduce this notation and related notation in Sect. 3.1. In Sect. 3.2 we present the main results of this article, and in Sect. 3.3 we discuss relevant consequences for the comparison of (mean) first passage times and stationary distributions.

### Preorders in $\mathbb {R}^d$

Let $m,d \ge 1$ be integers. Denote by $\le$ the usual componentwise partial order on $\mathbb {R}^d$, i.e., for $x,y \in \mathbb {R}^d$, $x \le y$ whenever $x_i \le y_i$ for every $1 \le i \le d$. Additionally, we write $x < y$ whenever $x_i < y_i$ for every $1 \le i \le d$. For the rest of the paper, we consider a matrix $A \in \mathbb {R}^{m \times d}$, where no row of *A* is identically zero.

#### Definition 3.1

For $x,y \in \mathbb {R}^d$, we say that $x \preccurlyeq _A y$ whenever $A(y-x) \ge 0$.

For the matrix *A*, consider the convex cone $K_A:= \{ x \in \mathbb {R}^d \,|\, Ax \ge 0\}$. Note that $x \preccurlyeq _A y$ holds if and only if $y-x \in K_A$. Moreover, the relation $\preccurlyeq _A$ is reflexive and transitive, and therefore a preorder on $\mathbb {R}^d$. Also, for this relation,

6 $$\begin{aligned} \text {if } x \preccurlyeq _A y, \text { then } x +z \preccurlyeq _A y +z \text { for any } z \in \mathbb {R}^d. \end{aligned}$$

For any $x \in \mathbb {R}^d$ consider the set

$$\begin{aligned} K_A +x =\{ y \in \mathbb {R}^d \,|\, A(y-x) \ge 0 \} = \{ y \in \mathbb {R}^d \,|\, x \preccurlyeq _A y \}. \end{aligned}$$

In the coming sections, we will consider the notions of increasing and decreasing sets with respect to $\preccurlyeq _A$ in a given subset of $\mathbb {Z}_+^d$. More concretely, consider a non-empty set ${\mathcal {X}}\subseteq \mathbb {Z}_+^d$. We will say that a set $\Gamma \subseteq {\mathcal {X}}$ is **increasing** in ${\mathcal {X}}$ with respect to $\preccurlyeq _A$ if for every $x \in \Gamma$ and $y \in {\mathcal {X}}$, $x \preccurlyeq _A y$ implies that $y \in \Gamma$. We observe that, for $x \in {\mathcal {X}}$, the set

7 $$\begin{aligned} (K_A +x) \cap {\mathcal {X}}= \{ y \in {\mathcal {X}}\,|\, x \preccurlyeq _A y \} \end{aligned}$$

is increasing in ${\mathcal {X}}$ by the transitivity property of $\preccurlyeq _A$. On the other hand, we will say that a set $\Gamma \subseteq {\mathcal {X}}$ is **decreasing** in ${\mathcal {X}}$ with respect to $\preccurlyeq _A$ if for every $x \in \Gamma$ and $y \in {\mathcal {X}}$, $y \preccurlyeq _A x$ implies that $y \in \Gamma$. We will say that a point *x* is **maximal** (resp. **minimal**) in ${\mathcal {X}}$ if for every $y \in {\mathcal {X}}$, $x \preccurlyeq _A y$ (resp. $y \preccurlyeq _A x$) implies that $x=y$. In this case, the set $\Gamma = \{x\}$ would be increasing (resp. decreasing) in ${\mathcal {X}}$.

#### Remark 3.1

If ${{\,\textrm{rank}\,}}(A)=d$, then the relation $\preccurlyeq _A$ will be antisymmetric and therefore a partial order on $\mathbb {R}^d$. Indeed, if ${{\,\textrm{rank}\,}}(A)=d$, then $A(y-x)=0$ implies that $x=y$. In addition, $\preccurlyeq _A$ will then be a partial order when restricted to ${\mathcal {X}}\subset \mathbb {Z}_+^d$. Throughout this article, we will not assume that ${{\,\textrm{rank}\,}}(A)=d$ and therefore, the relation $\preccurlyeq _A$ might not be a partial order on ${\mathcal {X}}$ (see Examples 4.1, 4.2, and 4.3).

### Stochastic Comparison Theorems

The fundamental objects in the following results are a non-empty set ${\mathcal {X}}\subseteq \mathbb {Z}_+^d$ and a pair of continuous-time Markov chains *X* and $\breve{X}$ with the same state space ${\mathcal {X}}$ and where it is assumed that the set of all possible transition vectors for *X* or $\breve{X}$ is a finite set. We denote the size of this set by *n*. A primary example of this setup is two stochastic chemical reaction networks as described in Sect. 2 with different propensity functions. We will now formally introduce the notation for stating our results.

Consider a non-empty set ${\mathcal {X}}\subseteq \mathbb {Z}_+^d$, an integer $n \ge 1$ and a collection of distinct vectors $v_1,\ldots ,v_n$ in $\mathbb {Z}^d {\setminus } \{0\}$, where 0 is the origin in $\mathbb {Z}^d$. Consider two collections of functions $\Upsilon =(\Upsilon _1, \dots ,\Upsilon _n)$ and $\breve{\Upsilon }= (\breve{\Upsilon }_1, \dots ,\breve{\Upsilon }_n)$ defined on ${\mathcal {X}}$ and taking values in $\mathbb {R}_+$, such that for every $1 \le j \le n$ and $x \in {\mathcal {X}}$:

8 $$\begin{aligned} \text {if } x +v_j \notin {\mathcal {X}}, \text { then } \Upsilon _j(x) = \breve{\Upsilon }_j(x)= 0. \end{aligned}$$

Consider a continuous-time Markov chain *X* on the state space ${\mathcal {X}}$ with infinitesimal generator $Q=(Q_{x,y})_{x,y \in {\mathcal {X}}}$ defined for $x,y \in {\mathcal {X}}$ by

9 $$\begin{aligned} Q_{x,y}:= {\left\{ \begin{array}{ll} \Upsilon _j(x) &{} \text { if } y-x = v_j \text { for some } 1 \le j \le n, \\ - \sum _{j=1}^n\Upsilon _j(x) &{} \text { if } x = y, \\ 0 &{} \text { otherwise. } \end{array}\right. } \end{aligned}$$

Consider the analogous continuous-time Markov chain $\breve{X}$ with infinitesimal generator $\breve{Q}$ as in (9) but with functions $\breve{\Upsilon }_1, \dots ,\breve{\Upsilon }_n$ instead of $\Upsilon _1, \dots ,\Upsilon _n$. We call *X* and $\breve{X}$ the continuous-time Markov chains associated with $\Upsilon$ and $\breve{\Upsilon }$, respectively. We will assume that *X* and $\breve{X}$ do not explode in finite time. The following is our main result.

**Fig. 1:**
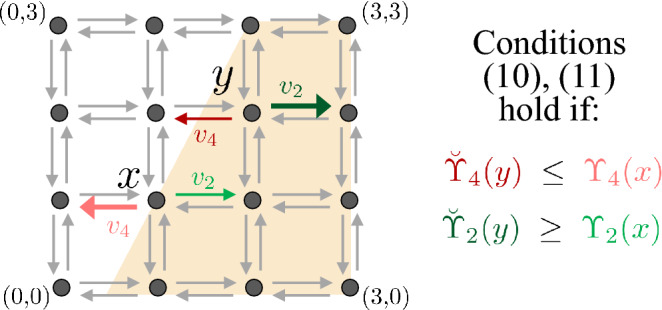
**Pictorial representation of conditions** (10), (11) **for a certain **$(K_{A} + x) \cap {\mathcal {X}}$ **in a two-dimensional lattice.** Here, ${\mathcal {X}}=\{0,1, 2, 3\} \times \{0,1, 2, 3\}$, $n=4$, $v_1=(0,1)^T$, $v_2=(1,0)^T$, $v_3=(0, -1)^T$, $v_4=(-1,0)^T$, where *T* denotes transpose, $A=[2\; -1]$, and $(K_{A} + x) \cap {\mathcal {X}}= \{ w \in {\mathcal {X}} \,|\, [2\; -1](w - x) \ge 0 \}$. In the graph, $(K_{A} + x) \cap {\mathcal {X}}$ consists of the states (black dots) that lie in the light orange region and the arrows represent possible transitions along $v_1,v_2,v_3,v_4$ between states. For the exhibited states $x,y \in {\mathcal {X}}$ with $x \preccurlyeq _A y$, the light green (dark green) and light red (dark red) arrows represent the transitions with rates $\Upsilon _2(x)$ ($\breve{\Upsilon }_2 (y)$) and $\Upsilon _4(x)$ ($\breve{\Upsilon }_4 (y)$) for the Markov chain *X* ($\breve{X}$). Higher transitions rates are associated with thicker arrows. To check the conditions (10) and (11), since $y+v_4 \notin K_A + x$ and $y \notin K_A +x+v_2$, we need to check that $\breve{\Upsilon }_4(y) \le \Upsilon _4(x)$ and $\breve{\Upsilon }_2(y) \ge \Upsilon _2(x)$

#### Theorem 3.1

Consider a non-empty set ${\mathcal {X}}\subseteq \mathbb {Z}_+^d$, a collection of distinct vectors $v_1,\ldots ,v_n$ in $\mathbb {Z}^d {\setminus } \{0\}$ and two collections of nonnegative functions on ${\mathcal {X}}$, $\Upsilon =(\Upsilon _1, \dots ,\Upsilon _n)$ and $\breve{\Upsilon }= (\breve{\Upsilon }_1, \dots ,\breve{\Upsilon }_n)$, such that (8) holds and the associated continuous-time Markov chains do not explode in finite time. Consider a matrix $A \in \mathbb {R}^{m \times d}$ with nonzero rows and suppose that for every $x,y \in {\mathcal {X}}$ such that $x \preccurlyeq _A y$ the following hold:

10 $$\begin{aligned} \breve{\Upsilon }_j(y) \le \Upsilon _j(x), \quad \text {for each } 1 \le j \le n \text { such that } y +v_j \in {\mathcal {X}}\setminus (K_A +x), \end{aligned}$$

and

10 $$\begin{aligned} \breve{\Upsilon }_j(y)\ge \Upsilon _j(x), \quad \text {for each } 1 \le j \le n \text { such that } x +v_j \in {\mathcal {X}}\text { and } y \notin K_A + x + v_j.\nonumber \\ \end{aligned}$$

Then, for each pair $x^{\circ },\breve{x}^\circ \in {\mathcal {X}}$ such that $x^{\circ }\preccurlyeq _A \breve{x}^\circ$, there exists a probability space $(\Omega ,{\mathcal {F}},\mathbb {P})$ with two continuous-time Markov chains $X = \{X(t), \, t \ge 0\}$ and $\breve{X}=\{\breve{X}(t), \, t \ge 0\}$ defined there, each having state space ${\mathcal {X}}\subseteq \mathbb {Z}^d_+$, with infinitesimal generators *Q* and $\breve{Q}$, associated with $\Upsilon$ and $\breve{\Upsilon }$, respectively, with initial conditions $X(0)=x^{\circ }$ and $\breve{X}(0)=\breve{x}^\circ$ and such that:

11 $$\begin{aligned} \mathbb {P}\left[ X(t) \preccurlyeq _A \breve{X}(t) \text { for every } t \ge 0 \right] =1. \end{aligned}$$

An example of checking conditions (10) and (11) is given in Fig. 1. The proof of Theorem 3.1 is given in Sect. 5.1. The main idea in the construction of the processes *X* and $\breve{X}$ is *uniformization* (see Chapter 2 in Keilson (1979)) together with a suitable coupling. Our proof uses a single Poisson process together with a sequence of i.i.d. uniform random variables to determine potential jumps for the two continuous-time Markov chains, where for *X* and $\breve{X}$, potential jumps in the same direction $v_j$ are coupled together, and their probabilities of acceptance are given by normalized versions of their infinitesimal transition rates $\Upsilon _j$ and $\breve{\Upsilon }_j$. Uniformization can be done provided the diagonal terms of the infinitesimal generators are uniformly bounded in size. In the proof of Theorem 3.1, we initially make this assumption on *Q* and $\breve{Q}$ in order to construct *X* and $\breve{X}$. We then generalize the result to Markov chains that do not explode in finite time by using a truncation and limiting procedure. The construction mentioned here, for the case where the diagonal terms of the infinitesimal generators are uniformly bounded in size, besides playing a key role in our proofs, is also the basis for an algorithm described in SI - Section S.4, which provides a way to simultaneously simulate the processes *X* and $\breve{X}$ in a comparable manner.

#### Remark 3.2

In Theorem 5.3 of Massey (1987), the author provides a necessary and sufficient condition for stochastic comparison of continuous-time Markov chains at each fixed time for all partially ordered initial conditions. By the work of Kamae et al. (1977), the conditions in Massey (1987) imply the existence of a coupling of continuous-time Markov chains so that a relation such as (12) holds. Massey’s condition requires that $\sum _{w \in \Gamma } Q_{xw} \le \sum _{w \in \Gamma }\breve{Q}_{yw}$ for every $x \preccurlyeq _A y$ and every set $\Gamma \subseteq {\mathcal {X}}$ that is increasing in ${\mathcal {X}}$ with respect to $\preccurlyeq _A$ and such that either $x \in \Gamma$ or $y \notin \Gamma$. These inequalities can often be difficult to check since first, they involve computing sums of terms in the infinitesimal generators and second, the form of all increasing sets can be hard to determine. In Theorem 3.1 we overcome these obstacles by providing simplified sufficient conditions that involve only pointwise comparison of entries in the infinitesimal generators associated to each of the transition vectors $v_j$. Besides this practical value, in our context, our results go beyond the work of Massey (1987), since he assumes that $\preccurlyeq _A$ is a partial order (we only assume preorder) and he assumes that the diagonal entries of the infinitesimal generators are bounded (we generalize to non-exploding Markov chains). Our proof has a commonality with the work of Massey in the sense that we also use uniformization. It is different in the sense that, when infinitesimal transition rates are bounded, we construct an explicit coupling for all time, exploiting the simplified nature of our conditions, while Massey does not provide an explicit coupling. Instead, he proves existence of a stochastic comparison for each fixed time, using a semigroup approach.

Conditions (10) and (11) may be simplified if we consider a particular relation between the matrix *A* and the vectors $v_1,\ldots ,v_n$ in which $A \in \mathbb {Z}^{m \times d}$ and $Av_j$ has entries taking values only in $\{-1,0,1\}$ for every $1\le j \le n$. More concretely, let us consider a class of continuous-time Markov chains such that, for a given matrix *A* with nonzero rows, if the Markov chain starts within the set $K_A + x$, then to go outside of it, the process will necessarily hit its boundary, and similarly for entry into $K_A + x$. In this case, we can derive a theorem whose conditions must be checked only on the boundary of $K_A + x$ because the only transitions that can lead the Markov chain outside or inside the set $K_A + x$ are ones starting on the boundary of $K_A + x$. Before stating the theorem, let us introduce the sets $\partial _i(K_A+x):= \{ y \in K_A +x \,|\, \langle A_{i\bullet },y\rangle = \langle A_{i\bullet },x\rangle \}$,^2^ for each $1 \le i \le m$. We can then characterize^3^ the boundary of $K_A + x$ as follows:

11 $$\begin{aligned} \partial (K_A +x) = \bigcup _{i=1}^{m} \partial _i(K_A+x). \end{aligned}$$

#### Theorem 3.2

Consider a non-empty set ${\mathcal {X}}\subseteq \mathbb {Z}_+^d$, a collection of distinct vectors $v_1,\ldots ,v_n$ in $\mathbb {Z}^d \setminus \{0\}$ and two collections of nonnegative functions on ${\mathcal {X}}$, $\Upsilon =(\Upsilon _1, \dots ,\Upsilon _n)$ and $\breve{\Upsilon }= (\breve{\Upsilon }_1, \dots ,\breve{\Upsilon }_n)$ such that (8) holds and the associated continuous-time Markov chains do not explode in finite time. Consider a matrix $A \in \mathbb {Z}^{m \times d}$ with nonzero rows and suppose that both of the following conditions hold:

(i) For each $1\le j \le n$, the vector $Av_j$ has entries in $\{-1,0,1\}$ only.
(ii) For each $x \in {\mathcal {X}}$, $1 \le i \le m$ and $y \in \partial _i(K_A+x) \cap {\mathcal {X}}$ we have that
  12 $$\begin{aligned} \breve{\Upsilon }_j(y) \le \Upsilon _j(x), \quad \text {for each } 1 \le j \le n \text { such that } \langle A_{i\bullet },v_j\rangle < 0, \end{aligned}$$
  and
  12 $$\begin{aligned} \breve{\Upsilon }_j(y) \ge \Upsilon _j(x), \quad \text {for each } 1 \le j \le n \text { such that } \langle A_{i\bullet },v_j\rangle > 0. \end{aligned}$$

Then, for each pair $x^{\circ },\breve{x}^\circ \in {\mathcal {X}}$ such that $x^{\circ }\preccurlyeq _A \breve{x}^\circ$, there exists a probability space $(\Omega ,{\mathcal {F}},\mathbb {P})$ with two continuous-time Markov chains $X = \{X(t), \, t \ge 0\}$ and $\breve{X}=\{\breve{X}(t), \, t \ge 0\}$ defined there, each having state space ${\mathcal {X}}\subseteq \mathbb {Z}^d_+$, with infinitesimal generators given by *Q* and $\breve{Q}$, associated with $\Upsilon$ and $\breve{\Upsilon }$, respectively, with initial conditions $X(0)=x^{\circ }$ and $\breve{X}(0)=\breve{x}^\circ$, and such that:

13 $$\begin{aligned} \mathbb {P}\left[ X(t) \preccurlyeq _A \breve{X}(t) \text { for every } t \ge 0 \right] =1. \end{aligned}$$

The proof of this theorem is given in Sect. 5.2 and involves checking that (10) and (11) of Theorem 3.1 hold, using conditions *(i) *and *(ii) *of Theorem 3.2.

#### Remark 3.3

In the context of Theorem 3.2, it is possible that for $x \in {\mathcal {X}}$, and $y \in \partial _{i_1}(K_A+x) \cap \partial _{i_2}(K_A+x) \cap {\mathcal {X}}$ with $i_1 \ne i_2$, it happens that $\langle A_{i_1\bullet },v_j\rangle < 0$ and $\langle A_{i_2\bullet },v_j\rangle > 0$ for some $1 \le j \le n$. For condition (*ii*) to hold, we must then have $\breve{\Upsilon }_j(y) = \Upsilon _j(x)$.

When there are multiple vectors $v_j$ with a common value for $Av_j$, the pointwise comparison in *j*, for $1 \le j \le n$, in conditions (14) and (15) in Theorem 3.2, can be weakened. To this end, let us introduce the set of distinct vectors $\{\eta ^1,\dots ,\eta ^s\}$ formed by $Av_j$, for $1 \le j \le n$, where *s* denotes the cardinality of this set. Consider the subsets of indices

14 $$\begin{aligned} G^{k}:= \{ j \,|\, 1\le j \le n \hbox { and } Av_j =\eta ^k \}, \quad \text {for } 1 \le k \le s. \end{aligned}$$

Then we have the following theorem.

#### Theorem 3.3

Consider a non-empty set ${\mathcal {X}}\subseteq \mathbb {Z}_+^d$, a collection of distinct vectors $v_1,\ldots ,v_n$ in $\mathbb {Z}^d {\setminus } \{0\}$ and two collections of nonnegative functions on ${\mathcal {X}}$, $\Upsilon =(\Upsilon _1, \dots ,\Upsilon _n)$ and $\breve{\Upsilon }= (\breve{\Upsilon }_1, \dots ,\breve{\Upsilon }_n)$ such that (8) holds and the associated continuous-time Markov chains do not explode in finite time. Consider a matrix $A \in \mathbb {Z}^{m \times d}$ with nonzero rows and suppose that both of the following conditions hold:

(i) For each $1\le j \le n$, the vector $Av_j$ has entries in $\{-1,0,1\}$ only.
(ii) For each $x \in {\mathcal {X}}$, $1 \le i \le m$ and $y \in \partial _i(K_A+x) \cap {\mathcal {X}}$ we have that
  14 $$\begin{aligned} \sum _{j \in G^{k}} \breve{\Upsilon }_j(y) \le \sum _{j \in G^{k}} \Upsilon _j(x), \quad \text {for each } k \text { such that } \eta ^k_i <0, \end{aligned}$$
  and
  15 $$\begin{aligned} \sum _{j \in G^{k} } \breve{\Upsilon }_j(y) \ge \sum _{j \in G^{k}} \Upsilon _j(x), \quad \text {for each } k \text { such that } \eta ^k_i >0. \end{aligned}$$

Then, for each pair $x^{\circ },\breve{x}^\circ \in {\mathcal {X}}$ such that $x^{\circ }\preccurlyeq _A \breve{x}^\circ$, there exists a probability space $(\Omega ,{\mathcal {F}},\mathbb {P})$ with two continuous-time Markov chains $X = \{X(t), \, t \ge 0\}$ and $\breve{X}=\{\breve{X}(t), \, t \ge 0\}$ defined there, each having state space ${\mathcal {X}}\subseteq \mathbb {Z}^d_+$, with infinitesimal generators *Q* and $\breve{Q}$, associated with $\Upsilon$ and $\breve{\Upsilon }$, respectively, with initial conditions $X(0)=x^{\circ }$ and $\breve{X}(0)=\breve{x}^\circ$ and such that:

15 $$\begin{aligned} \mathbb {P}\left[ X(t) \preccurlyeq _A \breve{X}(t) \text { for every } t \ge 0 \right] =1. \end{aligned}$$

The proof of this theorem is given in Sect. 5.3.

#### Remark 3.4

If $\Upsilon = \breve{\Upsilon }$, Theorems 3.1, 3.2 and 3.3 give sufficient conditions for monotonic dependence of the stochastic dynamic behavior on the initial condition. In the sense of Massey (1987), this notion corresponds to constructing a *strongly monotone Markov chain*.

#### Remark 3.5

For *deterministic* dynamical systems, there is a considerable literature giving monotonicity conditions with respect to initial conditions (see, e.g., Hirsch and Smith (2006)). Furthermore, Angeli and Sontag (2003) extended the concept of monotone systems to systems having external inputs (i.e., $\dot{x} = f(x,u)$, with *x* representing the state and *u* representing the input). More precisely, they developed tools to prove monotonic dependence of the deterministic dynamic behavior on the initial condition and external input, provided that certain sign conditions on the first partial derivatives of the function *f*(*x*, *u*) are satisfied on the entire state and input space. These theoretical tools can be used also to study how changing a system parameter affects the deterministic behavior of the system, by viewing *u* as the system parameter of interest.

#### Remark 3.6

Checking the conditions in Theorems 3.2 and 3.3 (if they hold) is less cumbersome than checking the conditions in Theorem 3.1. In fact, compared to Theorem 3.1, for Theorems 3.2 and 3.3, the conditions must be checked only on the boundaries of $K_A+x$, given that condition (*i*) there is assumed to hold. Furthermore, Theorem 3.3 has less restrictive conditions (i.e., comparing sums of infinitesimal rates associated with transitions inward or outward with respect to the hyperplanes $\{ z \in \mathbb {R}^d \,|\,\langle A_{i\bullet },z\rangle =\langle A_{i\bullet },x\rangle = \langle A_{i\bullet },y\rangle \}$, $1 \le i \le m$, instead of comparing transition rates one-by-one for $1 \le j \le n$).

### Monotonicity Properties for (Mean) First Passage Times and Stationary Distributions

The first consequence of our main results is for first passage times and it is related to stochastic orderings of real-valued random variables. Let *Y* and *Z* be $[0,\infty ]$-valued random variables with cumulative distribution functions $F_Y$ and $F_Z$, respectively. We say that *Y* is smaller than *Z* in the **usual stochastic order**, written $Y \preccurlyeq _{st} Z$ if $F_Y(t) \ge F_Z(t)$ for every $t \in \mathbb {R}$. The relation $Y \preccurlyeq _{st} Z$ is equivalent to the existence of a probability space $(\Omega ,{\mathcal {F}},\mathbb {P})$ with random variables $Y' \overset{\text {dist}}{=} Y$ and $Z' \overset{\text {dist}}{=} Z$ defined there such that $\mathbb {P}(Y' \le Z') =1$. The reader may consult Chapter 1 in Muller and Stoyan (2002) for the corresponding proofs and further properties of this notion.

#### Theorem 3.4

Consider a non-empty set ${\mathcal {X}}\subseteq \mathbb {Z}_+^d$, a collection of distinct vectors $v_1,\ldots ,v_n$ in $\mathbb {Z}^d {\setminus } \{0\}$ and two collections of nonnegative functions on ${\mathcal {X}}$, $\Upsilon =(\Upsilon _1, \dots ,\Upsilon _n)$ and $\breve{\Upsilon }= (\breve{\Upsilon }_1, \dots ,\breve{\Upsilon }_n)$, such that (8) holds and the associated continuous-time Markov chains do not explode in finite time. Consider a matrix $A \in \mathbb {R}^{m \times d}$ with nonzero rows and suppose that at least one of the following holds:

(i) For every $x,y \in {\mathcal {X}}$ such that $x \preccurlyeq _A y$, conditions (10) and (11) are satisfied.
(ii) The matrix *A* has integer-valued entries and conditions (*i*) and (*ii*) in Theorem 3.2 are satisfied.
(iii) The matrix *A* has integer-valued entries and conditions (*i*) and (*ii*) in Theorem 3.3 are satisfied.

Let $x^{\circ },\breve{x}^\circ \in {\mathcal {X}}$ be such that $x^{\circ }\preccurlyeq _A \breve{x}^\circ$ and let $X = \{X(t), \, t \ge 0\}$ and $\breve{X}=\{\breve{X}(t), \, t \ge 0\}$ be two continuous-time Markov chains (possibly defined on different probability spaces), each having state space ${\mathcal {X}}\subseteq \mathbb {Z}^d_+$, with infinitesimal generators *Q* and $\breve{Q}$, associated with $\Upsilon$ and $\breve{\Upsilon }$, respectively, and with initial conditions $X(0)=x^{\circ }$ and $\breve{X}(0)=\breve{x}^\circ$. For a non-empty set $\Gamma \subseteq {\mathcal {X}}$, consider $T_{\Gamma }:= \inf \{ t \ge 0 \,|\, X(t) \in \Gamma \}$ and $\breve{T}_{\Gamma }:= \inf \{ t \ge 0 \,|\, \breve{X}(t) \in \Gamma \}$. If $\Gamma$ is increasing in ${\mathcal {X}}$ with respect to the relation $\preccurlyeq _A$, then

16 $$\begin{aligned} \breve{T}_{\Gamma } \preccurlyeq _{st} T_\Gamma , \end{aligned}$$

and the mean first passage time of $\breve{X}$ from $\breve{x}^\circ$ to $\Gamma$ is dominated by the mean first passage time of *X* from $x^{\circ }$ to $\Gamma$. If $\Gamma$ is decreasing in ${\mathcal {X}}$ with respect to the relation $\preccurlyeq _A$, then

16 $$\begin{aligned} T_\Gamma \preccurlyeq _{st} \breve{T}_{\Gamma }, \end{aligned}$$

and the mean first passage time of *X* from $x^{\circ }$ to $\Gamma$ is dominated by the mean first passage time of $\breve{X}$ from $\breve{x}^\circ$ to $\Gamma$.

#### Proof

By Theorem 3.1, 3.2 or 3.3, we can construct two versions of the processes *X* and $\breve{X}$ on a common probability space $(\Omega ,{\mathcal {F}},\mathbb {P})$ with initial conditions $x^{\circ }$ and $\breve{x}^\circ$, respectively, and such that (12) or (16) or (20) hold. We denote these versions again by *X* and $\breve{X}$, and we observe that to show (21), it suffices to show that for an increasing set $\Gamma$, $\mathbb {P}[\breve{T}_{\Gamma } \le T_\Gamma ]=1$ for $T_{\Gamma }$ and $\breve{T}_{\Gamma }$ associated with these versions of *X* and $\breve{X}$. To see that this holds, let ${\tilde{\Omega }}$ be a set of probability one on which

17 $$\begin{aligned} X(t) \preccurlyeq _A \breve{X}(t), \quad \text { for all } t \ge 0 \end{aligned}$$

(this exists by (12), (16) or (20)). On $\{T_{\Gamma }=+\infty \}$, it is clear that $\breve{T}_{\Gamma } \le T_{\Gamma }$. For each $\omega \in \{T_{\Gamma } < + \infty \} \cap {\tilde{\Omega }}$ and $\varepsilon > 0$ there is $\tau _{\varepsilon }(\omega ) \in [T_{\Gamma }(\omega ),T_{\Gamma }(\omega ) + \varepsilon )$ such that $X(\tau _{\varepsilon }(\omega )) \in \Gamma$ and by (23), $X(\tau _{\varepsilon }(\omega )) \preccurlyeq _A \breve{X}(\tau _{\varepsilon }(\omega ))$. And then, since $\Gamma$ is increasing, $\breve{X}(\tau _{\varepsilon }(\omega )) \in \Gamma$. It follows that $\breve{T}_{\Gamma }(\omega ) \le T_{\Gamma }(\omega ) + \varepsilon$ and letting $\varepsilon \rightarrow 0$ we obtain that $\breve{T}_{\Gamma }(\omega ) \le T_{\Gamma }(\omega )$. It follows that $\mathbb {P}[\breve{T}_{\Gamma } \le T_\Gamma ]=1$. For the result on mean first passage times, let ${\overline{F}}_{T_{\Gamma }}:= 1 - F_{T_\Gamma }$ and ${\overline{F}}_{\breve{T}_{\Gamma }}:= 1 - F_{\breve{T}_\Gamma }$ represent the complementary cumulative distribution functions for $T_{\Gamma }$ and $\breve{T}_{\Gamma }$, respectively. Observe that (21) implies that ${\overline{F}}_{\breve{T}_{\Gamma }} \le {\overline{F}}_{T_\Gamma }$. For a nonnegative random variable, the mean of the random variable is given by the Lebesgue integral of the complementary cumulative distribution function. Consequently, the mean first passage time for $\breve{X}$ from $\breve{x}^\circ$ to $\Gamma$ is given by $\int _{0}^{\infty } {\overline{F}}_{\breve{T}_{\Gamma }}(t)dt \le \int _0^{\infty } {\overline{F}}_{T_\Gamma }(t) dt$, where the latter is the mean first passage time for *X* from $x^{\circ }$ to $\Gamma$. If $\Gamma$ is decreasing, analogous arguments yield the results stated for that case. $\square$

The second consequence of our results provides a comparison result for stationary distributions.

#### Theorem 3.5

Consider a non-empty set ${\mathcal {X}}\subseteq \mathbb {Z}_+^d$, a collection of distinct vectors $v_1,\ldots ,v_n$ in $\mathbb {Z}^d {\setminus } \{0\}$ and two collections of nonnegative functions on ${\mathcal {X}}$, $\Upsilon =(\Upsilon _1, \dots ,\Upsilon _n)$ and $\breve{\Upsilon }= (\breve{\Upsilon }_1, \dots ,\breve{\Upsilon }_n)$, such that (8) holds and the associated continuous-time Markov chains do not explode in finite time. Consider a matrix $A \in \mathbb {R}^{m \times d}$ with nonzero rows and suppose that at least one of the following holds:

(i) For every $x,y \in {\mathcal {X}}$ such that $x \preccurlyeq _A y$, conditions (10) and (11) are satisfied.
(ii) The matrix *A* has integer-valued entries and conditions (*i*) and (*ii*) in Theorem 3.2 are satisfied.
(iii) The matrix *A* has integer-valued entries and conditions (*i*) and (*ii*) in Theorem 3.3 are satisfied.

Assume that the two continuous-time Markov chains on the set ${\mathcal {X}}$ with infinitesimal generators *Q* and $\breve{Q}$, associated with $\Upsilon$ and $\breve{\Upsilon }$, respectively, are irreducible and positive recurrent on ${\mathcal {X}}$, and denote the associated stationary distributions by $\pi$ and $\breve{\pi }$, respectively. If $\Gamma \subseteq {\mathcal {X}}$ is a non-empty set that is increasing in ${\mathcal {X}}$ with respect to $\preccurlyeq _A$, then

18 $$\begin{aligned} \sum _{x \in \Gamma } \pi _x \le \sum _{x \in \Gamma } \breve{\pi }_x. \end{aligned}$$

If $\Gamma \subseteq {\mathcal {X}}$ is a non-empty set that is decreasing in ${\mathcal {X}}$ with respect to $\preccurlyeq _A$, then

18 $$\begin{aligned} \sum _{x \in \Gamma } \breve{\pi }_x \le \sum _{x \in \Gamma } \pi _x. \end{aligned}$$

#### Proof

As in the proof of Theorem 3.4, we can construct two versions of the processes *X* and $\breve{X}$ on a common probability space $(\Omega ,{\mathcal {F}},\mathbb {P})$ for some pair of initial conditions $x^{\circ }\preccurlyeq _A \breve{x}^\circ$. If $\Gamma \subseteq {\mathcal {X}}$ is increasing, equation (12) or (16) or (20) yields that $\mathbb {P}(X(t) \in \Gamma ) \le \mathbb {P}(\breve{X}(t) \in \Gamma )$ for every $t \ge 0$. By letting $t \rightarrow \infty$ and observing that the stationary distribution is the steady-state distribution under our assumptions of irreducibility and positive recurrence, we obtain (24). If $\Gamma$ is decreasing, an analogous argument yields (25). $\square$

#### Remark 3.7

A special case of Theorems 3.4 and 3.5 is when $\Gamma = \{x\}$ for some maximal or minimal element $x \in {\mathcal {X}}$.

In the next section, we give examples which illustrate Theorem 3.2 (see Examples 4.1, 4.2, 4.4 and 4.5), Theorem 3.3 (see Example 4.3), Theorem 3.4 and Theorem 3.5 for continuous-time Markov chains that are stochastic chemical reaction networks. For Examples 4.1, 4.2 and 4.3, the state space ${\mathcal {X}}$ will be a stoichiometric compatibility class $z + {\mathcal {L}}$ intersected with $\mathbb {Z}^d_+$. For Examples 4.4 and 4.5, we work with reduced Markov chains and the state space ${\mathcal {X}}$ will be a projection of a suitable higher-dimensional stoichiometric compatibility class $z + {\mathcal {L}}$ intersected with $\mathbb {Z}^d_+$.

## Examples

In this section, we apply the theoretical tools developed in the paper to several examples. While in Examples 4.1, 4.3 and 4.4 the Markov chains analyzed have a finite state space, in Examples 4.2 and 4.5 the Markov chains have a countably infinite state space, but it is straightforward to verify that they do not explode (see SI - Sections S.1.2 and S.1.3, respectively). The choice of matrix *A* in each example is based on the specific monotonicity relationship of interest. While for simpler cases the choice of *A* is straightforward, for more complicated systems the choice can be more subtle. In many cases, in order to study the monotonicity properties for the stochastic behavior of our system, we can rely on Theorem 3.2, which provides a reasonable approach to narrow down the choices for suitable *A*. The approach consists in solving, for each row *i*, the system of equations $\sum _{k=1}^d A_{ik}(v_j)_k=b_{ij}$, with $b_{ij}$ equal to $1,-1,$ or 0 depending, based on the monotonicity relationship of interest, whether we expect that the Markov chain transition in the direction $v_j$ leads inside, outside, or is parallel to the boundary of the region $K_A+x$. Finally, it is worth noticing that, while all the following examples compare two identical reaction networks with different rate constants, our theory can also be applied to compare two different reaction networks as long as they have the same reaction vectors $\{v_j\}_{j=1}^n$.

### Example 4.1

**Enzyme kinetics I**

**Fig. 2:**
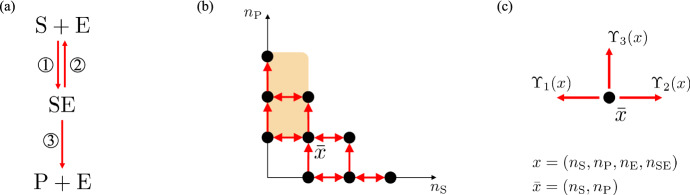
**Reaction model and corresponding Markov chain for enzymatic kinetics I example.** **a** Chemical reaction system. The numbers on the arrows correspond to the associated reactions. **b** Projected Markov chain graph for one stoichiometric compatibility class with two conservation laws $n_{\textrm{S}}+n_{\textrm{P}}+n_{\textrm{SE}}=\mathrm {S_{tot}}=3$ and $n_{\textrm{E}}+n_{\textrm{SE}}=\mathrm {E_{tot}}=2$. The projection takes a state $x=(n_{\textrm{S}},n_{\textrm{P}},n_{\textrm{E}},n_{\textrm{SE}})=(n_{\textrm{S}},n_{\textrm{P}},\mathrm {E_{tot}}-\mathrm {S_{tot}}+n_{\textrm{S}}+n_{\textrm{P}},\mathrm {S_{tot}}-n_{\textrm{S}}-n_{\textrm{P}})$ to ${\bar{x}} = (n_{\textrm{S}},n_{\textrm{P}})$. We use black dots to represent the states, red double-ended arrows to represent transitions in both directions and red single-ended arrows to represent transitions in one direction. Note that ${\bar{x}} = (0,0)$ is not a vertex in the graph because $0 \le n_{\textrm{E}} = \mathrm {E_{tot}}-\mathrm {S_{tot}}+n_{\textrm{S}}+n_{\textrm{P}}$, and so $n_{\textrm{S}}+n_{\textrm{P}}\ge 3-2=1$. We use orange to highlight the projection of the region $K_A+x$ intersected with the stoichiometric compatibility class, where *A* is defined in (27). **c** The projections of the directions of the possible transitions of the Markov chain. The transition rates $\Upsilon _1(x)$, $\Upsilon _2(x)$, and $\Upsilon _3(x)$ are defined in (26)

Let us consider a classic model of enzyme kinetics (see Michaelis and Menten (1913) and Kang et al. (2019)), where an enzyme catalyzes the conversion of a substrate to a product. The species considered here are substrate (S), enzyme (E), intermediate enzyme-substrate complex (SE), and product (P), and the chemical reaction system is depicted in Fig. 2a. We are interested in how the rate constant $\kappa _3$ affects the time to convert the substrate to the final product.

To this end, let us first introduce the set of species ${\mathscr {S}}=\{\textrm{S},\textrm{P},\textrm{E},\textrm{SE}\}$, and the set of reactions ${\mathscr {R}}=\{(v^-_1,v^+_1),(v^-_2,v^+_2),(v^-_3,v^+_3)\}$, where $v^-_1= v^+_2= (1,0,1,0)^T$, $v^+_1=v^-_2=v^-_3=(0,0,0,1)^T$, $v^+_3=(0,1,1,0)^T$, where *T* denotes transpose. At a given time, let the counts of each of the species S, P, E and SE be denoted by $n_{\textrm{S}}$, $n_{\textrm{P}}$, $n_{\textrm{E}}$ and $n_{\textrm{SE}}$, respectively. The state of the associated Markov chain is $(n_{\textrm{S}},n_{\textrm{P}},n_{\textrm{E}},n_{\textrm{SE}})$. The potential transitions of the Markov chain are in three possible directions:

$$\begin{aligned} v_1= & {} v^+_1-v^-_1= (-1,0,-1,1)^T,\\ v_2= & {} v^+_2-v^-_2 = (1,0,1,-1)^T,\\ v_3= & {} v^+_3-v^-_3=(0,1,1,-1)^T. \end{aligned}$$

Fixing integers $\mathrm {S_{tot}},\mathrm {E_{tot}}>0$, we have a stoichiometric compatibility class $z+{\mathcal {L}}$ with $z = (\mathrm {S_{tot}},0,\mathrm {E_{tot}},0)$ and ${\mathcal {L}}:= {{\,\textrm{span}\,}}\{v_1,v_2,v_3\}$, which is contained in a two-dimensional affine subspace of four-dimensional space. Then, the state space of the Markov chain is

$$\begin{aligned} {\mathcal {X}}= (z+{\mathcal {L}}) \cap \mathbb {Z}_+^4 = \{(x_1,x_2,x_3,x_4) \in \mathbb {Z}_+^4 | x_1+x_2+x_4=\mathrm {S_{tot}}, x_3+x_4=\mathrm {E_{tot}} \}. \end{aligned}$$

The two constraints described in the last expression for ${\mathcal {X}}$ characterize the two linearly independent conservation laws for this chemical reaction system: $n_{\textrm{S}}+n_{\textrm{P}}+n_{\textrm{SE}}=\mathrm {S_{tot}}$ and $n_{\textrm{E}}+n_{\textrm{SE}}=\mathrm {E_{tot}}$.

Given a state $x=(x_1,x_2,x_3,x_4) \in {\mathcal {X}}$, following mass-action kinetics, the infinitesimal transition rates are

19 $$\begin{aligned} \begin{aligned}&\Upsilon _1(x)= \kappa _1 x_1 x_3,\;\;\Upsilon _2(x)= \kappa _2 x_4,\;\;&\Upsilon _3(x)= \kappa _3 x_4, \end{aligned} \end{aligned}$$

for constants $\kappa _1,\kappa _2,\kappa _3 > 0$. Here, we have used $\kappa _j$ as an abbreviation for $\kappa _{(v_j^-,v_j^+)}$, $j=1,2,3$. We will use similar abbreviations in the other examples too.

We note that the projected process $(X_1,X_2)(\cdot )$ is still a continuous-time Markov chain, and we could apply our theory to it. However, when the functions $\Upsilon _j$, $j=1,2,3$, are written in terms of these two components, they will have a more complex, non-mass action form. Here we apply our theory directly to our four-dimensional Markov chain. For the purpose of visualization, Fig. 2b shows the two-dimensional projection of the four-dimensional Markov chain graph for one stoichiometric compatibility class. In Examples 4.2 and 4.3, we also analyze Markov chains without projections, and in Examples 4.4 and 4.5, we analyze projected Markov chains.

In order to study how the rate constant $\kappa _3$ affects the time to convert the substrate to the final product, let us define the state $(0,\mathrm {S_{tot}},\mathrm {E_{tot}},0)$ associated with $n_{\textrm{P}}=\mathrm {S_{tot}}$ as *p*, the state $(\mathrm {S_{tot}},0,\mathrm {E_{tot}},0)$ associated with $n_{\textrm{S}}=\mathrm {S_{tot}}$ as *s*, and the mean first passage time to reach the state *p*, starting from *s*, as $\mathbb {E}_{s} [T_p]$. We will verify that the assumptions of Theorems 3.2,  3.4 hold and exploit them to determine how $\kappa _3$ affects $\mathbb {E}_{s} [T_p]$. To this end, define the matrix

19 $$\begin{aligned} A= \begin{bmatrix} -1 &{} 0 &{} 0 &{} 0\\ 0 &{} 1 &{} 0 &{} 0 \end{bmatrix} \end{aligned}$$

and consider the preorder $x \preccurlyeq _A y$, defined by $A(y-x)\ge 0$, and the set $K_A +x = \{ w \in \mathbb {R}^4 \,|\, x \preccurlyeq _A w \}$. Let us also consider the infinitesimal transition rates $\breve{\Upsilon }_1(x),\breve{\Upsilon }_2(x)$ and $\breve{\Upsilon }_3(x)$ defined as for $\Upsilon _1(x),\Upsilon _2(x)$ and $\Upsilon _3(x)$, but with $\breve{\kappa }_1=\kappa _1$, $\breve{\kappa }_2=\kappa _2$, $\breve{\kappa }_3>\kappa _3$ in place of $\kappa _1$, $\kappa _2$, $\kappa _3$, respectively. Condition (*i*) of Theorem 3.2 (i.e., for every $1\le j \le n$, the vector $Av_j$ has entries in $\{-1,0,1\}$) holds since $Av_1=(1,0)^T, Av_2=(-1,0)^T$ and $Av_3=(0,1)^T$. Condition (*ii*) of Theorem 3.2 also holds, as shown in the paragraph below.

**Verification of condition** (*ii*) **of Theorem**3.2. We first consider $x \in {\mathcal {X}}$ and $y \in \partial _1(K_A+x) \cap {\mathcal {X}}$, where

$$\begin{aligned}{} & {} \partial _1(K_A+x) \cap {\mathcal {X}}\\{} & {} \quad = \{ w \in \mathbb {Z}^4_+ \,|\, x_1 = w_1, x_2 \le w_2 \} \cap {\mathcal {X}}\\{} & {} \quad = \{ w \in \mathbb {Z}^4_+ \,|\, x_1 = w_1, x_2 \le w_2, x_1 + x_2 + x_4 = w_1 + w_2 + w_4=\mathrm {S_{tot}}, x_3 + x_4 = w_3 + w_4=\mathrm {E_{tot}} \} \\{} & {} \quad = \{ w \in \mathbb {Z}_+^4 \,|\, x_1 = w_1, x_2 \le w_2, x_3 \le w_3, x_4 \ge w_4, w_1 + w_2 + w_4=\mathrm {S_{tot}}, w_3 + w_4=\mathrm {E_{tot}}\}\\{} & {} \quad = \{ w \in {\mathcal {X}}\,|\, x_1 = w_1, x_2 \le w_2, x_3 \le w_3, x_4 \ge w_4\}. \end{aligned}$$

Since $\langle A_{1\bullet },v_1\rangle =1$, $\langle A_{1\bullet },v_2\rangle =-1$, we need to check that $\Upsilon _1(x)\le \breve{\Upsilon }_1(y)$ and $\Upsilon _2(x)\ge \breve{\Upsilon }_2(y)$. The first inequality holds because $y \in \partial _1(K_A+x) \cap {\mathcal {X}}$ implies $x_1=y_1$ and $x_3 \le y_3$ so that $\Upsilon _1(x) = \kappa _1 x_1 x_3 \le \kappa _1 y_1 y_3 = \breve{\kappa }_1 y_1 y_3 = \breve{\Upsilon }_1(y)$. The second inequality holds because $y \in \partial _1(K_A+x) \cap {\mathcal {X}}$ implies $x_4 \ge y_4$ so that $\Upsilon _2(x) = \kappa _2 x_4 \ge \kappa _2 y_4 = \breve{\kappa }_2 y_4 = \breve{\Upsilon }_2(y)$.

Secondly, we consider $x \in {\mathcal {X}}$, $y \in \partial _2(K_A+x) \cap {\mathcal {X}}= \{ w \in {\mathcal {X}}\,|\, x_1 \ge w_1, x_2 = w_2, x_3 \ge w_3, x_4 \le w_4\}$. Then, since $\langle A_{2\bullet },v_3\rangle =1$, we need to check that $\Upsilon _3(x)\le \breve{\Upsilon }_3(y)$. This holds because $y \in \partial _2(K_A+x) \cap {\mathcal {X}}$ implies $x_4 \le y_4$ so that $\Upsilon _3(x) = \kappa _3 x_4 \le \kappa _3 y_4 \le \breve{\kappa }_3 y_4 = \breve{\Upsilon }_3(y)$.

Since all of the hypotheses of Theorem 3.2 hold, we can conclude that, for each $x^{\circ },\breve{x}^\circ \in {\mathcal {X}}$ with $x^{\circ }\preccurlyeq _A \breve{x}^\circ$, there exists a probability space $(\Omega ,{\mathcal {F}},\mathbb {P})$ with two Markov chains $X = \{X(t), \, t \ge 0\}$ and $\breve{X}=\{\breve{X}(t), \, t \ge 0\}$ associated with $\Upsilon$ and $\breve{\Upsilon }$, respectively, such that $X(0)=x^{\circ }$, $\breve{X}(0)=\breve{x}^\circ$ and

$$\begin{aligned} \mathbb {P}\left[ X(t) \preccurlyeq _A \breve{X}(t) \text { for every } t \ge 0 \right] =1. \end{aligned}$$

Furthermore, applying Theorem 3.4 with the set $\Gamma = \{p\}=\{ (0,\mathrm {S_{tot}},\mathrm {E_{tot}},0) \}$, which is increasing in ${\mathcal {X}}$ with respect to $\preccurlyeq _A$, we see that the mean first passage time from *s* to *p*, $\mathbb {E}_{s} [T_p]$, is a decreasing function of $\kappa _3$.

Because the Markov chain has one absorbing state, *p*, per stoichiometric compatibility class, the stationary distribution on a given stoichiometric compatibility class is trivial, and hence so too are its monotonicity properties.

### Example 4.2

**Enzyme kinetics II**

Let us consider an extension of the enzymatic kinetics model introduced in the previous example, in which the substrate S can enter and leave the system and the product can revert to the substrate. This is a simplified version of the enzymatic kinetics considered by Anderson et al. (2010).

**Fig. 3:**
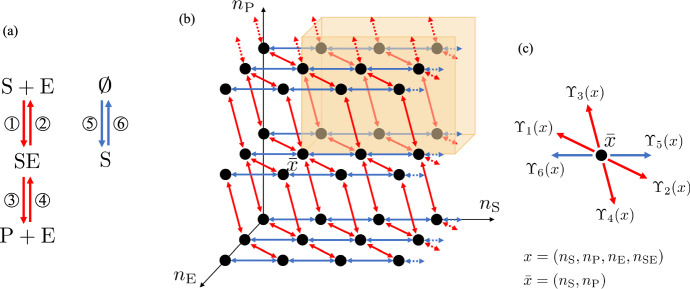
**Reaction model and corresponding Markov chain for enzymatic kinetics II example.** **a** Chemical reaction system. The numbers on the arrows correspond to the associated reactions. **b** Projected Markov chain graph for one stoichiometric compatibility class with the conservation law $n_{\textrm{E}}+n_{\textrm{SE}}=\mathrm {E_{tot}}=2$. The projection takes a state $x=(n_{\textrm{S}},n_{\textrm{P}},n_{\textrm{E}},n_{\textrm{SE}})=(n_{\textrm{S}},n_{\textrm{P}},n_{\textrm{E}},\mathrm {E_{tot}}-n_{\textrm{E}}) \in {\mathcal {X}}$ to ${\bar{x}} = (n_{\textrm{S}},n_{\textrm{P}},n_{\textrm{E}})\in \mathbb {Z}_+^3:0\le n_{\textrm{E}}\le 2$. Here, we use black dots to represent the states, red double-ended arrows to represent transitions in both directions associated with the reactions represented by the red arrows in (**a**) and blue double-ended arrows to represent transitions in both directions associated with the reactions represented by the blue arrows in (**a**). We use dotted arrowed-lines to indicate that the pattern of Markov chain transitions extends to infinity. We use orange to highlight the projections of the region $K_A+x$ intersected with the stoichiometric compatibility class, where *A* is defined in (29). **c** The projections of the directions of the possible transitions of the Markov chain within a stoichiometric compatibility class. The transition rates $\Upsilon _i(x)$, $i=1,2,3,4,5,6$, are defined in (28)

The chemical reaction system is depicted in Fig. 3a.

Now, for this case study, we first determine how the reaction rate constant $\kappa _5$ affects the stochastic behavior of the system and then we will study properties of the system with respect to initial conditions. To this end, let us introduce the set of species ${\mathscr {S}}=\{\textrm{S},\textrm{P},\textrm{E},\textrm{SE}\}$, and, similar to Example 4.1, we let $(n_{\textrm{S}},n_{\textrm{P}},n_{\textrm{E}},n_{\textrm{SE}})$ be the state of the Markov chain that records the number of molecules of each species. The potential transitions of the Markov chain are in six possible directions, $v_j$ for $j=1,...,6$, where $v_1=-v_2=(-1,0,-1,1)^T$, $v_3=-v_4= (0,1,1,-1)^T$, and $v_5=-v_6=(1,0,0,0)^T$ (see SI-Section S.2.1 for the derivation of the $v_j$, $j=1,...,6$). Since there is one linearly independent conservation law in this chemical reaction system: $n_{\textrm{E}}+n_{\textrm{SE}}=\mathrm {E_{tot}}$, each stoichiometric compatibility class is contained in a three-dimensional affine subspace of four-dimensional space, denoted as $z+{\mathcal {L}}$, where $z=(0,0,\mathrm {E_{tot}},0)$ and ${\mathcal {L}}:= {{\,\textrm{span}\,}}\{v_1,v_3,v_5\}$, with fixed integer $\mathrm {E_{tot}}>0$. Then, we can choose the state space of the Markov chain to be ${\mathcal {X}}= (z+{\mathcal {L}}) \cap \mathbb {Z}_+^4 = \{(x_1,x_2,x_3,x_4) \in \mathbb {Z}_+^4 |x_3+x_4=\mathrm {E_{tot}} \}$. Furthermore, given a state $x=(x_1,x_2,x_3,x_4) \in {\mathcal {X}}$, following mass-action kinetics, the associated infinitesimal transition rates are given by

20 $$\begin{aligned} \begin{aligned}&\Upsilon _1(x)= \kappa _1 x_1 x_3,\;\;\Upsilon _2(x)= \kappa _2 x_4,\;\;\Upsilon _3(x)= \kappa _3 x_4,\\&\Upsilon _4(x)= \kappa _4 x_2 x_3,\;\;\Upsilon _5(x)= \kappa _5,\;\;\Upsilon _6(x)= \kappa _6 x_1, \end{aligned} \end{aligned}$$

for $\kappa _1,\kappa _2,\kappa _3,\kappa _4,\kappa _5,\kappa _6 > 0$. As in Example 4.1, we apply our theory directly to our four-dimensional Markov chain, but, for the purpose of illustration, Fig. 3b shows the three-dimensional projection of the Markov chain graph for one stoichiometric compatibility class.

Now, for the first analysis (determining how $\kappa _5$ affects the stochastic behavior of the system), we verify that the assumptions of Theorems 3.2 and 3.5 hold and use them to determine how $\kappa _5$ affects the stationary distribution.

To this end, define the matrix

20 $$\begin{aligned} A= \begin{bmatrix} 1 &{} 0 &{} 0 &{} 0\\ 0 &{} 1 &{} 0 &{} 0\\ 0 &{} 0 &{} -1 &{} 0 \end{bmatrix} \end{aligned}$$

and consider the preorder $x \preccurlyeq _A y$, defined by $A(y-x)\ge 0$. For $x \in {\mathcal {X}}$, $K_A +x = \{ w \in \mathbb {R}^4 \,|\, x \preccurlyeq _A w \}$. Furthermore, let us consider the infinitesimal transition rates $\breve{\Upsilon }_1(x)$, $\breve{\Upsilon }_2(x)$, $\breve{\Upsilon }_3(x)$, $\breve{\Upsilon }_4(x)$, $\breve{\Upsilon }_5(x)$ and $\breve{\Upsilon }_6(x)$ defined as for $\Upsilon _1(x),\Upsilon _2(x),\Upsilon _3(x),\Upsilon _4(x),\Upsilon _5(x)$ and $\Upsilon _6(x)$, but with $\breve{\kappa }_i$ in place of $\kappa _i$, where $\breve{\kappa }_i=\kappa _i$, for $i = 1,2,3,4,6$, and $\breve{\kappa }_5\ge \kappa _5$. Given that $Av_1=(-1,0,1)^T$, $Av_2=(1,0,-1)^T$, $Av_3=(0,1,-1)^T$, $Av_4=(0,-1,1)^T$, $Av_5=(1,0,0)^T$ and $Av_6=(-1,0,0)^T$, we have that condition (*i*) of Theorem 3.2 holds. Condition (*ii*) of that theorem also holds, as shown in the next paragraph.

**Verification of condition** (*ii*) **of Theorem**3.2. First consider $x \in {\mathcal {X}}$ and $y \in \partial _1(K_A+x) \cap {\mathcal {X}}$, where $\partial _1(K_A+x) \cap {\mathcal {X}}= \{ w \in {\mathcal {X}}\,|\, x_1= w_1, x_2\le w_2, x_3\ge w_3, x_4\le w_4 \}$. Since $\langle A_{1\bullet },v_2\rangle =\langle A_{1\bullet },v_5\rangle =1$ and $\langle A_{1\bullet },v_1\rangle =\langle A_{1\bullet },v_6\rangle =-1$, we need to check that $\Upsilon _1(x)\ge \breve{\Upsilon }_1(y),\Upsilon _6(x) \ge \breve{\Upsilon }_6(y),\Upsilon _2(x)\le \breve{\Upsilon }_2(y),$ and $\Upsilon _5(x)\le \breve{\Upsilon }_5(y)$. Given that $y \in \partial _1(K_A+x) \cap {\mathcal {X}}$, the first inequality holds because $\Upsilon _1(x)=\kappa _1 x_1 x_3\ge \kappa _1 y_1 y_3=\breve{\kappa }_1 y_1 y_3= \breve{\Upsilon }_1(y)$, the second inequality holds because $\Upsilon _6(x) =\kappa _6 x_1=\kappa _6 y_1=\breve{\kappa }_6 y_1 =\breve{\Upsilon }_6(y)$, the third inequality holds because $\Upsilon _2(x)=\kappa _2 x_4\le \kappa _2 y_4=\breve{\kappa }_2 y_4=\breve{\Upsilon }_2(y)$, and the fourth inequality holds because $\Upsilon _5(x)= \kappa _5\le \breve{ \kappa }_5=\breve{\Upsilon }_5(y)$.

Secondly, we consider $x \in {\mathcal {X}}$ and $y \in \partial _2(K_A+x) \cap {\mathcal {X}}= \{ w \in {\mathcal {X}}\,|\, x_1\le w_1, x_2=w_2, x_3\ge w_3, x_4\le w_4 \}$. Given that $\langle A_{3\bullet },v_3\rangle =1$ and $\langle A_{3\bullet },v_4\rangle =-1$, we need to check that $\Upsilon _4(x)\ge \breve{\Upsilon }_4(y)$ and $\Upsilon _3(x)\le \breve{\Upsilon }_3(y)$. The first inequality holds because $\Upsilon _4(x)=\kappa _4 x_2 x_3\ge \kappa _4 y_2 y_3=\breve{\kappa }_4 y_2 y_3=\breve{\Upsilon }_4(y)$ and the second inequality holds because $\Upsilon _3(x)=\kappa _3 x_4 \le \kappa _3 y_4 = \breve{\kappa }_3 y_4=\breve{\Upsilon }_3(y)$.

Finally, consider $x \in {\mathcal {X}}$ and $y \in \partial _3(K_A+x) \cap {\mathcal {X}}= \{ w \in {\mathcal {X}}\,|\, x_1\le w_1, x_2 \le w_2, x_3=w_3, x_4= w_4 \}$. Since $\langle A_{3\bullet },v_1\rangle =\langle A_{3\bullet },v_4\rangle =1$ and $\langle A_{3\bullet },v_2\rangle =\langle A_{3\bullet },v_3\rangle =-1$, we need to check that $\Upsilon _2(x)\ge \breve{\Upsilon }_2(y)$, $\Upsilon _3(x)\ge \breve{\Upsilon }_3(y)$, $\Upsilon _1(x) \le \breve{\Upsilon }_1(y)$, and $\Upsilon _4(x)\le \breve{\Upsilon }_4(y)$. Indeed, we have that $\Upsilon _2(x)=\kappa _2 x_4 = \kappa _2 y_4= \breve{\kappa }_2 y_4=\breve{\Upsilon }_2(y)$, $\Upsilon _3(x) = \kappa _3 x_4 = \kappa _3 y_4 =\breve{\kappa }_3 y_4 = \breve{\Upsilon }_3(y)$, $\Upsilon _1(x) = \kappa _1 x_1 x_3 \le \kappa _1 y_1 y_3 = \breve{\kappa }_1 y_1 y_3 = \breve{\Upsilon }_1(y)$, and $\Upsilon _4(x)=\kappa _4 x_2x_3\le \kappa _4 y_2y_3= \breve{\kappa }_4 y_2y_3 = \breve{\Upsilon }_4(y)$.

Thus, all of the hypotheses of Theorem 3.2 are verified, and so, for each pair $x^{\circ },\breve{x}^\circ \in {\mathcal {X}}$ satisfying $x^{\circ }\preccurlyeq _A \breve{x}^\circ$, there exists a probability space $(\Omega ,{\mathcal {F}},\mathbb {P})$ with two Markov chains $X = \{X(t), \, t \ge 0\}$ and $\breve{X}=\{\breve{X}(t), \, t \ge 0\}$ associated with $\Upsilon$ and $\breve{\Upsilon }$, respectively, such that $X(0)=x^{\circ }$, $\breve{X}(0)=\breve{x}^\circ$ and $\mathbb {P}\left[ X(t) \preccurlyeq _A \breve{X}(t) \text { for every } t \ge 0 \right] =1$.

The Markov chains $X,\breve{X}$ are irreducible and positive recurrent (see SI - Section S.1.1).

Furthermore, for the increasing set in ${\mathcal {X}}$ with respect to $\preccurlyeq _A$ defined as $\Gamma (x) = \{ w \in {\mathcal {X}}\,|\, x_1 \le w_1, x_2 \le w_2, x_3 \ge w_3, x_4 \le w_4 \}$, we can apply Theorem 3.5 and obtain that $\sum _{w \in \Gamma (x)} \pi _w \le \sum _{w \in \Gamma (x)} \breve{\pi }_w$.

Loosely speaking, this means that increasing $\kappa _5$ causes the stationary distribution $\pi (x)$ to shift mass toward states characterized by lower $x_3$ and higher $x_1,x_2$ and $x_4$.

For this specific case, in which we have a stochastic chemical reaction network associated with a complex balanced dynamical system, an explicit expression for the stationary distribution can be obtained by applying Theorem 4.1 in Anderson et al. (2010). Analysis of this formula would provide results in agreement with the ones obtained by applying the theoretical tools developed in this paper. Specifically, $\pi _x$ can be written as a product of two Poisson distributions and a binomial distribution, i.e.,

21 $$\begin{aligned} \pi _x=\left( e^{-c_1}\frac{c_1^{x_1}}{x_1!}\right) \left( e^{-c_2}\frac{c_2^{x_2}}{x_2!}\right) \left( \mathrm {E_{tot}}!\frac{c_3^{x_3}}{x_3!}\frac{c_4^{x_4}}{x_4!}\right) ,\;\;\;x\in {\mathcal {X}}, \end{aligned}$$

in which $(c_1,c_2,c_3,c_4)$ represents the complex balanced equilibrium for the deterministic model, where

21 $$\begin{aligned} c_1=\frac{\kappa _5}{\kappa _6},\;\;c_2=\frac{\kappa _1\kappa _3\kappa _5}{\kappa _2\kappa _4\kappa _6},\;\;c_3 =\frac{1}{1+\frac{\kappa _1\kappa _5}{\kappa _2\kappa _6}},\; \textrm{and}\; c_4 =\frac{\frac{\kappa _1\kappa _5}{\kappa _2\kappa _6}}{1+\frac{\kappa _1\kappa _5}{\kappa _2\kappa _6}}. \end{aligned}$$

In most cases, it is not possible to derive an analytical formula for the stationary distribution, but our theorems can still be applied and then monotonicity properties for $\pi$ can still be determined even without an explicit expression for $\pi$. For instance, in the context of the above example, if the infinitesimal transition rates $\Upsilon _i$ do not follow mass-action kinetics, the deficiency zero theorem and Theorem 4.1 in Anderson et al. (2010) do not apply. Nevertheless, our theory can still be easily applied to study monotonicity properties for sample paths and stationary distributions.

As pointed out in Remark 3.4, we can also exploit our theoretical tools to determine monotonicity properties of the system with respect to the initial conditions.

For this, suppose that $\breve{\kappa }_i=\kappa _i$ for $i = 1,2,3,4,5,6$. Then, by the analysis above, Theorem 3.2 holds and yields monotonically (with preorder induced by the matrix *A*) with respect to the initial conditions.

### Example 4.3

**Fig. 4:**
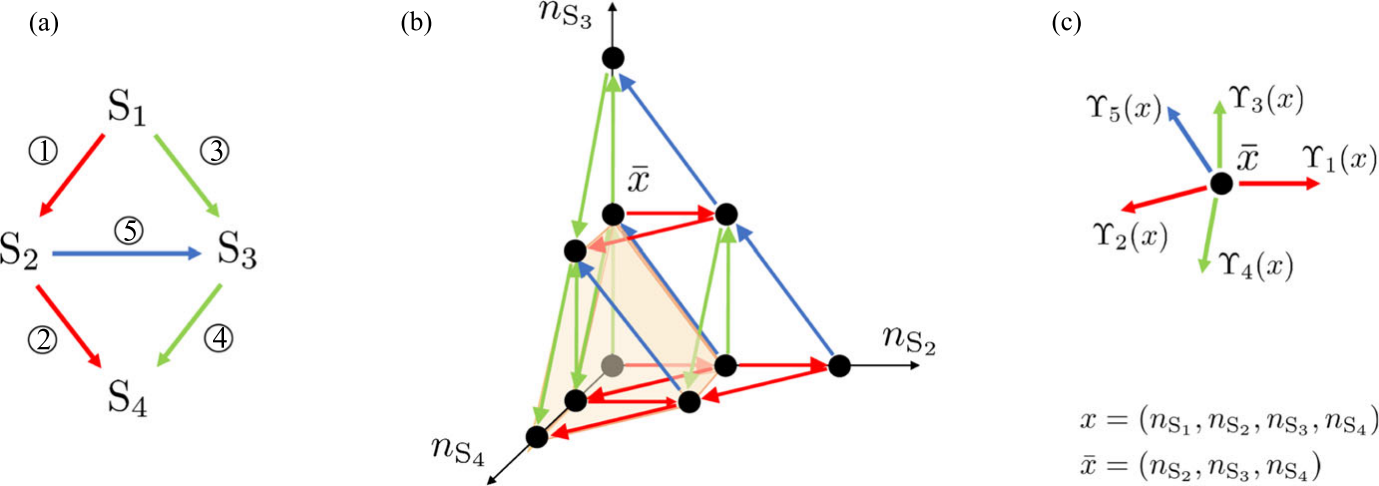
**Circuit inspired by Braess’ paradox and corresponding Markov chain.** **a** Chemical reaction system. The numbers on the arrows correspond to the associated reactions. **b** Projected Markov chain graph for one stoichiometric compatibility class with the conservation law $n_{\mathrm {S_1}}+n_{\mathrm {S_2}}+n_{\mathrm {S_3}}+n_{\mathrm {S_4}}=\mathrm {S_{tot}}=2$. The projection takes a state $x=(n_{\mathrm {S_1}},n_{\mathrm {S_2}},n_{\mathrm {S_3}},n_{\mathrm {S_4}})=(\mathrm {S_{tot}}-n_{\mathrm {S_2}}-n_{\mathrm {S_3}}-n_{\mathrm {S_4}},n_{\mathrm {S_2}},n_{\mathrm {S_3}},n_{\mathrm {S_4}}) \in {\mathcal {X}}$ to ${\bar{x}} = (n_{\mathrm {S_2}},n_{\mathrm {S_3}},n_{\mathrm {S_4}})$. Here, we use black dots to represent the states and red (blue, green) arrows to represent transitions in directions associated with the reactions represented by the red (blue, green) arrows in (**a**). We use orange to highlight the projection of the region $K_A+x$ intersected with the stoichiometric compatibility class, where *A* is defined in (33). **c** The projections of the directions of the possible transitions of the Markov chain within a stoichiometric compatibility class. The transition rates $\Upsilon _i(x)$, $i=1,2,3,4,5$, are given in (32)

**A network topology arising in Braess’ paradox** A natural question in synthetic biology may involve the prediction of whether an engineered biological circuit with additional reactions will lead to the desired effect of accelerating the process or unexpected behaviors. Now, we consider an example inspired by Braess’ paradox, which arises from transportation networks, where adding one or more roads to a road network can slow down overall traffic flow through the network (see Braess (1968) and see also a related state-dependent queuing network model in Calvert et al. (1997)). A simple network of this type is one where there are two routes to get from the start to the final destination, and adding a linkage road between the routes can in some cases increase travel times. Figure 4a shows a reaction network analogue of the Braess’ network topology. Of course, our chemical reaction network is a little different from a road network since there is no congestion nor competition between molecules and pathways are chosen randomly with certain probabilities instead of routing decisions being based on the number of cars on the routes. Nevertheless, the example considered here is interesting because adding a reaction to cross-link two pathways might intuitively be interpreted as a detour and be expected to increase the time to the final destination, while this is sometimes not the case in this example.

The chemical reaction system is depicted in Fig. 4a, which involves four species ${\mathscr {S}}=\{\mathrm {S_1}, \mathrm {S_2}, \mathrm {S_3}, \mathrm {S_4}\}$. The state of the Markov chain is $(n_{\mathrm {S_1}}, n_{\mathrm {S_2}}, n_{\mathrm {S_3}}, n_{\mathrm {S_4}})$ where $n_\mathrm {S_i}$ is the number of copies of $\mathrm {S_i}$ for $i=1,2,3,4$. The potential transitions of the Markov chain are in five possible directions, $v_j$, $j=1,...,5$, where $v_1=(-1,1,0,0)^T$, $v_2=(0,-1,0,1)^T$, $v_3=(-1,0,1,0)^T$, $v_4=(0,0,-1,1)^T$ and $v_5=(0,-1,1,0)^T$ (see SI-Section S.2.2 for the derivation of the $v_j$, $j=1,...,5$). Fixing an integer $\mathrm {S_{tot}}>0$, the associated stoichiometric compatibility class is $z+{\mathcal {L}}$ with $z = (\mathrm {S_{tot}},0,0,0)$ and ${\mathcal {L}}:= {{\,\textrm{span}\,}}\{v_1,v_2,v_3,v_4,v_5\}$. The set $z+{\mathcal {L}}$ is a three-dimensional affine subspace of four-dimensional space. We choose the state space of our Markov chain to be ${\mathcal {X}}= (z+{\mathcal {L}}) \cap \mathbb {Z}_+^4 = \{(x_1,x_2,x_3,x_4) \in \mathbb {Z}_+^4 | x_1+x_2+x_3+x_4=\mathrm {S_{tot}}\}$. The constraint introduced in the last expression for ${\mathcal {X}}$ follows from the conservation law in this chemical reaction system, that is $n_{\mathrm {S_1}}+n_{\mathrm {S_2}}+n_{\mathrm {S_3}}+n_{\mathrm {S_4}}=\mathrm {S_{tot}}$. Given a generic state $x=(x_1,x_2,x_3,x_4)$, following mass-action kinetics, the infinitesimal transition rates are

22 $$\begin{aligned} \Upsilon _1(x)= \kappa _1 x_1,\;\;\Upsilon _2(x)= \kappa _2 x_2,\;\;\Upsilon _3(x)= \kappa _3 x_1,\;\;\Upsilon _4(x)= \kappa _4 x_3,\;\;\Upsilon _5(x)= \kappa _5 x_2.\nonumber \\ \end{aligned}$$

For the purpose of illustration, Fig. 4b shows the three-dimensional projection of the Markov chain graph for one stoichiometric compatibility class.

A natural question is how the time $T_{(0,0,0,\mathrm {S_{tot}})}$ to reach the state $(0,0,0,\mathrm {S_{tot}})$ from $(\mathrm {S_{tot}},0,0,0)$ depends on the rate constants $\kappa _1$,$\kappa _2$,$\kappa _3$,$\kappa _4$ and $\kappa _5$. For this, we use Theorem 3.4. Let

22 $$\begin{aligned} A= \begin{bmatrix} -1 &{} 0 &{} 0 &{} 0\\ 0 &{} -1 &{} -1 &{} 0 \end{bmatrix}. \end{aligned}$$

The matrix *A* here defines a preorder that is not a partial order of ${\mathcal {X}}$. For $x \in {\mathcal {X}}$, consider infinitesimal transition rates $\breve{\Upsilon }_1(x),\breve{\Upsilon }_2(x), \breve{\Upsilon }_3(x), \breve{\Upsilon }_4(x)$ and $\breve{\Upsilon }_5(x)$ defined as for $\Upsilon _1(x),\Upsilon _2(x),\Upsilon _3(x),\Upsilon _4(x)$ and $\Upsilon _5(x)$, but with $\breve{\kappa }_i$ in place of $\kappa _i$ where $\breve{\kappa }_i=\kappa _i$, for $i = 1,2,3,4$, and $\breve{\kappa }_5 \ne \kappa _5$. Suppose that $\kappa _2 = \kappa _4$. Now, let us verify that the assumptions of Theorem 3.3 hold. Condition (*i*) holds since $Av_1=(1,-1)^T$, $Av_2=(0,1)^T$, $Av_3=(1,-1)^T$, $Av_4=(0,1)^T$ and $Av_5=(0,0)^T$. Condition (*ii*) of Theorem 3.3 also holds, as shown in the paragraph below.

**Verification of condition** (*ii*) **of Theorem**3.3. Let $x \in {\mathcal {X}}$, and first consider $x\in {\mathcal {X}}$ and $y\in \partial _1(K_A+x) \cap {\mathcal {X}}$, where $\partial _1(K_A+x) \cap {\mathcal {X}}= \{ w \in {\mathcal {X}}\,|\, x_1 = w_1, x_2 + x_3 \ge w_2 + w_3, x_4 \le w_4\}$. Given that $Av_2=Av_4, Av_1=Av_3$, and $\langle A_{1\bullet },v_1\rangle =\langle A_{1\bullet },v_3\rangle =1$, we need to check that $\Upsilon _1(x) + \Upsilon _3(x) \le \breve{\Upsilon }_1(y) + \breve{\Upsilon }_3(y)$. Since $y \in \partial _1(K_A+x) \cap {\mathcal {X}}$, then $\Upsilon _1(x) = \kappa _1 x_1 =\kappa _1 y_1 = \breve{\kappa }_1 y_1 = \breve{\Upsilon }_1(y)$ and $\Upsilon _3(x) = \kappa _3 x_1 =\kappa _3 y_1 = \breve{\kappa }_3 y_1 = \breve{\Upsilon }_3(y)$, and so the desired inequality holds with equality. Secondly, consider $y \in \partial _2(K_A+x) \cap {\mathcal {X}}= \{ w \in {\mathcal {X}}\,|\, x_1 \ge w_1, x_2 + x_3 = w_2 + w_3, x_4\le w_4\}$. Given that $Av_2=Av_4, Av_1=Av_3$, and $\langle A_{1\bullet },v_1\rangle =\langle A_{1\bullet },v_3\rangle =-1$ and $\langle A_{1\bullet },v_2\rangle =\langle A_{1\bullet },v_4\rangle =1$, we need to check that $\Upsilon _2(x) + \Upsilon _4(x) \le \breve{\Upsilon }_2(y) + \breve{\Upsilon }_4(y)$ and $\Upsilon _1(x) +\Upsilon _3(x) \ge \breve{\Upsilon }_1(y) + \breve{\Upsilon }_3(y)$. For $x\in {\mathcal {X}}$ and $y\in \partial _2(K_A+x) \cap {\mathcal {X}}$, we have that $\Upsilon _2(x) + \Upsilon _4(x) = \kappa _2 x_2 + \kappa _4 x_3 = \kappa _2 (x_2 + x_3) \le \kappa _2 (y_2 + y_3) = \breve{\kappa }_2 (y_2 + y_3) = \breve{\Upsilon }_2(y) + \breve{\Upsilon }_4(y)$ and $\Upsilon _1(x) = \kappa _1 x_1 \ge \kappa _1 y_1 = \breve{\kappa }_1 y_1 = \breve{\Upsilon }_1(y)$, $\Upsilon _3(x) = \kappa _3 x_1 \ge \kappa _3 y_1 = \breve{\kappa }_3 y_1 = \breve{\Upsilon }_3(y)$.

Thus, all hypotheses of Theorem 3.3 hold, and so for every $x^{\circ },\breve{x}^\circ \in {\mathcal {X}}$ where $x^{\circ }\preccurlyeq _A \breve{x}^\circ$ there there exists a probability space $(\Omega ,{\mathcal {F}},\mathbb {P})$ with two Markov chains $X = \{X(t), \, t \ge 0\}$ and $\breve{X}=\{\breve{X}(t), \, t \ge 0\}$ associated with $\Upsilon$ and $\breve{\Upsilon }$, respectively, such that $X(0)=x^{\circ }$, $\breve{X}(0)=\breve{x}^\circ$ and $\mathbb {P}\left[ X(t) \preccurlyeq _A \breve{X}(t) \text { for every } t \ge 0 \right] =1$. Let $\Gamma = \{(0,0,0,\mathrm {S_{tot}})\}$. This is an increasing set in ${\mathcal {X}}$ with respect to the relation $\preccurlyeq _A$. Let $T_{(0,0,0,\mathrm {S_{tot}})}$, respectively $\breve{T}_{(0,0,0,\mathrm {S_{tot}})}$ be the first time that the Markov chain *X*, respectively $\breve{X}$, reaches the set $\Gamma$. Then, by Theorem 3.4, if $X(0)=\breve{X}(0)=(\mathrm {S_{tot}},0,0,0)$, we have that $\breve{T}_{(0,0,0,\mathrm {S_{tot}})} \preccurlyeq _{st} T_{(0,0,0,\mathrm {S_{tot}})}$. By interchanging $\breve{\Upsilon }_5$ and $\kappa _5$, we can conclude that $\breve{T}_{(0,0,0,\mathrm {S_{tot}})}$ and $T_{(0,0,0,\mathrm {S_{tot}})}$ are stochastically equivalent (equal in distribution). It follows that the mean first passage time from $(\mathrm {S_{tot}},0,0,0)$ to $(0,0,0,\mathrm {S_{tot}})$ is insensitive to $\kappa _5$ when $\kappa _2 = \kappa _4$. This is naively counter-intuitive: since the fifth reaction re-routes some samples to another state where the last reaction has the same rate constant as the final reaction without re-routing, it should take a longer expected time since re-routing also takes some time. However, in reality, the presence of the fifth reaction also fastens the rate to transition from $\mathrm {S_2}$, and this balances the time of re-routing. Most importantly, our theorem is able to capture this result without explicitly calculating the mean first passage time and allows us to reach the conclusion easily. We expect that in more complex situations, our method will be a valuable tool to establish monotonicity and insensitivity results.

Given that the Markov chain has one absorbing state per stoichiometric compatibility class, the stationary distribution for a given stoichiometric compatibility class is trivial, and hence so too are its monotonicity properties.

Theorem S.2 allows us to conclude further interesting properties for this network. Using two other *A* matrices (see SI - Section S.3.2), we can conclude that adding reaction ${\textcircled {\small 5}}$ (changing from $\kappa _5 = 0$ to $\kappa _5 > 0$) causes the mean first passage time from $(\mathrm {S_{tot}},0,0,0)$ to $(0,0,0,\mathrm {S_{tot}})$ to increase if $\kappa _2 > \kappa _4$ or to decrease if $\kappa _2 < \kappa _4$. More explicitly, this shows that there can be opposing effects on the mean first passage time with different choices of $\kappa _2$ and $\kappa _4$ when reaction ${\textcircled {\small 5}}$ is added.

### Example 4.4

**Epigenetic regulation by chromation modifications**

**Fig. 5:**
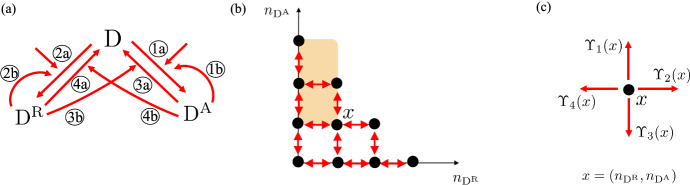
**Histone modification circuit and corresponding Markov chain.** **a** Original chemical reaction system. The numbers on the arrows correspond to the associated reactions. **b** Markov chain graph associated with the reduced chemical reaction system. Here, we consider $\text {D}_{\text {tot}}=3$, we use black dots to represent the states and red double-ended arrows to represent transitions in both directions. We use orange to highlight the region $(K_A +x) \cap {\mathcal {X}}$, with *A* defined in (37). **c** Direction of the possible transitions of the Markov chains, whose rates are given in equation (36)

Epigenetic regulation is the modification of the DNA structure, due to chromatin modifications, that determines if a gene is active or repressed. There are several chromatin modifications that can affect the DNA structure. Here, we will focus only on histone modifications. More precisely, we consider a ubiquitous model for a histone modification circuit (see Dodd et al. (2007) and Bruno et al. (2022)). The species considered are nucleosomes that are unmodified (D), modified with repressive modifications ($\mathrm {D^R}$), and modified with activating modifications ($\mathrm {D^A}$), and, in terms of molecular interactions, each histone modification autocatalyzes itself and promotes the erasure of the other one. The chemical reaction system considered is depicted in Fig. 5a. The amount of each species is represented by $n_{\textrm{D}}$, $n_{\mathrm {D^R}}$ and $n_{\mathrm {D^A}}$, respectively, and their sum is conserved, that is $n_{\textrm{D}}+n_{\mathrm {D^R}}+n_{\mathrm {D^A}}=\text {D}_{\text {tot}}$, with $\text {D}_{\text {tot}}$ representing the total number of nucleosomes within the gene.

By fixing an integer $\text {D}_{\text {tot}}>0$, we fix one stoichiometric compatibility class. The projected process $(X_1,X_2)(\cdot ) = (n_{\mathrm {D^R}},n_{\mathrm {D^A}})$ is still a continuous-time Markov chain, and in this example we choose to apply our theory to this reduced system. This is the same as studying the reduced chemical reaction system defined as follows:

23 $$\begin{aligned} \begin{aligned}&{\textcircled {\small 1}}\;{\emptyset \rightarrow \textrm{D}^{\textrm{A}} },\;\;{\textcircled {\small 2}}\;{\emptyset \rightarrow \textrm{D}^{\textrm{R}} },\;\;{\textcircled {\small 3}}\;{\textrm{D}^{\textrm{A}} \rightarrow \emptyset },\;\;{\textcircled {\small 4}}\;{\textrm{D}^{\textrm{R}} \rightarrow \emptyset }, \end{aligned} \end{aligned}$$

with two species ${\mathscr {S}}=\{\mathrm {D^R}, \mathrm {D^A}\}$ and four reactions ${\mathscr {R}}= \{(v^-_{1},v^+_{1}), (v^-_{2},v^+_{2}),$ $(v^-_{3},v^+_{3}), (v^-_{4},v^+_{4})\}$, where $v^-_{1} =v^-_{2} =v^+_{3} =v^+_{4}=(0,0)^T$, $v^+_{2}= v^-_{4}= (1,0)^T$, $v^+_{1}= v^-_{3}= (0,1)^T$, and with associated propensity functions of non-mass-action type defined as follows:

24 $$\begin{aligned}&\Lambda _{(v^-_{1},v^+_{1})}(x)= (\text {D}_{\text {tot}}-(x_1+x_2))\left( \kappa _{1a} + \kappa _{1b}x_2\right) ,\nonumber \\&\Lambda _{(v^-_{2},v^+_{2})}(x)= (\text {D}_{\text {tot}}-(x_1+x_2))\left( \kappa _{2a} + \kappa _{2b} x_1\right) ,\nonumber \\&\Lambda _{(v^-_{3},v^+_{3})}(x)= x_2\left( \kappa _{3a} + x_1\kappa _{3b}\right) ,\;\;\Lambda _{(v^-_{4},v^+_{4})}(x) = x_1\mu \left( c\kappa _{3a} + x_2 \kappa _{3b}\right) , \end{aligned}$$

in which $\kappa _{1a}$, $\kappa _{1b}$, $\kappa _{3a}$, $\kappa _{3b}$, $\kappa _{2a}$, $\kappa _{2b}$, $\kappa _{4a} = \mu c\kappa _{3a}$, $\kappa _{4b} = \mu \kappa _{3b}$ are the rate constants that go with each of the reactions shown in Fig. 5a, respectively.

The state space for the Markov chain is ${\mathcal {X}}= \{(x_1,x_2) \in \mathbb {Z}_+^2 \,|\, x_1 + x_2 \le \text {D}_{\text {tot}}\}$. Given a generic state $x=(x_1,x_2)\in {\mathcal {X}}$, the potential transitions of the Markov chain are in four possible directions $v_j=v^+_j-v^-_j$, $j=1, 2, 3, 4$, that can be written as $v_{1}= (0,1)^T, v_2=(1,0)^T, v_3=(0,-1)^T$ and $v_4=(-1,0)^T$, with associated infinitesimal transition rates

24 $$\begin{aligned} \begin{aligned}&\Upsilon _{1}(x)= \Lambda _{(v^-_{1},v^+_{1})}(x),\;\;\Upsilon _{2}(x)= \Lambda _{(v^-_{2},v^+_{2})}(x),\;\;\\&\Upsilon _{3}(x)= \Lambda _{(v^-_{3},v^+_{3})}(x),\;\;\Upsilon _{4}(x)= \Lambda _{(v^-_{4},v^+_{4})}(x). \end{aligned} \end{aligned}$$

We are interested in determining how the asymmetry of the system, represented by the parameter $\mu$ affects the stochastic behavior of the system. In particular, we will focus on studying the stationary distribution and the *time to memory loss* of the active and repressed state, defined as the mean first passage time to reach the fully repressed state ($r=(n_{\mathrm {D^R}},n_{\mathrm {D^A}})=(\text {D}_{\text {tot}},0)$), starting from the fully active state ($a=(n_{\mathrm {D^R}},n_{\mathrm {D^A}})=(0,\text {D}_{\text {tot}})$), and vice versa (i.e., $h_{a,r}= \mathbb {E}_{a}[T_{r}]$ and $h_{r,a}= \mathbb {E}_{r}[T_{a}]$). To this end, we first verify that we can apply Theorem 3.2.

Let

25 $$\begin{aligned} A= \begin{bmatrix} -1 &{} 0\\ 0 &{} 1 \end{bmatrix}. \end{aligned}$$

For $x\in {\mathcal {X}}$, $K_A +x = \{ w \in \mathbb {R}^2 \,|\, x \preccurlyeq _A w \}$ and $(K_A +x)\cap {\mathcal {X}}= \{ w \in {\mathcal {X}}\,|\, x \preccurlyeq _A w \}$. See Fig. 5b for an example of ${\mathcal {X}}$ and $(K_A +x) \cap {\mathcal {X}}$ for $\text {D}_{\text {tot}}=3$. We introduce infinitesimal transition rates $\breve{\Upsilon }_{1}(x),\breve{\Upsilon }_{2}(x), \breve{\Upsilon }_{3}(x)$ and $\breve{\Upsilon }_{4}(x)$ defined as for $\Upsilon _{1}(x),\Upsilon _{2}(x),\Upsilon _{3}(x)$ and $\Upsilon _{4}(x)$, with all the parameters having the same values except that $\mu$ is replaced by $\breve{\mu }$, where $\breve{\mu }\ge \mu$. Since $Av_1=(0,1)^T$, $Av_2=(-1,0)^T$, $Av_3=(0,-1)^T$ and $Av_4=(1,0)^T$, we have that condition (*i*) of Theorem 3.2 holds. Condition (*ii*) also holds, as shown in the paragraph below.

**Verification of condition** (*ii*) **of Theorem**3.2. Consider $x\in {\mathcal {X}}$ and $y \in \partial _1(K_A+x) \cap {\mathcal {X}}$, where $\partial _1(K_A+x) \cap {\mathcal {X}}=\{ w \in {\mathcal {X}}\,|\, x_1=w_1, x_2\le w_2\}$. Since $\langle A_{1\bullet },v_{4}\rangle =1$ and $\langle A_{1\bullet },v_{2}\rangle =-1$, we must check that $\Upsilon _{2}(x)\ge \breve{\Upsilon }_{2}(y)$ and $\Upsilon _{4}(x)\le \breve{\Upsilon }_{4}(y)$. Since $y \in \partial _1(K_A+x) \cap {\mathcal {X}}$ implies $x_1=y_1$ and $x_2\le y_2$, we have $\Upsilon _{2}(x) = (\text {D}_{\text {tot}}-(x_1+x_2))\left( \kappa _{2a} + \kappa _{2b} x_1\right) \ge (\text {D}_{\text {tot}}-(y_1+y_2))\left( \kappa _{2a} + \kappa _{2b} y_1\right) = \breve{\Upsilon }_{2}(y)$ and $\Upsilon _{4}(x) =x_1\mu \left( c\kappa _{3a} + x_2 \kappa _{3b}\right) \le y_1\mu \left( c\kappa _{3a} + y_2 \kappa _{3b}\right) \le y_1\breve{\mu }\left( c\kappa _{3a} + y_2 \kappa _{3b}\right) = \breve{\Upsilon }_{4}(y)$, and so both inequalities hold. Similarly, for $x\in {\mathcal {X}}$ and $y \in \partial _2(K_A+x) \cap {\mathcal {X}}=\{ w \in {\mathcal {X}}\,|\, x_1\ge w_1, x_2=w_2 \}$, since $\langle A_{2\bullet },v_{1}\rangle =1$ and $\langle A_{2\bullet },v_{3}\rangle =-1$, we need to check that $\Upsilon _{1}(x)\le \breve{\Upsilon }_{1}(y)$ and $\Upsilon _{3}(x)\ge \breve{\Upsilon }_{3}(y)$. Indeed, $\Upsilon _{1}(x) = (\text {D}_{\text {tot}}-(x_1+x_2))\left( \kappa _{1a} + \kappa _{1b}x_2\right) \le (\text {D}_{\text {tot}}-(y_1+y_2))\left( \kappa _{1a} + \kappa _{1b} y_2\right) = \breve{\Upsilon }_{1}(y)$ and $\Upsilon _{3}(x) = x_2\left( \kappa _{3a} + x_1\kappa _{3b}\right) \ge y_2\left( \kappa _{3a} + y_1\kappa _{3b}\right) = \breve{\Upsilon }_{3}(y)$.

Since all of the hypotheses of Theorem 3.2 hold, for each pair $x^{\circ },\breve{x}^\circ \in {\mathcal {X}}$ satisfying $x^{\circ }\preccurlyeq _A \breve{x}^\circ$, there exists a probability space $(\Omega ,{\mathcal {F}},\mathbb {P})$ with two Markov chains $X = \{X(t), \, t \ge 0\}$ and $\breve{X}=\{\breve{X}(t), \, t \ge 0\}$ associated with $\Upsilon$ and $\breve{\Upsilon }$, respectively, such that $X(0)=x^{\circ }$, $\breve{X}(0)=\breve{x}^\circ$ and $\mathbb {P}\left[ X(t) \preccurlyeq _A \breve{X}(t) \text { for every } t \ge 0 \right] =1$.

We can also apply Theorem 3.5. The Markov chains *X* and $\breve{X}$ are irreducible and, having only finitely many states, are positive recurrent. Based on the order $\preccurlyeq _A$ we introduced, the fully active state $a=(0,\text {D}_{\text {tot}})$ is maximal in ${\mathcal {X}}$ and the fully repressed state $r=(\text {D}_{\text {tot}},0)$ is minimal in ${\mathcal {X}}$. Then, by Theorem 3.5, we can conclude that $\pi _a \le \breve{\pi }_a$ and $\pi _r \ge \breve{\pi }_r$. This implies that increasing $\mu$ increases the probability of the system in steady-state to be in the active state *a* to the detriment of the repressed state *r* (and vice versa for decreasing $\mu$). We can also apply Theorem 3.4. Since $\{a\}$ is increasing and $\{r\}$ is decreasing, then by Theorem 3.4, $\breve{h}_{r,a}=\mathbb {E}_{r}[\breve{T}_{a}]\le \mathbb {E}_{r}[T_{a}]=h_{r,a}$ and $h_{a,r}=\mathbb {E}_{a}[T_{r}]\le \mathbb {E}_{a}[\breve{T}_{r}]=\breve{h}_{a,r}$. Since the only difference between the two systems was that $\mu \le \breve{\mu }$, these results imply that the time to memory loss of the active state increases for higher values of $\mu$, while the time to memory loss of the repressed state decreases for higher values of $\mu$.

### Example 4.5

**Epigenetic regulation by chromatin modifications with positive TF-enabled autoregulation**

Now, we consider the histone modification circuit considered in the previous example with an additional positive autoregulation loop. For this, we assume that a protein expressed by the gene of interest recruits writers for the activating histone modifications. Consequently, we introduce the gene product P as an additional species for our system and add the following reactions to the ones shown in Fig. 5a:

25 $$\begin{aligned} \begin{aligned}&{\textcircled {\small 5a}}\;{\textrm{D}^{\textrm{A}} \rightarrow \textrm{D}^{\textrm{A}} + \textrm{P} },\;\;{\textcircled {\small 6a}}\; {\textrm{P} \rightarrow \emptyset }. \end{aligned} \end{aligned}$$

**Fig. 6:**
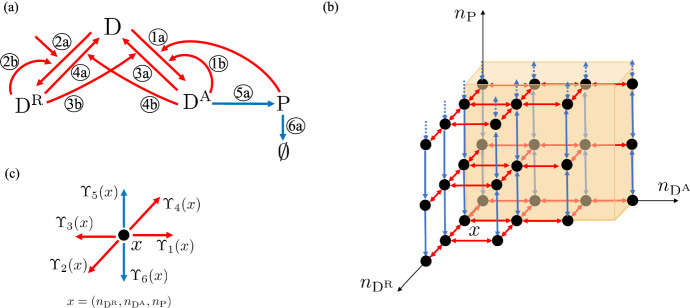
**Histone modification circuit with positive TF-enabled autoregulation and corresponding Markov chain.** **a** Original chemical reaction system. The numbers on the arrows correspond to the associated reactions. **b** Markov chain graph. Here, we consider $\text {D}_{\text {tot}}=3$, we use black dots to represent the states and red double-ended arrows to represent transitions in both directions associated with the reactions represented by the red arrows in **a**. Similarly we use blue double-ended (single-ended) arrows to represent transitions in both directions (in one direction) associated with the reactions represented by the blue arrows in **a**. We use blue dotted lines to show that, in the vertical direction, the Markov chain has countably infinitely many states, connected by transitions in both directions. Finally, we use orange to highlight the region $K_A+x$ intersected with the state space ${\mathcal {X}}$, with *A* defined in (41). **c** Direction of the possible transitions of the Markov chain starting from a state *x*, whose rates are defined in equation (40)

Furthermore, given the P-enabled autoregulation loop (Fig. 6a), let us consider the rate constant that goes with ${\textcircled {\small 1a}}$ in Fig. 5a as $\kappa _{1a}=\kappa _{1a}^0+\kappa _{1a}^1g(n_{\textrm{P}})$, with $\kappa _{1a}^0$ and $\kappa _{1a}^1$ representing the rate constants that go with the $\mathrm {D^A}$ basal *de-novo* establishment process and with the $\mathrm {D^A}$ *de-novo* establishment process enhanced by $n_{\textrm{P}}$, respectively, and $g(n_{\textrm{P}})$ representing a nonnegative, bounded, monotonically increasing function of $n_{\textrm{P}}$ (see Bruno et al. (2022), Sect. 3.4).

Here, we are interested in determining how the reaction rate constant $\kappa _{5a}$ affects the reactivation time of the gene. As before, we have the conservation law $n_{\textrm{D}}+n_{\mathrm {D^R}}+n_{\mathrm {D^A}}=\text {D}_{\text {tot}}$, with $\text {D}_{\text {tot}}$ representing the total number of nucleosomes within the gene, and by fixing $\text {D}_{\text {tot}}>0$, we fix one stoichiometric compatibility class and the projected process $(X_1,X_2,X_3)(\cdot ) = (n_{\mathrm {D^R}},n_{\mathrm {D^A}},n_{\textrm{P}})$ is a continuous-time Markov chain. This is the same as studying the reduced chemical reaction system:

26 $$\begin{aligned} \begin{aligned}&{\textcircled {\small 1}}\;{\emptyset \rightarrow \textrm{D}^{\textrm{A}} },\;\;{\textcircled {\small 2}}\;{\emptyset \rightarrow \textrm{D}^{\textrm{R}} },\;\;{\textcircled {\small 3}}\;{\textrm{D}^{\textrm{A}} \rightarrow \emptyset },\\&{\textcircled {\small 4}}\;{\textrm{D}^{\textrm{R}} \rightarrow \emptyset },\;\;{\textcircled {\small 5}}\;{\textrm{D}^{\textrm{A}} \rightarrow \textrm{D}^{\textrm{A}} + \textrm{P} },\;\;{\textcircled {\small 6}}\;{\textrm{P} \rightarrow \emptyset },\\ \end{aligned} \end{aligned}$$

with set of species ${\mathscr {S}}=\{\mathrm {D^R}, \mathrm {D^A}, \textrm{P}\}$, set of reactions ${\mathscr {R}}= \{(v^-_{1},v^+_{1})$, $(v^-_{2},v^+_{2})$, $(v^-_{3},v^+_{3})$, $(v^-_{4},v^+_{4})$, $(v^-_{5},v^+_{5})$, $(v^-_{6},v^+_{6})\}$, where $v^-_{1} =v^-_{2} =v^+_{3} =v^+_{4}=v^+_{6} =(0,0,0)^T$, $v^+_{2}= v^-_{4}=(1,0,0)^T$, $v^+_{1}= v^-_{3}= v^-_{5}=(0,1,0)^T$, $v^+_{5}=(0,1,1)^T$, $v^-_{6} =(0,0,1)^T$, and with associated propensity functions of non-mass-action type defined as follows:

$$\begin{aligned} \begin{aligned}&\Lambda _{(v^-_{1},v^+_{1})}(x)= (\text {D}_{\text {tot}}-(x_1+x_2))\left( \kappa _{1a}^0+\kappa _{1a}^1g(x_3) + \kappa _{1b}x_2\right) ,\\&\Lambda _{(v^-_{2},v^+_{2})}(x)= (\text {D}_{\text {tot}}-(x_1+x_2))\left( \kappa _{2a} + \kappa _{2b}x_1\right) ,\;\;\Lambda _{(v^-_{3},v^+_{3})}(x)= x_2\left( \kappa _{3a} + x_1 \kappa _{3b}\right) ,\\&\Lambda _{(v^-_{4},v^+_{4})}(x) = x_1\mu \left( c\kappa _{3a} + x_2\kappa _{3b}\right) ,\;\;\Lambda _{(v^-_{5},v^+_{5})}(x) = \kappa _{5a} x_2,\;\;\Lambda _{(v^-_{6},v^+_{6})}(x) = \kappa _{6a} x_3, \end{aligned} \end{aligned}$$

in which $\kappa _{5a}$ and $\kappa _{6a}$ are the rate constants that go with reactions ${\textcircled {\small 5a}}$ and ${\textcircled {\small 6a}}$ in (38), respectively, and all the other rate constants are defined as for (35).

The state space for the Markov chain is ${\mathcal {X}}= \{(x_1,x_2,x_3) \in \mathbb {Z}_+^3 \,|\, x_1 + x_2 \le \text {D}_{\text {tot}}\}$. Given a generic state $x=(x_1,x_2,x_3)$, the transitions of the Markov chain are in six possible directions $v_j=v^+_j-v^-_j$, $j\in \{1,..., 6\}$, that can be written as $v_1=(0,1,0)^T$, $v_2=(1,0,0)^T$, $v_3=(0,-1,0)^T$, $v_4=(-1,0,0)^T$, $v_5=(0,0,1)^T$, $v_6=(0,0,-1)^T$, with associated infinitesimal transition rates:

27 $$\begin{aligned} \begin{aligned}&\Upsilon _{1}(x)= \Lambda _{(v^-_{1},v^+_{1})}(x),\;\;\Upsilon _{2}(x)= \Lambda _{(v^-_{2},v^+_{2})}(x),\;\;\Upsilon _{3}(x)= \Lambda _{(v^-_{3},v^+_{3})}(x),\\&\Upsilon _{4}(x)= \Lambda _{(v^-_{4},v^+_{4})}(x),\;\;\Upsilon _{5}(x)= \Lambda _{(v^-_{5},v^+_{5})}(x),\;\;\Upsilon _{6}(x)= \Lambda _{(v^-_{6},v^+_{6})}(x). \end{aligned} \end{aligned}$$

As mentioned before, we are interested in determining how the protein production rate $\kappa _{5a}$ affects the reactivation time of the gene, defined as $h_{r,\Theta }= \mathbb {E}_{r}[T_{\Theta }]$, where $r=(\text {D}_{\text {tot}},0,0)$ and $\Theta =\{w\in {\mathcal {X}}| w=(0,\text {D}_{\text {tot}},i), i\in \mathbb {Z}_{+}\}$ corresponds to the set of states characterized by the fully active state $n_{\mathrm {D^A}}=\text {D}_{\text {tot}}$. We first check that the assumptions of Theorem 3.2 hold. Let

28 $$\begin{aligned} A= \begin{bmatrix} -1 &{} 0 &{} 0\\ 0 &{} 1 &{} 0\\ 0 &{} 0 &{} 1 \end{bmatrix}. \end{aligned}$$

For $x\in {\mathcal {X}}$, $x \preccurlyeq _A y$ and the set $K_A +x = \{ y \in \mathbb {R}^3 \,|\, x \preccurlyeq _A y \}$. For our example, the region $(K_A +x) \cap {\mathcal {X}}$ is depicted in orange in Fig. 6b. We introduce infinitesimal transition rates $\breve{\Upsilon }_{1}(x),\breve{\Upsilon }_{2}(x), \breve{\Upsilon }_{3}(x), \breve{\Upsilon }_{4}(x), \breve{\Upsilon }_{5}(x)$ and $\breve{\Upsilon }_{6}(x)$ defined as for $\Upsilon _{1}(x),\Upsilon _{2}(x),\Upsilon _{3}(x),\Upsilon _{4}(x),\Upsilon _{5}(x)$ and $\Upsilon _{6}(x)$, with all the parameters having the same values except that $\kappa _{5a}$ is replaced by $\breve{\kappa }_{5a}> \kappa _{5a}$. Condition (*i*) of Theorem 3.2 holds since $Av_{1}=(0,1,0)^T$, $Av_{2}=(-1,0,0)^T$, $Av_{3}=(0,-1,0)^T$, $Av_4=(1,0,0)^T$, $Av_5=(0,0,1)^T$, $Av_6=(0,0,-1)^T$. Condition (*ii*) also holds, as shown in the paragraph below.

**Verification of condition** (*ii*) **of Theorem **3.2. First consider $x\in {\mathcal {X}}$ and $y \in \partial _1(K_A+x) \cap {\mathcal {X}}=\{ w \in {\mathcal {X}}\,|\, x_1=w_1, x_2\le w_2, x_3\le w_3\}$. Since $\langle A_{1\bullet },v_{4}\rangle =1$ and $\langle A_{1\bullet },v_{2}\rangle =-1$, we need to check that $\Upsilon _{4}(x)\le \breve{\Upsilon }_{4}(y)$ and $\Upsilon _{2}(x)\ge \breve{\Upsilon }_{2}(y)$. Since $x_1=y_1, x_2\le y_2, x_3\le y_3$, we have that $\Upsilon _{4}(x)=x_1\mu \left( c\kappa _{3a} + x_2\kappa _{3b}\right) \le y_1\mu \left( c\kappa _{3a} + y_2\kappa _{3b}\right) = \breve{\Upsilon }_{4}(y)$ and $\Upsilon _{2}(x)= (\text {D}_{\text {tot}}-(x_1+x_2))\left( \kappa _{2a} + \kappa _{2b}x_1\right) \ge (\text {D}_{\text {tot}}-(y_1+y_2))\left( \kappa _{2a} + \kappa _{2b}y_1\right) = \breve{\Upsilon }_{2}(y)$. Secondly, consider $x\in {\mathcal {X}}$ and $y \in \partial _2(K_A+x) \cap {\mathcal {X}}=\{ w \in {\mathcal {X}}\,|\, x_1\ge w_1, x_2=w_2, x_3\le w_3\}$. Since $\langle A_{2\bullet },v_{1}\rangle =1$ and $\langle A_{2\bullet },v_{3}\rangle =-1$, we need to check that $\Upsilon _{1}(x)\le \breve{\Upsilon }_{1}(y)$ and $\Upsilon _{3}(x)\ge \breve{\Upsilon }_{3}(y)$. Since $x_1\ge y_1, x_2=y_2, x_3\le y_3$, we have $\Upsilon _{1}(x)= (\text {D}_{\text {tot}}-(x_1+x_2))\left( \kappa _{1a}^0+\kappa _{1a}^1\,g(x_3) + \kappa _{1b}x_2\right) \le (\text {D}_{\text {tot}}-(y_1+y_2))\left( \kappa _{1a}^0+\kappa _{1a}^1\,g(y_3) + \kappa _{1b} y_2\right) = \breve{\Upsilon }_{1}(y)$ and $\Upsilon _{3}(x)=x_2\left( \kappa _{3a} + x_1 \kappa _{3b}\right) \ge y_2\left( \kappa _{3a} + y_1 \kappa _{3b}\right) = \breve{\Upsilon }_{3}(y)$. Finally, consider $x\in {\mathcal {X}}$ and $y \in \partial _3(K_A+x) \cap {\mathcal {X}}=\{ w \in {\mathcal {X}}\,|\, x_1 \ge w_1, x_2\le w_2, x_3=w_3\}$. Since $\langle A_{3\bullet },v_{5}\rangle =1$ and $\langle A_{3\bullet },v_{6}\rangle =-1$, we must check that $\Upsilon _{5}(x)\le \breve{\Upsilon }_{5}(y)$ and $\Upsilon _{6}(x)\ge \breve{\Upsilon }_{6}(y)$. Since $x_1 \ge y_1, x_2\le y_2, x_3=y_3$, we obtain $\Upsilon _{5}(x)=\kappa _{5a} x_2 \le \kappa _{5a} y_2 \le \breve{\kappa }_{5a} y_2 = \breve{\Upsilon }_{5}(y)$ and $\Upsilon _{6}(x)=\kappa _{6a} x_3 =\kappa _{6a} y_3 =\breve{\Upsilon }_{6}(y)$.

Since all the hypotheses of Theorem 3.2 hold, for each $x^{\circ },\breve{x}^\circ \in {\mathcal {X}}$ satisfying $x^{\circ }\preccurlyeq _A \breve{x}^\circ$, there exists a probability space $(\Omega ,{\mathcal {F}},\mathbb {P})$ with two Markov chains $X = \{X(t), \, t \ge 0\}$ and $\breve{X}=\{\breve{X}(t), \, t \ge 0\}$ associated with $\Upsilon$ and $\breve{\Upsilon }$, respectively, such that $X(0)=x^{\circ }$, $\breve{X}(0)=\breve{x}^\circ$ and $\mathbb {P}\left[ X(t) \preccurlyeq _A \breve{X}(t) \text { for every } t \ge 0 \right] =1$.

Furthermore, since the hypotheses of Theorem 3.2 hold, we can also apply Theorem 3.4. Specifically, for $r=(\text {D}_{\text {tot}},0,0)$ and $\Theta =\{y\in {\mathcal {X}}| y=(0,\text {D}_{\text {tot}},i), i\in \mathbb {Z}_{+}\}$, since $\Theta$ is an increasing set in ${\mathcal {X}}$ with respect to the relation $\preccurlyeq _A$, then $h_{r,\Theta }\ge \breve{h}_{r,\Theta }$. This implies that, assuming that the only difference between the two systems is in the value of the protein production rate parameter, $\kappa _{5a}$, higher protein production rates reduce the mean reaction time for the gene.

## Proofs of the Main Results

### Proof of Theorem 3.1.

Consider a non-empty set ${\mathcal {X}}\subseteq \mathbb {Z}_+^d$, a collection of distinct vectors $v_1,\ldots ,v_n$ in $\mathbb {Z}^d {\setminus } \{0\}$ and two collections of nonnegative functions on ${\mathcal {X}}$, $\Upsilon =(\Upsilon _1, \dots ,\Upsilon _n)$ and $\breve{\Upsilon }= (\breve{\Upsilon }_1, \dots ,\breve{\Upsilon }_n)$, such that (8) holds. Let $Q=(Q_{x,y})_{x,y \in {\mathcal {X}}}$ and $\breve{Q}=(\breve{Q}_{x,y})_{x,y \in {\mathcal {X}}}$ denote the infinitesimal generators for the continuous-time Markov chains associated with $\Upsilon$ and $\breve{\Upsilon }$, respectively. In the following, let $A \in \mathbb {R}^{m \times d}$ be a matrix with nonzero rows and consider the relation $\preccurlyeq _A$ as defined in Definition 3.1.

For the proof of Theorem 3.1, we first assume that

28 $$\begin{aligned} \sup _{x \in {\mathcal {X}}} \Upsilon _j(x)< \infty \quad \text { and } \quad \sup _{x \in {\mathcal {X}}} \breve{\Upsilon }_j(x) < \infty \qquad \text { for every } 1 \le j \le n. \end{aligned}$$

This restriction will be relaxed later. Then, we define a constant $\lambda > 0$ and a pair of functions $\Phi _{\lambda }$ and $\breve{\Phi }_{\lambda }$, which will be key to our construction of the coupled processes *X* and $\breve{X}$. Let $\lambda > 0$ such that:

29 $$\begin{aligned} \lambda > n\max \left\{ \sup _{x \in {\mathcal {X}}} \sum _{j=1}^n\Upsilon _j(x), \sup _{x \in {\mathcal {X}}} \sum _{j=1}^n\breve{\Upsilon }_{j}(x) \right\} . \end{aligned}$$

Note that both $\frac{\Upsilon _j(x)}{\lambda }$ and $\frac{\breve{\Upsilon }_j(x)}{\lambda }$ are less than $\frac{1}{n}$ for every $x \in {\mathcal {X}}$ and $1 \le j \le n$. For $x \in {\mathcal {X}}$, consider the sets

29 $$\begin{aligned} I_j(x):= \left[ \frac{j-1}{n},\frac{j-1}{n} + \frac{\Upsilon _j(x)}{\lambda }\right) , \qquad 1 \le j \le n. \end{aligned}$$

If $\Upsilon _j(x) = 0$, then $I_j(x)$ is the empty set. On the other hand, if $\Upsilon _j(x) >0$, then $I_j(x)$ is an interval that is a strict subset of $[\frac{j-1}{n},\frac{j}{n})$. Define the function $\Phi _{\lambda }(\cdot ,\cdot ): {\mathcal {X}}\times [0,1] \longrightarrow {\mathcal {X}}$ by

30 $$\begin{aligned} \Phi _{\lambda }(x,u):= x + \sum _{j=1}^n v_j\mathbbm {1}_{I_j(x)}(u), \qquad x \in {\mathcal {X}}, \; u \in [0,1]. \end{aligned}$$

For $x \in {\mathcal {X}}$, the sets $I_1(x),\ldots ,I_n(x)$ are mutually disjoint and so for any $u \in [0,1]$ either $\Phi _{\lambda }(x,u) =x$ or $\Phi _{\lambda }(x,u) =x + v_j$ for some $1 \le j \le n$. In the second case, this will happen if and only if $u \in I_j(x)$ for the corresponding index *j*. The latter condition implies that $I_j(x) \ne \emptyset$, hence by (44), $\Upsilon _{j}(x) > 0$ and by (8), $x + v_{j} \in {\mathcal {X}}$.

This shows that $\Phi _{\lambda }(\cdot ,\cdot )$ is well-defined as an ${\mathcal {X}}$-valued function. We define intervals $\breve{I}_j(x), \, 1 \le j \le n, \, x \in {\mathcal {X}}$ and a function $\breve{\Phi }_{\lambda }:{\mathcal {X}}\times [0,1] \longrightarrow {\mathcal {X}}$ in an analogous manner to that above, where $\breve{\Phi }_{\lambda }$ is defined as in (45), but with the intervals $I_j(x)$ replaced by $\breve{I}_j(x)$, where these are defined as in (44), but with $\Upsilon _j(x)$ replaced by $\breve{\Upsilon }_j(x)$.

#### Lemma 5.1

Suppose that $x,y \in {\mathcal {X}}$ are such that $x \preccurlyeq _A y$ and the following hold:

30 $$\begin{aligned} \breve{\Upsilon }_j(y) \le \Upsilon _j(x), \quad \text {for each } 1 \le j \le n \text { such that } y +v_j \in {\mathcal {X}}\setminus (K_A +x), \end{aligned}$$

and

31 $$\begin{aligned} \breve{\Upsilon }_j(y)\ge \Upsilon _j(x), \quad \text {for each } 1 \le j \le n \text { such that } x +v_j \in {\mathcal {X}}\text { and } y \notin K_A + x+ v_j.\nonumber \\ \end{aligned}$$

Then, for each $u \in [0,1]$,

31 $$\begin{aligned} \Phi _{\lambda }(x,u) \preccurlyeq _A \breve{\Phi }_{\lambda }(y,u). \end{aligned}$$

#### Proof

First, we note that $\Phi _{\lambda }, \breve{\Phi }_{\lambda }$ have the following property: for every $u \in [0,1]$ and $1 \le j \le n$,

32 $$\begin{aligned} \text { if } \; \Phi _{\lambda }(x,u) = x +v_j, \, \text { then } \; \breve{\Phi }_{\lambda }(y,u) \in \{y,y +v_j\}, \end{aligned}$$

since $$I_j(x), \breve{I}_j(y) \subseteq [\frac{j-1}{n},\frac{j}{n})$$. Similarly,

32 $$\begin{aligned} \text { if } \; \breve{\Phi }_{\lambda }(y,u) = y +v_j, \, \text { then } \; \Phi _{\lambda }(x,u) \in \{x,x +v_j\}. \end{aligned}$$

Furthermore, if $\breve{\Upsilon }_j(y) \ge \Upsilon _j(x)$, then

33 $$\begin{aligned} \Phi _{\lambda }(x,u) = x +v_j \, \text { implies that } \; \breve{\Phi }_{\lambda }(y,u) =y +v_j, \end{aligned}$$

since under this condition, $I_j(x) \subseteq \breve{I}_j(y)$. Similarly, if $\breve{\Upsilon }_j(y) \le \Upsilon _j(x)$, then

33 $$\begin{aligned} \breve{\Phi }_{\lambda }(y,u) = y +v_j \, \text { implies that } \; \Phi _{\lambda }(x,u) =x +v_j. \end{aligned}$$

Now, to prove (48), fix $u \in [0,1]$. We consider two cases.

**Case 1:** $\breve{\Phi }_{\lambda }(y,u) = y +v_j$ for some $1 \le j \le n$.

Fix such an index *j*. Then, by (50), either $\Phi _{\lambda }(x,u) = x + v_j$ or $\Phi _{\lambda }(x,u) = x$.

- If $\Phi _{\lambda }(x,u) = x + v_j$, then, by (6), $x + v_j \preccurlyeq _A y +v_j$ and therefore $\Phi _{\lambda }(x,u) \preccurlyeq _A \breve{\Phi }_{\lambda }(y,u)$.
- If $\Phi _{\lambda }(x,u) = x$, then $y + v_j \in K_A +x$. To see this, we note that $y + v_j \in {\mathcal {X}}$ by (8) and since $\breve{\Upsilon }_j(y) > 0$ because $\breve{I}_j(y) \ne \emptyset$. Then, if $y + v_j \notin K_A + x$, by (46), we would have $\breve{\Upsilon }_j(y) \le \Upsilon _j(x)$, which would imply that $\Phi _{\lambda }(x,u) = x +v_j$ by (52). But this contradicts the assumption that $\Phi _{\lambda }(x,u)=x$. Thus, $y + v_j \in K_A + x$ and so $\Phi _{\lambda }(x,u)=x \preccurlyeq _A y+v_j=\breve{\Phi }_{\lambda }(y,u)$.

**Case 2:** $\breve{\Phi }_{\lambda }(y,u) = y$. Again, we consider two subcases.

- If $\Phi _{\lambda }(x,u)=x$, then (48) holds, since $x \preccurlyeq _A y$ by assumption.
- If $\Phi _{\lambda }(x,u) = x +v_j$ for some $1 \le j \le n$, then $y \in K_A + x + v_j$ for the corresponding value of *j*. To see this, fix the value of *j* for which $\Phi _{\lambda }(x,u)=x+v_j$ and notice that $x +v_j \in {\mathcal {X}}$ by (8) and since $\Upsilon _j(x) >0$. If $y \notin K_A +x+v_j$, then by (47) we would have $\Upsilon _j(x) \le \breve{\Upsilon }_j(y)$, which would imply that $\breve{\Phi }_{\lambda }(y,u) = y +v_j$. This contradicts the assumption that $\breve{\Phi }_{\lambda }(y,u)=y$. Thus, we must have $y \in K_A +x +v_j$ and then $\Phi _{\lambda }(x,u)=x+v_j \preccurlyeq _A y=\breve{\Phi }_{\lambda }(y,u)$. $\square$

Now that all these preliminaries have been established under assumption (42), we proceed with the main part of the proof of Theorem 3.1 with this assumption. For this proof, we assume that all of the conditions of Theorem 3.1 hold and in addition that condition (42) holds. The latter ensures that the pair of continuous-time Markov chains with infinitesimal generators *Q* and $\breve{Q}$ are *uniformizable* (see Chapter 2 in Keilson (1979)). With $\lambda > 0$ as in (43), the (possibly infinite) matrices^4^$P_{\lambda }(Q):= \frac{1}{\lambda }Q +I$ and $P_{\lambda }(\breve{Q}):= \frac{1}{\lambda }\breve{Q} +I$ are stochastic,^5^ where $I =(I_{x,y})_{x,y \in {\mathcal {X}}}$ is the identity matrix. Indeed, for $x \in {\mathcal {X}}$, $(P_{\lambda }(Q))_{x,x} = \frac{Q_{x,x}}{\lambda } + 1 = 1 - \frac{|Q_{x,x}|}{\lambda } \in [1-\frac{1}{n},1]$, for $y \ne x$, $(P_{\lambda }(Q))_{x,y} = \frac{Q_{x,y}}{\lambda } \in [0, \frac{1}{n}]$ and $\sum _{y \in {\mathcal {X}}} (P_{\lambda }(Q))_{x,y} = \sum _{y \in {\mathcal {X}}} \frac{1}{\lambda }Q_{x,y} + 1 = 1$.

Now, let $x^{\circ },\breve{x}^\circ \in {\mathcal {X}}$ be such that $x^{\circ }\preccurlyeq _A \breve{x}^\circ$. Consider a probability space $(\Omega ,{\mathcal {F}},\mathbb {P})$ where the following are defined:

(i) A Poisson process $N = \{N(t), \, 0 \le t < \infty \}$ of rate $\lambda > 0$.
(ii) A sequence of independent and identically distributed (i.i.d.) random variables $U =(U_k)_{k \ge 1}$ where each $U_k$ has the uniform distribution on [0, 1].

Additionally, choose *N* to be independent of *U*. We construct two discrete-time processes, $Y=(Y_k)_{k \ge 0}$ and $\breve{Y}=(\breve{Y}_k)_{k \ge 0}$, by defining $Y_0:= x^{\circ }$, $\breve{Y}_0:= \breve{x}^\circ$, and for $k \ge 0$,

34 $$\begin{aligned} Y_{k+1}:= \Phi _{\lambda }(Y_k,U_{k+1}), \qquad \breve{Y}_{k+1}:= \breve{\Phi }_{\lambda }(\breve{Y}_k,U_{k+1}). \end{aligned}$$

Then *Y* and $\breve{Y}$ are discrete-time Markov chains with transition matrices $P_{\lambda }(Q)$ and $P_{\lambda }(\breve{Q})$, respectively. Now, define the processes

34 $$\begin{aligned} X(t):= Y_{N(t)}, \qquad \breve{X}(t):= \breve{Y}_{N(t)}, \qquad t \ge 0. \end{aligned}$$

According to Section 2.1 in Keilson (1979) (see the discussion around Equation 2.1.6), *X* and $\breve{X}$ are continuous-time Markov chains with infinitesimal generators *Q* and $\breve{Q}$, respectively, and with initial conditions $X(0)=x^{\circ }$ and $\breve{X}(0)=\breve{x}^\circ$.

In order to prove (12), it suffices to check that the following holds:

35 $$\begin{aligned} \mathbb {P}[Y_k \preccurlyeq _A \breve{Y}_k] =1, \qquad \text { for every } k \ge 0. \end{aligned}$$

Indeed, if this is true, then $\mathbb {P}[Y_k \preccurlyeq _A \breve{Y}_k \text { for every } k \ge 0] =1$ and therefore $\mathbb {P}[Y_{N(t)} \preccurlyeq _A \breve{Y}_{N(t)} \text { for every }$ $t \ge 0] =1$. We will prove (55) by induction on *k*. We already know that $x^{\circ }\preccurlyeq _A \breve{x}^\circ$ and so (55) holds for $k=0$. Now, assume $\mathbb {P}[Y_k \preccurlyeq _A \breve{Y}_k] =1$ for some $k \ge 0$. Since conditions (10) and (11) hold for every $x,y \in {\mathcal {X}}$ such that $x \preccurlyeq _A y$, by Lemma 5.1 we obtain that on a set of probability one, on which $Y_k \preccurlyeq _A \breve{Y}_{k}$,

36 $$\begin{aligned} Y_{k+1} =\Phi _{\lambda }(Y_k,U_{k+1}) \preccurlyeq _A \breve{\Phi }_{\lambda }(\breve{Y}_k,U_{k+1}) = \breve{Y}_{k+1}, \end{aligned}$$

and so (55) holds with $k+1$ in place of *k*. This completes the induction step, and so Theorem 3.1 is proved whenever (42) holds.

For the case where (42) does not hold, we construct the corresponding continuous-time Markov chains as a limit in distribution of appropriately coupled continuous-time Markov chains with truncated propensity functions for which (42) holds. Many elements for this case are similar to the previous case, although the use of Lemma 5.1 is different. We provide the details below, where we assume that the hypotheses of Theorem 3.1 hold.

We consider truncations of the propensity functions $\Upsilon$ and $\breve{\Upsilon }$. More concretely, for $x^{\circ },\breve{x}^\circ \in {\mathcal {X}}$ such that $x^{\circ }\preccurlyeq _A \breve{x}^\circ$, let $M_0 \ge 1$ be an integer such that $\Vert x^{\circ }\Vert _{\infty },\Vert \breve{x}^\circ \Vert _{\infty } \le M_0$. For every integer $M \ge M_0$, consider the finite set ${\mathcal {X}}_{M}:= \{ x \in {\mathcal {X}}\,|\, \Vert x\Vert _{\infty } \le M\}$, together with the functions $\Upsilon ^M_j,\breve{\Upsilon }^M_j: {\mathcal {X}}\longrightarrow \mathbb {R}_+$ defined by $\Upsilon ^M_j(x):= \Upsilon _j(x)\mathbbm {1}_{{\mathcal {X}}_{M}}(x)$ and $\breve{\Upsilon }^M_j(x):= \breve{\Upsilon }_j(x)\mathbbm {1}_{{\mathcal {X}}_{M}}(x)$ for $1 \le j \le n$ and $x \in {\mathcal {X}}$. We see that for every $M \ge M_0$, (8) holds with the functions $\Upsilon ^M=(\Upsilon ^M_1, \dots ,\Upsilon ^M_n)$ and $\breve{\Upsilon }^M= (\breve{\Upsilon }^M_1, \dots ,\breve{\Upsilon }^M_n)$ in place of $\Upsilon$ and $\breve{\Upsilon }$. Also, since ${\mathcal {X}}_M$ is a finite set, $\sup _{x \in {\mathcal {X}}} \Upsilon ^M_j(x) = \sup _{x \in {\mathcal {X}}_M} \Upsilon _j(x) < \infty$ and $\sup _{x \in {\mathcal {X}}} \breve{\Upsilon }^M_j(x) = \sup _{x \in {\mathcal {X}}_M} \breve{\Upsilon }_j(x) < \infty$ for every $1 \le j \le n$. Furthermore, by (10) and (11), we have that for every pair $x,y \in {\mathcal {X}}_M$ such that $x \preccurlyeq _A y$,

36 $$\begin{aligned} \begin{aligned} \breve{\Upsilon }^M_j(y) \le \Upsilon ^M_j(x), \quad \text {for every } 1 \le j \le n \text { such that } y +v_j \in {\mathcal {X}}\setminus (K_A + x), \text { and } \\ \breve{\Upsilon }^M_j(y)\ge \Upsilon ^M_j(x), \quad \text {for every } 1 \le j \le n \text { such that } x +v_j \in {\mathcal {X}}\text { and } y \notin K_A + x + v_j. \end{aligned} \end{aligned}$$

Let $Q^M$ and $\breve{Q}^M$ denote the infinitesimal generators associated with $\Upsilon ^M$ and $\breve{\Upsilon }^M$, respectively. We define an increasing sequence $\{\lambda _M\}_{M \ge M_0}$ of positive numbers such that $\lambda _{M} \longrightarrow \infty$ as $M \longrightarrow \infty$ and $\lambda _M > n\max \left\{ \sup _{x \in {\mathcal {X}}} \sum _{j=1}^n\Upsilon ^M_j(x), \sup _{x \in {\mathcal {X}}} \sum _{j=1}^n\breve{\Upsilon }^M_{j}(x) \right\}$ for every $M \ge M_0$. Define $\Phi _{\lambda _M}(\cdot ,\cdot ),\breve{\Phi }_{\lambda _M}(\cdot ,\cdot ): {\mathcal {X}}\times [0,1] \longrightarrow {\mathcal {X}}$ as in (45), but with $\Upsilon ^M$ and $\breve{\Upsilon }^M$ in place of $\Upsilon$ and $\breve{\Upsilon }$, respectively. Since (57) holds, applying Lemma 5.1 with $\Upsilon ^M,\breve{\Upsilon }^M,\lambda _M,\Phi _{\lambda _M},\breve{\Phi }_{\lambda _M}$ in place of $\Upsilon ,\breve{\Upsilon },\lambda ,\Phi _{\lambda },\breve{\Phi }_{\lambda }$ yields that

37 $$\begin{aligned} \Phi _{\lambda _M}(x,u) \preccurlyeq _A \breve{\Phi }_{\lambda _M}(y,u) \quad \text { for every } x,y \in {\mathcal {X}}_M \text { such that } x \preccurlyeq _A y \text { and } u \in [0,1].\nonumber \\ \end{aligned}$$

Now, for each $M \ge M_0$ consider a probability space $(\Omega ^M,{\mathcal {F}}^M,\mathbb {P}^M)$ where the following are defined:

(i) A Poisson process $N^{M} = \{N^M(t), \, 0 \le t < \infty \}$ of rate $\lambda _M > 0$.
(ii) An i.i.d. sequence $U^M =(U^M_{k})_{k \ge 1}$ of uniform [0, 1] random variables.

Additionally, choose $N^M$ to be independent of $U^M$. For every $M \ge M_0$, we construct two discrete-time processes, $Y^{M}=(Y^M_k)_{k \ge 0}$ and $\breve{Y}^M=(\breve{Y}^M_k)_{k \ge 0}$, by defining $Y^M_0:= x^{\circ }$, $\breve{Y}^M_0:= \breve{x}^\circ$ and for $k \ge 0$,

37 $$\begin{aligned} Y^M_{k+1}:= \Phi _{\lambda _M}(Y^M_k,U^M_{k+1}), \qquad \breve{Y}^M_{k+1}:= \breve{\Phi }_{\lambda _M}(\breve{Y}^M_k,U^M_{k+1}). \end{aligned}$$

Similarly to the previous case, $Y^M$ and $\breve{Y}^M$ are discrete-time Markov chains with transition matrices $P_{\lambda _M}(Q^{M}):= \frac{1}{\lambda _M}Q^{M} + I$ and $P_{\lambda _M}(\breve{Q}^M):= \frac{1}{\lambda _M}\breve{Q}^{M} + I$, respectively.

Now, we claim that for each $M \ge M_0$:

38 $$\begin{aligned} \mathbb {P}^M\left[ Y^M_{k \wedge S^M} \preccurlyeq _A \breve{Y}^M_{k \wedge S^M} \text { for every } k \ge 0 \right] =1. \end{aligned}$$

where $S^M:= \inf \{ k \ge 0 \,|\, Y^M_k \notin {\mathcal {X}}_M \text { or } \breve{Y}^M_k \notin {\mathcal {X}}_M\}$. In fact, (60) is equivalent to proving that $\mathbb {P}^M\left[ Y^M_{k \wedge S^M} \preccurlyeq _A \breve{Y}^M_{k \wedge S^M} \right] =1$ for every $k \ge 0$, which we do by induction. We already know that $Y^M_0 \preccurlyeq _A \breve{Y}^M_0$. Assuming the statement is true for some $k \ge 0$, to establish it for $k+1$ we distinguish between two cases. First, on $\{S^M \le k\}$, $Y^M_{(k+1) \wedge S^M} = Y^M_{k \wedge S^M} \preccurlyeq _A \breve{Y}^M_{k \wedge S^M} =\breve{Y}^M_{(k+1) \wedge S^M}$, $\mathbb {P}^M$-a.s.. Second, on $\{S^M > k\}$, $Y^M_k \in {\mathcal {X}}_M, \breve{Y}^M_k \in {\mathcal {X}}_M$, and by the induction assumption, $Y^M_k \preccurlyeq _A \breve{Y}^M_k$, $\mathbb {P}^M$-a.s.. Applying Lemma 5.1, we obtain $\mathbb {P}^M$-a.s. on $\{S^M > k\}$ that

38 $$\begin{aligned} Y^M_{(k+1) \wedge S^M} = Y^M_{k+1} =\Phi _{\lambda _M}(Y^M_k,U^M_{k+1}) \preccurlyeq _A \breve{\Phi }_{\lambda _M}(\breve{Y}^M_k,U^M_{k+1}) =\breve{Y}^M_{(k+1) \wedge S^M}, \end{aligned}$$

where we have used (58).

Now, for each $M \ge M_0$, we define the processes

39 $$\begin{aligned} X^M(t):= Y^M_{N^M(t)}, \qquad \breve{X}^{M}(t):= \breve{Y}^M_{N^M(t)}, \qquad t \ge 0. \end{aligned}$$

Then, $X^{M}$ and $\breve{X}^{M}$ are continuous-time Markov chains with infinitesimal generators $Q^M$ and $\breve{Q}^M$, respectively, and with initial conditions $X^{M}(0)=x^{\circ }$ and $\breve{X}^{M}(0)=\breve{x}^\circ$. Define $T^M:= \inf \{ t \ge 0 \,|\, X^{M}(t) \notin {\mathcal {X}}_M \text { or } \breve{X}^{M}(t) \notin {\mathcal {X}}_M\}$ and, because $Y^M$ and $\breve{Y}^M$ are the discrete time skeletons for $X^M$ and $\breve{X}^M$, we have that $\mathbb {P}^M$-a.s.

39 $$\begin{aligned} T^M = \inf \{ t \ge 0 \,|\, N^M(t) =S^M \}. \end{aligned}$$

Then, it follows from (60) that

40 $$\begin{aligned} \mathbb {P}^M\left[ X^{M}(t \wedge T^M) \preccurlyeq _A \breve{X}^{M}(t \wedge T^M) \text { for every } t \ge 0 \right] =1. \end{aligned}$$

We now prove that for every $t \ge 0$,

40 $$\begin{aligned} \mathbb {P}^M[T^M < t ] \longrightarrow 0, \quad \text { as } M \longrightarrow \infty . \end{aligned}$$

For this, let $T^M_{X^M}:= \inf \{ t \ge 0 \,|\, X^{M}(t) \notin {\mathcal {X}}_M \}$ and $T^M_{\breve{X}^M}:= \inf \{ t \ge 0 \,|\, \breve{X}^{M}(t) \notin {\mathcal {X}}_M \}$. Since $T^M =T^M_{X^M} \wedge T^M_{\breve{X}^M}$, then

41 $$\begin{aligned} \mathbb {P}^M[T^M< t ] \le \mathbb {P}^M[T^M_{X^M}< t ] + \mathbb {P}^M[T^M_{\breve{X}^M} < t], \qquad \text { for every } t \ge 0. \end{aligned}$$

Now, since $Q^M_{x,y}=Q_{x,y}$ for $x \in {\mathcal {X}}_M$ and $y \in {\mathcal {X}}$, $X^M(\cdot \wedge T^M_{X^M})$ will have the same distribution as a Markov chain with infinitesimal generator *Q* and initial condition $x^{\circ }$, stopped at the first time it leaves ${\mathcal {X}}_M$. Because of this, $T^M_{X^M}$ has the same distribution as the first time a continuous-time Markov chain with infinitesimal generator *Q* leaves ${\mathcal {X}}_M$. Since a continuous-time Markov chain with infinitesimal generator *Q* has been assumed to not explode in finite time, we obtain that $\mathbb {P}^M[T^M_{X^M} < t ] \longrightarrow 0$ as $M \rightarrow \infty$. Similar reasoning holds for $T^M_{\breve{X}^M}$. Combining with (66), we obtain (65).

Denote by ${\mathcal {D}}([0,\infty ),{\mathcal {X}}^2)$ the space of right-continuous functions from $[0,\infty )$ into ${\mathcal {X}}^2$ that also have finite left-limits. As usual, this space is endowed with Skorokhod’s $J_1$ topology. The pair $(X^M,\breve{X}^M)$ have paths in ${\mathcal {D}}([0,\infty ),{\mathcal {X}}^2)$ and we obtain $(X,\breve{X})$ as a limit in distribution of $(X^M,\breve{X}^M)$ as $M \rightarrow \infty$. We first verify that the sequence of processes $\{(X^{M},\breve{X}^{M})\}_{M \ge M_0}$ is tight. For this, it suffices to check that each sequence $\{X^{M}\}_{M \ge M_0}$ and $\{\breve{X}^{M}\}_{M \ge M_0}$ is tight, which we do by means of Theorem 7.2 in Chapter 3 of Ethier and Kurtz (1986). Condition (*a*) there (compact containment) is satisfied, because of (65) and because for ${\tilde{M}} \ge M \ge M_0$ we have that $X^{{\tilde{M}}}(\cdot \wedge T^M_{X^{{\tilde{M}}}})$ under $\mathbb {P}^{{\tilde{M}}}$ has the same law as $X^M(\cdot \wedge T^M_{X^M})$ under $\mathbb {P}^M$, where $T^M_{X^{{\tilde{M}}}}:= \inf \{t \ge 0 \,|\, X^{{\tilde{M}}}(t) \notin {\mathcal {X}}_M\}$. To verify condition (*b*) in Theorem 7.2 of Ethier and Kurtz (1986), for $t_0 > 0$ fixed and $\eta > 0$, let $M_{\eta } \ge M_0$ be such that $\mathbb {P}^M\left[ T^M_{X^M} < t_0\right] \le \frac{\eta }{2}$ for all $M \ge M_{\eta }$. Then,

$$\begin{aligned} \mathbb {P}^M\left[ w'(X^M,\delta ,t_0) \ge \eta \right]&\le \mathbb {P}^M\left[ w'(X^M,\delta ,t_0) \ge \eta \,;\, T^M_{X^M} \ge t_0\right] + \mathbb {P}^M\left[ T^M_{X^M} < t_0\right] \\&\le {\tilde{\mathbb {P}}}\left[ w'({\tilde{X}},\delta ,t_0) \ge \eta \,;\, \tau _{{\tilde{X}}}^M \ge t_0\right] + \frac{\eta }{2} \\&\le {\tilde{\mathbb {P}}}[w'({\tilde{X}},\delta ,t_0) \ge \eta ] + \frac{\eta }{2}, \end{aligned}$$

where $w'(\cdot ,\cdot ,\cdot )$ is the modulus of continuity, as defined in Equation (6.2), Chapter 3 of Ethier and Kurtz (1986), ${\tilde{X}}$ under ${\tilde{\mathbb {P}}}$ is a realization of the Markov chain associated with the infinitesimal generator *Q* that starts with $x^{\circ }$, and $\tau _{{\tilde{X}}}^M:= \inf \{ t \ge 0 \,|\, {\tilde{X}}(t) \notin {\mathcal {X}}_M \}$. Since ${\tilde{X}}$ under ${\tilde{\mathbb {P}}}$ is a single process with right-continuous paths having finite left-limits, the tightness applies to it and so the term ${\tilde{\mathbb {P}}}[w'({\tilde{X}},\delta ,t_0) \ge \eta ]$ can be made less than $\frac{\eta }{2}$ by choosing $\delta$ sufficiently small and so condition (*b*) of Theorem 7.2 of Ethier and Kurtz (1986) is satisfied. It follows that $\{X^{M}\}_{M \ge M_0}$ is tight. Similar reasoning yields tightness for $\{\breve{X}^{M}\}_{M \ge M_0}$.

It follows that there exists a probability space $(\Omega ,{\mathcal {F}},\mathbb {P})$ with two processes *X* and $\breve{X}$ defined there, having paths that are right-continuous with finite left-limits, and a subsequence $\{M_k\}_{k \ge 1}$ such that $M_k \rightarrow \infty$ as $k \rightarrow \infty$, and the sequence $\{(X^{M_k},\breve{X}^{M_k})\}_{k \ge 1}$ converges in distribution to the pair of processes $(X,\breve{X})$. To identify the law of the limit, note that since $\{Q^{M_k}\}_{k \ge 1}$ converges pointwise to *Q*, for any function *f* with bounded support in ${\mathcal {X}}$, $f(X(t)) - \int _0^t Qf(X(s))ds$ will inherit the martingale property of $f(X^{M_k}(t)) - \int _0^t Q^{M_k}f(X^{M_k}(s))ds$. It follows from the martingale characterization that *X* is a continuous-time Markov chain with infinitesimal generator *Q* (see Chapter 4 in Ethier and Kurtz (1986)). Similarly, $\breve{X}$ will be a continuous-time Markov chain with infinitesimal generator $\breve{Q}$. In addition, the processes have inherited initial conditions $X(0)=x^{\circ }$ and $\breve{X}(0)=\breve{x}^\circ$.

Finally, to show that (12) holds, consider the set

41 $$\begin{aligned} F = \{ (f,g) \in {\mathcal {D}}([0,\infty ),{\mathcal {X}}^2) \,|\, f(t) \preccurlyeq _A g(t) \text { for all } t \ge 0 \}, \end{aligned}$$

which is closed in the Skorokhod topology. From (64), we know that the stopped processes satisfy $\mathbb {P}^{M_k}[(X^{M_k}(\cdot \wedge T^{M_k}),\breve{X}^{M_k}(\cdot \wedge T^{M_k})) \in F] =1$ for every $k \ge 1$. Furthermore, from (65) we know that $T^{M_k} \longrightarrow \infty$ in probability as $k \rightarrow \infty$. The reader may verify that this last fact, along with the convergence of $(X^{M_k},\breve{X}^{M_k})$ to $(X,\breve{X})$, implies that $(X^{M_k}(\cdot \wedge T^{M_k}),\breve{X}^{M_k}(\cdot \wedge T^{M_k}))$ converges in distribution to $(X,\breve{X})$ as $k \rightarrow \infty$. By the Portmanteau Theorem (see Theorem 2.1 in Billingsley (1999)),

42 $$\begin{aligned} 1=\limsup _{k \rightarrow \infty } \mathbb {P}^{M_k}[(X^{M_k}(\cdot \wedge T^{M_k}),\breve{X}^{M_k}(\cdot \wedge T^{M_k})) \in F] \le \mathbb {P}[(X,\breve{X}) \in F] \end{aligned}$$

and we obtain (12).

#### Remark 5.1

The proof of Theorem 3.1 provides a method to simulate the sample paths for the continuous-time Markov chains *X* and $\breve{X}$ in a coupled manner for the case where (42) holds. Roughly speaking, the procedure consists of determining $\lambda > 0$ as in (43), $\Phi _{\lambda },\breve{\Phi }_{\lambda }$ as in (45), $Y,\breve{Y}$ as in (53) and $X,\breve{X}$ as in (54). For the benefit of the reader, this method is described as an algorithm in SI - Section S.4, which yields coupled sample paths under the assumptions of Theorem 3.2, 3.3 and S.2.

### Proof of Theorem 3.2

By Theorem 3.1, it suffices to prove that for every $x,y \in {\mathcal {X}}$ such that $x \preccurlyeq _A y$, conditions (10) and (11) hold. For this, we make some observations first. Consider $x,y \in {\mathcal {X}}$ such that $x \preccurlyeq _A y$ and let $1 \le j \le n$. Observe that $x \preccurlyeq _A y + v_j$ will hold if and only if $A(y+v_j -x) \ge 0$ which is equivalent to:

43 $$\begin{aligned} \langle A_{i\bullet },y -x\rangle + \langle A_{i\bullet },v_j\rangle \ge 0, \qquad \text {for every } 1 \le i \le m. \end{aligned}$$

Similarly, $x + v_j \preccurlyeq _A y$ will hold if and only if

44 $$\begin{aligned} \langle A_{i\bullet },y -x\rangle - \langle A_{i\bullet },v_j\rangle \ge 0, \qquad \text {for every } 1 \le i \le m. \end{aligned}$$

Since $x \preccurlyeq _A y$, then $\langle A_{i\bullet },y-x\rangle \ge 0$ for every $1 \le i \le m$. Now, consider $i \in \{1,...,m\}$ such that $\langle A_{i\bullet },y-x\rangle >0$. Since $A \in \mathbb {Z}^{m \times d}$ and $y-x \in \mathbb {Z}^d$, then $\langle A_{i\bullet },y-x\rangle \ge 1$. This yields that

45 $$\begin{aligned} \langle A_{i\bullet },y-x\rangle + \langle A_{i\bullet },v_j\rangle \ge 1 + \langle A_{i\bullet },v_j\rangle \ge 0, \end{aligned}$$

since $\langle A_{i\bullet },v_j\rangle \in \{-1,0,1\}$. Similarly, $\langle A_{i\bullet },y-x\rangle - \langle A_{i\bullet },v_j\rangle \ge 1 - \langle A_{i\bullet },v_j\rangle \ge 0$. By observing that the interior of $K_A +x$ is of the form ${{\,\textrm{int}\,}}(K_A + x) = \{ y \in \mathbb {R}^d \,|\, Ax < Ay\}$, the latter argument shows that for every $x \in {\mathcal {X}}$ and $y \in {{\,\textrm{int}\,}}(K_A + x) \cap {\mathcal {X}}$, we have

46 $$\begin{aligned} x \preccurlyeq _A y + v_j \text { and } x + v_j \preccurlyeq _A y, \qquad \text { for every } 1 \le j \le n. \end{aligned}$$

Now, lets check condition (10). For this, let $x,y \in {\mathcal {X}}$ be such that $x \preccurlyeq _A y$ and let $1 \le j \le n$ be such that $y + v_j \in {\mathcal {X}}\backslash (K_A + x)$. By (72), $y \notin {{\,\textrm{int}\,}}(K_A + x)$ and since $y \in K_A + x$, we must have $y \in \partial (K_A + x)= \{ z \in K_A +x \,|\, \langle A_{i\bullet },z\rangle = \langle A_{i\bullet },x\rangle \; \mathrm {for\; some\;} 1\le i \le m\}$, the boundary of $K_A + x$. Consider the set of indices ${{\textbf {K}}}_{y}:= \{ i\,|\, \langle A_{i\bullet },y\rangle = \langle A_{i\bullet },x\rangle , 1\le i\le m \}$, which is non-empty. Observe that for every $i \notin {{\textbf {K}}}_y$, $\langle A_{i\bullet },y-x\rangle > 0$ and from (71), $\langle A_{i\bullet },(y+v_j)-x\rangle \ge 0$, while for $i \in {{\textbf {K}}}_y$, $\langle A_{i\bullet },(y+v_j)-x\rangle = \langle A_{i\bullet },v_j\rangle$. From this, we can infer that there exists an $i_k \in {{\textbf {K}}}_y$ such that $\langle A_{i_k\bullet },v_j\rangle < 0$. Indeed, if this was not the case, then $\langle A_{i\bullet },(y+v_j)-x\rangle \ge 0$ for every $i \in {{\textbf {K}}}_y$ and consequently (69) would hold. This contradicts the fact that $y + v_j \notin K_A + x$. By (14), we know that $\langle A_{i_k\bullet },v_j\rangle < 0$ implies $\breve{\Upsilon }_j(y) \le \Upsilon _j(x)$ and we conclude that (10) holds.

To check condition (11), let $x,y \in {\mathcal {X}}$ be such that $x \preccurlyeq _A y$ and let $1 \le j \le n$ be such that $x + v_j \in {\mathcal {X}}$ and $y \notin K_A + x + v_j$. Again, by (72), we obtain that $y \in \partial (K_A + x)$ and ${{\textbf {K}}}_{y} \ne \emptyset$. For every $i \notin {{\textbf {K}}}_y$, $\langle A_{i\bullet },y-(x+v_j)\rangle \ge 0$, while for $i \in {{\textbf {K}}}_y$, $\langle A_{i\bullet },y-(x+v_j)\rangle = -\langle A_{i\bullet },v_j\rangle$. From this, we can infer that there exists an $i_k \in {{\textbf {K}}}_y$ such that $\langle A_{i_k\bullet },v_j\rangle > 0$. By (15), we know that $\langle A_{i_k\bullet },v_j\rangle > 0$ implies $\breve{\Upsilon }_j(y) \ge \Upsilon _j(x)$ and we conclude that (11) holds.

### Proof of Theorem 3.3

The proof of this result uses similar general ideas to the ones used in the proof of Theorem 3.1. However, since the conditions involve sums, the construction is somewhat different and more complex and we provide the details below. Let us consider again a non-empty set ${\mathcal {X}}\subseteq \mathbb {Z}_+^d$, a collection of distinct vectors $v_1,\ldots ,v_n$ in $\mathbb {Z}^d {\setminus } \{0\}$ and two collections of nonnegative functions on ${\mathcal {X}}$, $\Upsilon =(\Upsilon _1, \dots ,\Upsilon _n)$ and $\breve{\Upsilon }= (\breve{\Upsilon }_1, \dots ,\breve{\Upsilon }_n)$ such that (8) holds. In the following, let $A \in \mathbb {Z}^{m \times d}$ be a matrix with nonzero rows such that condition (i) of Theorem 3.3 holds.

We initially assume that $\sup _{x \in {\mathcal {X}}} \Upsilon _j(x) < \infty$ and $\sup _{x \in {\mathcal {X}}} \breve{\Upsilon }_j(x) < \infty$ for every $1 \le j \le n$, and let $\lambda > 0$ such that (43) holds. We shall relax these assumptions later. We start by defining functions analogous to $\Phi _{\lambda }$ and $\breve{\Phi }_{\lambda }$ as defined in (45), although this time, the construction is more involved.

Recall that *s* denotes the size of the set $\{Av_j \,|\, 1 \le j \le n\}$ and that the index sets $G^k \ne \emptyset$, $1 \le k \le s$, defined in (17), are such that $Av_j =\eta ^k$ for all $j \in G^k$, $1 \le k \le s$. Consider a bijection $\sigma :\{1,\ldots ,n\} \longrightarrow \{1,\ldots ,n\}$ such that the vectors $v_{\sigma (1)},\ldots , v_{\sigma (n)}$ have the property that the first $|G^1|$ vectors have indices in $G^1$, the next $|G^2|$ vectors have indices in $G^2$, and so on. More precisely, the bijection $\sigma$ is such that for $1 \le k \le s$, $Av_{\sigma (q)}=\eta ^k$, whenever $\sum _{\ell =1}^{k-1}|G^{\ell }|+1 \le q \le \sum _{\ell =1}^{k}|G^{\ell }|$. Recall for this that a sum over an empty set is taken to equal zero.

For $x \in {\mathcal {X}}$, we define a family of intervals $\{I^k(x)\,|\, 1 \le k \le s\}$ as follows.

Let $p_0:= 0$, and for $1 \le k \le s$, inductively define $p_k:=\sum _{\ell =1}^{k}|G^{\ell }|$, and

47 $$\begin{aligned} I^k(x):= \bigcup _{q=p_{k-1}+1}^{p_k} I^k_{q}(x), \end{aligned}$$

where for $$p_{k-1}+1\le q \le p_k$$,

48 $$\begin{aligned} I^k_{q}(x):=\left[ \frac{p_{k-1}}{n}+\sum _{\ell =p_{k-1}+1}^{q-1}\frac{\Upsilon _{\sigma (\ell )}(x)}{\lambda },\frac{p_{k-1}}{n} +\sum _{\ell =p_{k-1}+1}^{q}\frac{\Upsilon _{\sigma (\ell )}(x)}{\lambda }\right) . \end{aligned}$$

The sets $I^k_q(x)$, with $1\le k\le s$ and $p_{k-1}+1 \le q \le p_k$, are mutually disjoint, and by (43), the length of $I^k(x)$ is less than $\frac{p_k-p_{k-1}}{n}=\frac{|G^k|}{n}$, and so the sum of the lengths of $\{I^k(x)\,|\, 1 \le k \le s\}$ is less than $\frac{1}{n} \sum _{k=1}^s |G^k|=1$. Now, let us define $\Psi _{\lambda }(\cdot ,\cdot ): {\mathcal {X}}\times [0,1] \longrightarrow {\mathcal {X}}$ by

49 $$\begin{aligned} \Psi _{\lambda }(x,u):= x + \sum _{k=1}^{s} \sum _{q=p_{k-1}+1}^{p_k} v_{\sigma (q)}\mathbbm {1}_{I^k_q(x)}(u), \qquad x \in {\mathcal {X}}, \; u \in [0,1]. \end{aligned}$$

Note that $Av_{\sigma (q)}=\eta ^k$ for $p_{k-1}+1 \le q \le p_k$, $1 \le k \le s$. From the above properties of $I^k_q(x)$, we have that for any $u \in [0,1]$, either $u \notin \bigcup _{k=1}^{s} I^k(x)$ or $u \in I^k_{q}(x)$ for exactly one *k* and *q* such that $I^k_{q}(x) \ne \emptyset$. The latter condition implies, by (74), that $\Upsilon _{\sigma (q)}(x) > 0$ and then, by (8), $x + v_{\sigma (q)} \in {\mathcal {X}}$. This shows that $\Psi _{\lambda }(\cdot ,\cdot )$ is well-defined as an ${\mathcal {X}}$-valued function.

In an analogous manner to that above, we can define intervals $\breve{I}^k(x)$, $\breve{I}^k_q(x), \, 1 \le k \le s, \, p_{k-1}+1 \le q \le p_k, \, x \in {\mathcal {X}}$ and a function $\breve{\Psi }_{\lambda }:{\mathcal {X}}\times [0,1] \longrightarrow {\mathcal {X}}$, as in (73) – (75), but with $\breve{\Upsilon }_j(x)$, $\breve{I}^k(x)$, $\breve{I}^k_q(x)$, $\breve{\Psi }_{\lambda }$ in place of $\Upsilon _j(x)$, $I^k(x)$, $I^k_q(x)$, $\Psi _{\lambda }$.

#### Lemma 5.2

Suppose that $x,y \in {\mathcal {X}}$ are such that $x \preccurlyeq _A y$ and the following hold: whenever $y \in \partial _i(K_A+x) \cap {\mathcal {X}}$ for some $1 \le i \le m$, we have

50 $$\begin{aligned} \sum _{j \in G^{k}} \breve{\Upsilon }_j(y) \le \sum _{j \in G^{k}} \Upsilon _j(x), \quad \text {for every } k \text { such that } \eta ^k_i <0, \end{aligned}$$

and

51 $$\begin{aligned} \sum _{j \in G^{k} } \breve{\Upsilon }_j(y) \ge \sum _{j \in G^{k}} \Upsilon _j(x), \quad \text {for every } k \text { such that } \eta ^k_i >0. \end{aligned}$$

Then, for each $u \in [0,1]$,

52 $$\begin{aligned} \Psi _{\lambda }(x,u) \preccurlyeq _A \breve{\Psi }_{\lambda }(y,u). \end{aligned}$$

#### Proof

First, we note that $\Psi _{\lambda }, \breve{\Psi }_{\lambda }$ have the following properties: for every $u \in [0,1]$, $1\le k \le s$, $j \in G^k$,

53 $$\begin{aligned} \text { if } \Psi _{\lambda }(x,u) = x + v_j \text {, then } \breve{\Psi }_{\lambda }(y,u) \in \{y + v_\ell : \ell \in G^k \} \cup \{y\}, \end{aligned}$$

since $I_{\sigma ^{-1}(j)}^k(x), \breve{I}_{\sigma ^{-1}(\ell )}^k(y) \subseteq [\frac{p_{k-1}}{n},\frac{p_{k}}{n})$ for $\ell \in G^k$. Similarly,

54 $$\begin{aligned} \text { if } \breve{\Psi }_{\lambda }(y,u) = y +v_j \text {, then } \Psi _{\lambda }(x,u) \in \{x + v_\ell : \ell \in G^k\} \cup \{x\}. \end{aligned}$$

Furthermore, for $1\le k \le s$, $j \in G^k$, if $\sum _{\ell \in G^{k}} \breve{\Upsilon }_\ell (y) \ge \sum _{\ell \in G^{k}} \Upsilon _\ell (x)$, then

55 $$\begin{aligned} \Psi _{\lambda }(x,u) = x +v_j \text { implies that } \breve{\Psi }_{\lambda }(y,u) =y +v_\ell \, \text { for some } \, \ell \in G^k, \end{aligned}$$

since under the condition, $I^k(x) \subseteq \breve{I}^k(y)$. Similarly, if $\sum _{\ell \in G^{k}} \breve{\Upsilon }_\ell (y) \le \sum _{\ell \in G^{k}} \Upsilon _\ell (x)$, then

56 $$\begin{aligned} \breve{\Psi }_{\lambda }(y,u) = y +v_j \text { implies that } \Psi _{\lambda }(x,u) =x +v_\ell \, \text { for some } \, \ell \in G^k. \end{aligned}$$

We also have that, for $1 \le k \le s$ and $j \in G^k$, $x \preccurlyeq _A y + v_j$ if and only if

57 $$\begin{aligned} \langle A_{i\bullet },y -x\rangle + \langle A_{i\bullet },v_j\rangle \ge 0, \qquad \text {for every } 1 \le i \le m. \end{aligned}$$

Similarly, $x + v_j \preccurlyeq _A y$ if and only if

58 $$\begin{aligned} \langle A_{i\bullet },y -x\rangle - \langle A_{i\bullet },v_j\rangle \ge 0, \qquad \text {for every } 1 \le i \le m. \end{aligned}$$

Furthermore, for $1 \le k \le s$ and $j,\ell \in G^k$, since $Av_j=Av_{\ell }$ and $x \preccurlyeq _A y$, then

59 $$\begin{aligned} \langle A_{i\bullet },y -x\rangle + \langle A_{i\bullet },(v_j-v_\ell )\rangle = \langle A_{i\bullet },y-x\rangle \ge 0, \quad \text {for every } 1 \le i \le m.\qquad \end{aligned}$$

To prove (78), we first consider the situation where $y \in {{\,\textrm{int}\,}}(K_A + x) = \{ w \in \mathbb {R}^d \,|\, Ax < Aw\}$. Then, for each $1 \le i \le m$, $\langle A_{i\bullet },y-x\rangle >0$ and since $A \in \mathbb {Z}^{m \times d}$ and $y-x \in \mathbb {Z}^d$, we have $\langle A_{i\bullet },y-x\rangle \ge 1$. This implies that for $1 \le k \le s$ and $j \in G^k$,

60 $$\begin{aligned} \langle A_{i\bullet },y-x\rangle + \langle A_{i\bullet },v_j\rangle \ge 1 + \langle A_{i\bullet },v_j\rangle \ge 0, \qquad \text {for every } 1 \le i \le m, \end{aligned}$$

since $\langle A_{i\bullet },v_j\rangle \in \{-1,0,1\}$ by condition (*i*) of Theorem 3.3. Similarly, for $1 \le k \le s$ and $j \in G^k$,

61 $$\begin{aligned} \langle A_{i\bullet },y-x\rangle - \langle A_{i\bullet },v_j\rangle \ge 1 - \langle A_{i\bullet },v_j\rangle \ge 0, \qquad \text {for every } 1 \le i \le m. \end{aligned}$$

It follows from (85) – (87) that if $y \in {{\,\textrm{int}\,}}(K_A + x) \cap {\mathcal {X}}$, then for any $1 \le k \le s$ and $j, \ell \in G^k$:

62 $$\begin{aligned} x \preccurlyeq _A y + v_j, \, x + v_j \preccurlyeq _A y \, \text { and } \, x + v_\ell \preccurlyeq _A y + v_j. \end{aligned}$$

We also have, by assumption, that $x \preccurlyeq _A y$. It follows that if $y \in {{\,\textrm{int}\,}}(K_A + x) \cap {\mathcal {X}}$, then $\{x,x +v_\ell \,|\, \ell \in G^k\}\preccurlyeq _A \{y,y+v_j \,|\, j \in G^k\}$ for $1 \le k \le s$ and consequently (78) holds for all $u \in [0,1]$.

Now, we turn to the other situation where $y \in \partial _i(K_A+x) \cap {\mathcal {X}}$ for some $1 \le i \le m$. Then ${{\textbf {K}}}_{y}:= \{ i\,|\, \langle A_{i\bullet },y\rangle = \langle A_{i\bullet },x\rangle , 1\le i\le m \}$ is non-empty. Let $u\in [0,1]$. We consider two cases.

**Case 1:** $\breve{\Psi }_{\lambda }(y,u) = y +v_j$ for some $1 \le j \le n$.

Fix such an index *j*. Consider the unique $1 \le k \le s$ such that $j \in G^k$. Then, by (80), either $\Psi _{\lambda }(x,u) = x + v_\ell$ for some $\ell \in G^k$, or $\Psi _{\lambda }(x,u) = x$.

- If $\Psi _{\lambda }(x,u) = x + v_\ell$ for some $\ell \in G^k$, then, since $x \preccurlyeq _A y$ and $Av_j = Av_\ell$, we have $x + v_\ell \preccurlyeq _A y +v_j$. Hence, $\Psi _{\lambda }(x,u) \preccurlyeq _A \breve{\Psi }_{\lambda }(y,u)$ and (78) holds.
- If $\Psi _{\lambda }(x,u) = x$, we claim that $y + v_j \in K_A + x$. To see this, observe that for every $i \notin {{\textbf {K}}}_y$, $\langle A_{i\bullet },y-x\rangle > 0$ and as for (86), $\langle A_{i\bullet },(y+v_j)-x\rangle \ge 0$, while for $i \in {{\textbf {K}}}_y$, $\langle A_{i\bullet },(y+v_j)-x\rangle = \langle A_{i\bullet },v_j\rangle \in \{-1,0,1\}$. For each $i \in {{\textbf {K}}}_y$, if $\langle A_{i\bullet },v_j\rangle =-1$, then by (76), we would have $\sum _{\ell \in G^{k}} \breve{\Upsilon }_\ell (y) \le \sum _{\ell \in G^{k}} \Upsilon _\ell (x)$, which would imply that $\Psi _{\lambda }(x,u) = x +v_\ell$ for some $\ell \in G^k$, but this contradicts the assumption that $\Psi _{\lambda }(x,u) = x$. So we must have $\langle A_{i\bullet },v_j\rangle \ge 0$ and hence $\langle A_{i\bullet },(y+v_j)-x\rangle \ge 0$ for all $i \in {{\textbf {K}}}_y$. Thus, $y + v_j \in K_A + x$ and so $\Psi _{\lambda }(x,u) = x \preccurlyeq _A y + v_j = \breve{\Psi }_{\lambda }(y,u)$ holds.

**Case 2:** $\breve{\Psi }_{\lambda }(y,u) = y$. Again, we consider two subcases.

- If $\Psi _{\lambda }(x,u)=x$, then (78) holds, because $x \preccurlyeq _A y$.
- If $\Psi _{\lambda }(x,u) = x +v_j$ for some $1 \le j \le n$, we claim that $y \in K_A + x + v_j$ for the corresponding value of *j*. To see this, fix the value of *j* for which $\Psi _{\lambda }(x,u) = x +v_j$, let $1 \le k \le s$ be such that $j \in G^k$, and observe that for every $i \notin {{\textbf {K}}}_y$, $\langle A_{i\bullet },y-x\rangle > 0$ and as for (87), $\langle A_{i\bullet },y-(x+v_j)\rangle \ge 0$, while for $i \in {{\textbf {K}}}_y$, $\langle A_{i\bullet },y-(x+v_j)\rangle = - \langle A_{i\bullet },v_j\rangle \in \{-1,0,1\}$. For each $i \in {{\textbf {K}}}_y$, if $\langle A_{i\bullet },v_j\rangle =1$, then by (77), we would have $\sum _{\ell \in G^{k} } \breve{\Upsilon }_\ell (y) \ge \sum _{\ell \in G^{k}} \Upsilon _\ell (x)$, which would imply that $\breve{\Psi }_{\lambda }(y,u) = y +v_\ell$ for some $\ell \in G^k$. This would contradict the assumption that $\breve{\Psi }_{\lambda }(y,u) = y$. So we must have $\langle A_{i\bullet },v_j\rangle \le 0$ and hence $\langle A_{i\bullet },y-(x+v_j)\rangle =\langle A_{i\bullet },y-x\rangle -\langle A_{i\bullet },v_j\rangle \ge 0$ for all $i \in {{\textbf {K}}}_y$. Thus, we have $y \in K_A + x + v_j$ and then $\Psi _{\lambda }(x,u) = x + v_j \preccurlyeq _A y = \breve{\Psi }_{\lambda }(y,u)$.

$$\square $$

In order to prove Theorem 3.3, from here on we can follow a similar procedure to the one used in the proof of Theorem 3.1 after Lemma 5.1 was proved there. For the case where (42) holds, we define two discrete-time processes, $Y=(Y_k)_{k \ge 0}$ and $\breve{Y}=(\breve{Y}_k)_{k \ge 0}$, by defining $Y_0:= x^{\circ }$, $\breve{Y}_0:= \breve{x}^\circ$, and for $k \ge 0$,

63 $$\begin{aligned} Y_{k+1}:= \Psi _{\lambda }(Y_k,U_{k+1}), \qquad \breve{Y}_{k+1}:= \breve{\Psi }_{\lambda }(\breve{Y}_k,U_{k+1}), \end{aligned}$$

and define *X* and $\breve{X}$ using these and an independent Poisson process *N* as in (54). For the case where (42) does not hold, we can use a truncation procedure similar to that for Theorem 3.1. In both cases, we use Lemma 5.2 instead of Lemma 5.1.

## Conclusion

In this work, we first reviewed the concept of Stochastic Chemical Reaction Networks (SCRNs), a class of continuous-time Markov chain models frequently used to describe the stochastic behavior of chemical reaction systems. We also gave the definitions of preorder and increasing set considered in this paper. In Sect. 3.2, we presented the main theoretical results of this paper. We first derived, by exploiting uniformization and then coupling of stochastic processes (see Grassmann (1977) and Keilson (1979)), three theorems which give practical sufficient conditions for stochastic dominance of one continuous-time Markov chain over another. More precisely, these theorems provide conditions under which, when one or more parameters is changed monotonically, the system is almost surely “higher” with respect to a certain preorder. While the first theorem (Theorem 3.1) can be used for any SCRN, it has extensive conditions to check. The second set of theorems (Theorems 3.2, 3.3) can be used for more specific SCRN classes, but they have assumptions that only need to be checked at the boundary of certain translated convex cones. All these theorems can be applied to SCRNs with either finite or countably many states. In Sect. 3.3, we exploited these tools to develop two theorems to specifically study the monotonicity properties of stationary distributions and mean first passage times depending on system parameters.

Subsequently, in Sect. 4, we presented some illustrative examples to highlight the advantages of using our theoretical tools in order to study the stochastic behavior of SCRNs. Specifically, we focused on two common models for enzymatic kinetics (see Michaelis and Menten (1913), Kang et al. (2019), Del Vecchio and Murray (2014) and Anderson et al. (2010)), on a model inspired by Braess’s paradox (see Calvert et al. (1997)) and on a recently developed model describing the main interactions among histone modifications alone, and together with an expressed protein (see Bruno et al. (2022)). In these illustrative examples we see that our sufficient conditions can be easy to check and our results can be also used to study networks with a countably infinite number of states. Furthermore, the conclusions obtained by using our theorems are true for trajectories of the Markov chains, yielding results for both transient and steady-state behavior.

Overall, in this paper we derived and presented theorems that can be used for the theoretical study of monotonicity of SCRNs associated to a variety of chemical reaction systems. Future work will include the adaptation of our theoretical tools to other forms of monotonicity for SCRNs (see Definition 5.1.1 in Muller and Stoyan (2002) as an example), the investigation of possible correlations between the network graph properties and the monotonicity properties of the SCRN (extension of the work of Angeli et al. (2006) to SCRNs), and the application of our results to deterministic chemical reaction network through appropriate limits.

## **Supplementary information (SI) file:**

File containing detailed mathematical derivations for some of our examples, a generalization of Theorem 3.3, and an algorithm for coupled stochastic simulation.

## Supplementary Information

Below is the link to the electronic supplementary material.

Supplementary file 1 (pdf 462 KB)

## Data Availability

Data sharing not applicable to this article as no datasets were generated or analyzed during the current study.

## Footnotes

1 Note that *Q* is sometimes called an infinitesimal transition matrix although it may have countably many “rows" and “columns". The entries $Q_{x,y}$ for $x \ne y$ are the infinitesimal transition rates of going from *x* to *y*: $\mathbb {P}[X(t+h) = y | X(t)=x] = Q_{x,y}h + o(h)$ as $h \rightarrow 0$.

2 Here, for convenience of notation, let $A_{i\bullet }$ denote the row vector corresponding to the *i*-th row of *A*, for $1 \le i \le m$. In this article, we will adopt the convention of considering the inner product $\langle \cdot ,\cdot \rangle$ as a function of a row vector in its first entry and as a function of a column vector in the second entry. In particular, $\langle A_{i\bullet },x\rangle = \sum _{k=1}^d A_{ik}x_k$.

3 The fact that *A* does not contain zero rows allows for equation (13) to hold. In fact, let $A \in \mathbb {R}^{m \times d}$ be a matrix that is not identically zero and let ${\bar{A}}$ be the matrix obtained from *A* by erasing any rows that contain all zeros. Then, for $x,y \in \mathbb {R}^d$, $A(y-x) \ge 0$ if and only if ${\bar{A}}(y-x) \ge 0$, and so $K_A+x = K_{{\bar{A}}}+x$ and $x \preccurlyeq _A y$ if and only if $x \preccurlyeq _{{\bar{A}}} y$. However, if *A* contains a row $A_{i\bullet }$ such that $A_{i\bullet }=0$, then $\partial _i(K_A +x) = K_A +x$ and if $K_A +x$ has non empty interior, then equation (13) will not hold. Consequently, we have made the assumption that *A* has no zero rows.

4 These “matrices" may have countably many rows and columns, in which case they could be considered as operators on $\ell ^{\infty }$. For convenience, we still call them “matrices” here.

5 Stochastic here means that all entries take values in [0, 1] and all row sums equal one.
