# Supplementary material for: Comparison Theorems for Stochastic Chemical Reaction Networks
Source: Bull Math Biol. 2023 Mar 31;85(5):39. doi: 10.1007/s11538-023-01136-5 (PMC10066174; doi:10.1007/s11538-023-01136-5)
Supplement: Supplementary file 1 — (pdf 462 KB) [file 11538_2023_1136_MOESM1_ESM.pdf]

Comparison Theorems for Stochastic Chemical Reaction Networks

Felipe A. Campos[1,*], Simone Bruno[2,*], Yi Fu[1], Domitilla Del Vecchio[2], and Ruth J. Williams[1]

[1] *Department of Mathematics, University of California, San Diego, 9500 Gilman Drive, La Jolla CA 92093-0112. Email:* `(fcamposv,yif064,rjwilliams)@ucsd.edu`

[2] *Department of Mechanical Engineering, Massachusetts Institute of Technology, 77 Massachusetts Avenue, Cambridge, MA 02139. Emails:* `(sbruno,ddv)@mit.edu`

[*] *These authors contributed equally: F. A. Campos and S. Bruno*

Supplementary Information (SI) file

S.1 Criteria for Positive Recurrence and Exponential Ergodicity with Application to Examples 4.2 and 4.5

S.1.1 Foster-Lyapunov Conditions for Positive Recurrence and Exponential Ergodicity

Here we recall fairly general conditions for positive recurrence and exponential ergodicity of a continuous-time Markov chain. Such conditions are well known and are usually referred to as Foster-Lyapunov-type conditions. We also apply these to the Markov chains in Examples 4.2 and 4.5, in subsections S.1.2 and S.1.3.

Theorem S.1. *Let X be an irreducible continuous-time Markov chain with state space $\mathcal{X}$ and infinitesimal generator Q. Suppose $V : \mathcal{X} \to \mathbb{R}_+$ is norm-like, that is $\{x \in \mathcal{X} : V(x) \leq a\}$ is compact* [6] *for each $a \in \mathbb{R}_+$. Further assume that for some $c > 0$, $d > 0$ and a compact set C,*

$$QV(x) \leq -c + d\mathbb{1}_C(x), \quad \text{for all } x \in \mathcal{X}. \tag{S.1}$$

Then, X is non-explosive and positive recurrent, and has a unique stationary distribution π. If instead of (S.1), we have that for some $c' > 0$ and $d' > 0$,

$$QV(x) \leq -c'V(x) + d', \quad \text{for all } x \in \mathcal{X}, \tag{S.2}$$

then (S.1) automatically holds and the consequences stated above hold, and in addition, the stationary distribution satisfies

$$\pi(V) = \sum_{x \in \mathcal{X}} \pi_x V(x) < \infty,$$

and there is $0 < B < \infty$ and $0 < \beta < 1$ such that for all $t \geq 0$ and $x \in \mathcal{X}$,

$$\sum_{y \in \mathcal{X}} |P_{xy}(t) - \pi_y| \leq \|P_{x\bullet}(t) - \pi\|_{V+1} \leq B(V(x)+1)\beta^t, \tag{S.3}$$

[6] Since X is a Markov chain, $\mathcal{X}$ is finite or countable, and we endow it with the usual discrete topology consisting of all subsets of $\mathcal{X}$.

where $P_{xy}(t) = \mathbb{P}[X(t) = y | X(0) = x]$ *and*

$$\|P_{x\bullet}(t) - \pi\|_{V+1} = \sup_{|g| \le V+1} \left| \sum_{y \in \mathcal{X}} (P_{xy}(t) - \pi_y) g(y) \right|.$$

Remark S.1. When the second inequality in (S.3) holds, we say that the Markov chain is exponentially ergodic in the $(V+1)$-norm.

Proof. We will verify the sufficient conditions for each of non-explosion, positive recurrence and exponential ergodicity given in Meyn & Tweedie [1]. Note that X is a Borel right-process under the definition in Sharpe [2]. In addition, since the state space is discrete, each compact set is finite and therefore petite.

For $m \in \mathbb{Z}_+$, if Q_m is the infinitesimal generator for the Markov chain X killed upon exit from $O_m = \{x \in \mathcal{X} : V(x) \le m\}$ [7], then $Q_m V(x) \le QV(x)$ for $x \in O_m$. It then follows from (S.1) that conditions (CD0) and (CD2) (with $f = 1$) in [1] hold with Q_m in place of $\mathscr{A}_m$ there. By Theorem 2.1 and Theorem 4.2 in [1], the Markov chain is non-explosive and positive recurrent, and it has a unique stationary distribution π.

On the other hand, if (S.2) holds, then (S.1) holds, using the norm like property of V. Furthermore, (S.2) implies that conditions (CD0), (CD2) (with $f = V + 1$) and (CD3) in [1] hold with Q_m in place of $\mathscr{A}_m$ there. By Theorem 2.1, Theorem 4.2 and Theorem 6.1 in [1], the Markov chain is non-explosive and positive recurrent, with a unique stationary distribution π such that $\pi(V) < \infty$, and it is exponentially ergodic in the $(V+1)$-norm, that is the second inequality in (S.3) holds for all $t \ge 0$ and $x \in \mathcal{X}$. For fixed $t \ge 0$ and $x \in \mathcal{X}$, setting $g(y) = \text{sgn}(P_{xy}(t) - \pi_y)$, for $y \in \mathcal{X}$, we have that $|g| \le 1 \le V + 1$, and

$$\sum_{y \in \mathcal{X}} |P_{xy}(t) - \pi_y| = \left| \sum_{y \in \mathcal{X}} (P_{xy}(t) - \pi_y) g(y) \right| \le \|P_{x\bullet}(t) - \pi\|_{V+1},$$

yielding the first inequality in (S.3). □

S.1.2 Application to Example 4.2

For Example 4.2, we first show that the Markov chain is irreducible. For this, consider $x^\circ = (0, 0, \mathrm{E_{tot}}, 0)$ and any fixed state $x = (x_1, x_2, x_3, \mathrm{E_{tot}} - x_3) : 0 \le x_3 \le \mathrm{E_{tot}}$. Starting at x°, by having reaction ⑤ fire $x_1 + \mathrm{E_{tot}} - x_3 + x_2$ times in succession, then having reaction ①, immediately followed by reaction ③, fire x_2 times in succession and then reaction ① fire $\mathrm{E_{tot}} - x_3$ times, without any other reactions firing, we see that the Markov chain can transition with positive probability from x° to x. Since each reaction is reversible, it also follows that the Markov chain can transition from x to x° with positive probability. Thus, the Markov chain is irreducible.

Next we will introduce a norm-like function V and show that (S.1) holds. For each $x \in \mathcal{X}$, let

$$V(x) = x_1^2 + ((2\mathrm{E_{tot}} - 1)b + 1)x_2 + bx_4^2,$$

[7] Upon exit from O_m, the killed process goes to a cemetery state Δ_m in $\mathcal{X} \backslash O_m$ where $V(\Delta_m) = \min\{V(x) : x \in \mathcal{X} \setminus O_m\}$.

where

$$b = \frac{1 + (\kappa_5 + \kappa_2 \mathrm{E_{tot}} + \kappa_3 \mathrm{E_{tot}}) + \frac{(2\kappa_5 + \kappa_6 + 2\kappa_2 \mathrm{E_{tot}})^2}{8\kappa_6}}{\kappa_2 \mathrm{E_{tot}}(2\mathrm{E_{tot}} - 1)}. \tag{S.4}$$

Notice that $b > 0$ since $\mathrm{E_{tot}} \geq 1$. Then, for each $a \in \mathbb{R}_+$, $\{x \in \mathcal{X} : V(x) \leq a\}$ consists of finitely many states, and for any $x \in \mathcal{X}$,

$$\begin{aligned}
QV(x) &= \sum_{j=1}^{6} \Upsilon_j(x) \cdot (V(x + v_j) - V(x)) \\
&= \kappa_1 x_1 x_3 \cdot (((x_1 - 1)^2 + b(x_4 + 1)^2) - (x_1^2 + b x_4^2)) \\
&\quad + \kappa_2 x_4 \cdot (((x_1 + 1)^2 + b(x_4 - 1)^2) - (x_1^2 + b x_4^2)) \\
&\quad + \kappa_3 x_4 \cdot ((((2\mathrm{E_{tot}} - 1)b + 1)(x_2 + 1) + b(x_4 - 1)^2) - (((2\mathrm{E_{tot}} - 1)b + 1)x_2 + b x_4^2)) \\
&\quad + \kappa_4 x_2 x_3 \cdot ((((2\mathrm{E_{tot}} - 1)b + 1)(x_2 - 1) + b(x_4 + 1)^2) - (((2\mathrm{E_{tot}} - 1)b + 1)x_2 + b x_4^2)) \\
&\quad + \kappa_5 \cdot ((x_1 + 1)^2 - x_1^2) + \kappa_6 x_1 \cdot ((x_1 - 1)^2 - x_1^2) \\
&= \kappa_1 x_1 x_3 \cdot (-2x_1 + 1 + b(2x_4 + 1)) + \kappa_2 x_4 \cdot (2x_1 + 1 + b(-2x_4 + 1)) \\
&\quad + \kappa_3 x_4 \cdot (((2\mathrm{E_{tot}} - 1)b + 1) + b(-2x_4 + 1)) \\
&\quad + \kappa_4 x_2 x_3 \cdot (-((2\mathrm{E_{tot}} - 1)b + 1) + b(2x_4 + 1)) \\
&\quad + \kappa_5 \cdot (2x_1 + 1) + \kappa_6 x_1 \cdot (-2x_1 + 1) \\
&= -(2\kappa_1 x_3 + 2\kappa_6) \cdot x_1^2 + (2\kappa_5 + \kappa_6 + \kappa_1(1 + b)x_3 + 2b\kappa_1 x_3 x_4 + 2\kappa_2 x_4) \cdot x_1 \\
&\quad + \kappa_4(-2b\mathrm{E_{tot}} x_3 + 2b x_3 x_4 + (2b - 1)x_3) \cdot x_2 \\
&\quad + \kappa_5 + (\kappa_2(1 + b) + \kappa_3(2b\mathrm{E_{tot}} + 1))x_4 - 2b(\kappa_2 + \kappa_3)x_4^2 \\
&= -(2\kappa_1 x_3 + 2\kappa_6) \cdot x_1^2 + (2\kappa_5 + \kappa_6 + \kappa_1(1 + b)x_3 + 2b\kappa_1 x_3 x_4 + 2\kappa_2 x_4) \cdot x_1 \\
&\quad + \kappa_4(-2b x_3^2 + (2b - 1)x_3) \cdot x_2 + \kappa_5 + (\kappa_2(1 + b) + \kappa_3(2b\mathrm{E_{tot}} + 1))x_4 - 2b(\kappa_2 + \kappa_3)x_4^2.
\end{aligned}$$

The last equality uses the fact that $x_3 = \mathrm{E_{tot}} - x_4$. For the following, we note that $-2b x_3^2 + (2b - 1)x_3 = (-2b x_3 + (2b - 1))x_3 \leq -1$ when $x_3 \geq 1$.

We now consider two cases for $QV(x)$: when $x_3 = 0$ and $x_3 > 0$. For the first case, when $x_3 = 0$, we have $x_4 = \mathrm{E_{tot}}$ and

$$QV(x) = -2\kappa_6 \cdot x_1^2 + (2\kappa_5 + \kappa_6 + 2\kappa_2 \mathrm{E_{tot}}) \cdot x_1 + \kappa_5 + (\kappa_2(1 + b) + \kappa_3)\mathrm{E_{tot}} - 2b\kappa_2 \mathrm{E_{tot}}^2.$$

As a quadratic function, the last expression is bounded above by

$$b\kappa_2 \mathrm{E_{tot}}(1 - 2\mathrm{E_{tot}}) + (\kappa_5 + \kappa_2 \mathrm{E_{tot}} + \kappa_3 \mathrm{E_{tot}}) + \frac{(2\kappa_5 + \kappa_6 + 2\kappa_2 \mathrm{E_{tot}})^2}{8\kappa_6} = -1,$$

since b is chosen as in (S.4). For the second case when $x_3 \in \{1, \ldots, \mathrm{E_{tot}}\}$, we have

$$\begin{aligned}
QV(x) &\leq -2\kappa_6 \cdot x_1^2 + (2\kappa_5 + \kappa_6 + \kappa_1(1 + b)x_3 + 2b\kappa_1 x_3 x_4 + 2\kappa_2 x_4) \cdot x_1 \\
&\quad - \kappa_4 \cdot x_2 + \kappa_5 + (\kappa_2(1 + b) + \kappa_3(2b\mathrm{E_{tot}} + 1))x_4 - 2b(\kappa_2 + \kappa_3)x_4^2 \\
&\leq -2\kappa_6 \cdot x_1^2 + \kappa_6 \cdot x_1^2 + \left(\frac{2\kappa_5 + \kappa_6 + \kappa_1(1 + b)x_3 + 2b\kappa_1 x_3 x_4 + 2\kappa_2 x_4}{2\sqrt{\kappa_6}}\right)^2 \\
&\quad - \kappa_4 \cdot x_2 + \kappa_5 + (\kappa_2(1 + b) + \kappa_3(2b\mathrm{E_{tot}} + 1))x_4 - 2b(\kappa_2 + \kappa_3)x_4^2 \\
&\leq -\kappa_6 \cdot x_1^2 - \kappa_4 \cdot x_2 + \frac{(2\kappa_5 + \kappa_6 + \kappa_1(1 + b)\mathrm{E_{tot}} + 2b\kappa_1 \mathrm{E_{tot}}^2 + 2\kappa_2 \mathrm{E_{tot}})^2}{4\kappa_6} \\
&\quad + \kappa_5 + (\kappa_2(1 + b) + \kappa_3(2b\mathrm{E_{tot}} + 1))\mathrm{E_{tot}},
\end{aligned}$$

where we have used the fact that $2ab \leq a^2 + b^2$ for the second inequality and the last expression will be less than or equal to -1 whenever x_1 or x_2 is sufficiently large.

Let $C = \{x \in \mathcal{X} : QV(x) > -1\}$. Then, by the above, C consists of finitely many points, which implies that C is a compact set. Then, (S.1) holds with $c = 1$ and $d = 1 + \max_{x \in C} QV(x) \vee 0$. It follows by Theorem S.1 that the Markov chain in Example 4.2 is non-explosive and positive recurrent, and has a unique stationary distribution π.

S.1.3 Application to Example 4.5

For Example 4.5, we first check that the Markov chain is irreducible. For this, consider $x^\circ = (0, \mathrm{D_{tot}}, 0)$ and any fixed state $x = (x_1, x_2, x_3) : 0 \le x_1 + x_2 \le \mathrm{D_{tot}}$. Starting at x°, by having reaction ⑤ fire x_3 times, then reaction ③ fire $\mathrm{D_{tot}} - x_2$ times, and finally, reaction ② fire x_1 times, without any other reactions firing, we see that the Markov chain can transition from x° to x with positive probability. For the reverse transition, by having reaction ⑥ fire x_3 times, then reaction ④ fire x_1 times, and finally, reaction ① fire $\mathrm{D_{tot}} - x_2$ times, we see that the Markov chain can transition from x to x° with positive probability. Thus, the Markov chain is irreducible.

Next we will introduce a norm-like function V and show that (S.2) holds. For each $x \in \mathcal{X}$, let

$$V(x) = x_3.$$

Then $\{x \in \mathcal{X} : V(x) \le a\} = \{x \in \mathbb{Z}^3_+ : x_1 + x_2 \le \mathrm{D_{tot}}, x_3 \le a\}$ consists of finitely many states for each $a \in \mathbb{R}_+$, and for any $x \in \mathcal{X}$,

$$\begin{aligned} QV(x) &= \sum_{j=1}^{6} \Upsilon_j(x) \cdot (V(x + v_j) - V(x)) = \sum_{j=1}^{6} \Upsilon_j(x) \cdot V(v_j) \\ &= \Upsilon_1(x) \cdot 0 + \Upsilon_2(x) \cdot 0 + \Upsilon_3(x) \cdot 0 + \Upsilon_4(x) \cdot 0 + \kappa_{5a} x_2 \cdot 1 + \kappa_{6a} x_3 \cdot (-1) \\ &\le -\kappa_{6a} x_3 + \kappa_{5a} \mathrm{D_{tot}} = -c' V(x) + d', \end{aligned}$$

where $c' = \kappa_{6a}$ and $d' = \kappa_{5a}$. Therefore, we conclude by Theorem S.1 that the Markov chain in Example 4.5 is non-explosive and positive recurrent with a unique stationary distribution π such that $\pi(V) < \infty$, and it is exponentially ergodic in the $(V + 1)$-norm.

S.2 Derivation of Markov chain transition directions, v_j

S.2.1 Example 4.2

The set of reactions associated to the chemical reaction system in Fig. 3(a) is given by $\mathscr{R} = \{(v_1^-, v_1^+), (v_2^-, v_2^+), (v_3^-, v_3^+), (v_4^-, v_4^+), (v_5^-, v_5^+), (v_6^-, v_6^+)\}$, where (v_1^-, v_1^+), (v_2^-, v_2^+), (v_3^-, v_3^+) are defined as in Example 4.1, and $v_4^- = (0, 1, 1, 0)^T$, $v_4^+ = (0, 0, 0, 1)^T$, $v_5^- = v_6^+ = (0, 0, 0, 0)^T$, $v_5^+ = v_6^- = (1, 0, 0, 0)^T$. Then, the potential transitions of the Markov chain are in six possible directions, $v_j = v_j^+ - v_j^-$ for $j = 1, ..., 6$, where $v_1 = -v_2 = (-1, 0, -1, 1)^T$, $v_3 = -v_4 = (0, 1, 1, -1)^T$, and $v_5 = -v_6 = (1, 0, 0, 0)^T$.

S.2.2 Example 4.3

The set of five reactions associated to the chemical reaction system in Fig. 4(a) is given by $\mathscr{R} = \{(v_1^-, v_1^+), (v_2^-, v_2^+), (v_3^-, v_3^+), (v_4^-, v_4^+), (v_5^-, v_5^+)\}$, where $v_1^- = v_3^- = (1, 0, 0, 0)^T$, $v_1^+ = v_2^- = v_5^- = (0, 1, 0, 0)^T$, $v_3^+ = v_4^- = v_5^+ = (0, 0, 1, 0)^T$, $v_2^+ = v_4^+ = (0, 0, 0, 1)^T$. Then, the potential transitions of the Markov chain are in five possible directions, $v_j = v_j^+ - v_j^-$, $j =$

$1, ..., 5$, where $v_1 = (-1, 1, 0, 0)^T$, $v_2 = (0, -1, 0, 1)^T$, $v_3 = (-1, 0, 1, 0)^T$, $v_4 = (0, 0, -1, 1)^T$ and $v_5 = (0, -1, 1, 0)^T$.

S.3 A generalization of Theorem 3.3

Here, we provide a more general version of Theorem 3.3. The simpler form given as Theorem 3.3 in the main text was used there because it is more straightforward to state and the conditions are easier to verify. However, the more general version of the theorem provided in this section can be useful in some cases, such as Example 4.3 (see Section S.3.2).

The generalization relies on the idea that grouping of vectors can be more general than what is described in (3.12), and so we introduce the following assumption.

Assumption S.1. *Consider a collection of distinct vectors $v_1, \ldots, v_n$ in $\mathbb{Z}^d \setminus \{0\}$ and a matrix $A \in \mathbb{Z}^{m\times d}$ with non-zero rows. Suppose that there exists a partition* [8] *$\{G^1, \ldots, G^s\}$ of $\{1, \ldots, n\}$ and an associated bijection $\sigma : \{1, \ldots, n\} \longrightarrow \{1, \ldots, n\}$ such that for $p_0 := 0$ and $1 \le k \le s$, with $p_k := \sum_{\ell=1}^{k} |G^\ell|$, we have $\sigma(q) \in G^k$ for $p_{k-1} + 1 \le q \le p_k$, and whenever $p_{k-1} + 2 \le q \le p_k$, we also have for each $1 \le i \le m$ that either $\langle A_{i\bullet}, v_{\sigma(q)}\rangle$ is equal to $\langle A_{i\bullet}, v_{\sigma(q-1)}\rangle$ or it is 0.*

Theorem S.2. *Consider a non-empty set $\mathcal{X} \subseteq \mathbb{Z}^d_+$, suppose that Assumption S.1 holds and consider two collections of non-negative functions on $\mathcal{X}$, $\Upsilon = (\Upsilon_1, \ldots, \Upsilon_n)$ and $\breve{\Upsilon} = (\breve{\Upsilon}_1, \ldots, \breve{\Upsilon}_n)$, such that (3.3) holds and the associated continuous-time Markov chains do not explode in finite time. Further suppose that both of the following conditions hold:*

(i) For each $1 \le j \le n$, the vector Av_j has entries in $\{-1, 0, 1\}$ only.

(ii) For each $x \in \mathcal{X}$, $1 \le i \le m$ and $y \in \partial_i(K_A + x) \cap \mathcal{X}$ we have that for each $1 \le k \le s$,

$$\sum_{j\in G_i^{k,-}} \breve{\Upsilon}_j(y) \le \sum_{j\in G_i^{k,-}} \Upsilon_j(x), \qquad \text{where } G_i^{k,-} = \{j \in G^k \mid \langle A_{i\bullet}, v_j\rangle = -1\},$$

and

$$\sum_{j\in G_i^{k,+}} \breve{\Upsilon}_j(y) \ge \sum_{j\in G_i^{k,+}} \Upsilon_j(x), \qquad \text{where } G_i^{k,+} = \{j \in G^k \mid \langle A_{i\bullet}, v_j\rangle = 1\}.$$

Then, for each pair $x^\circ, \breve{x}^\circ \in \mathcal{X}$ such that $x^\circ \preccurlyeq_A \breve{x}^\circ$, there exists a probability space $(\Omega, \mathcal{F}, \mathbb{P})$ with two continuous-time Markov chains $X = \{X(t), t \ge 0\}$ and $\breve{X} = \{\breve{X}(t), t \ge 0\}$ defined there, each having state space $\mathcal{X} \subseteq \mathbb{Z}^d_+$, with infinitesimal generators Q and $\breve{Q}$, associated with Υ and $\breve{\Upsilon}$ respectively, with initial conditions $X(0) = x^\circ$ and $\breve{X}(0) = \breve{x}^\circ$ and such that:

$$\mathbb{P}\left[X(t) \preccurlyeq_A \breve{X}(t) \text{ for every } t \ge 0\right] = 1. \tag{S.5}$$

Remark S.2. Under Assumption S.1, for a given $1 \le k \le s$ and $1 \le i \le m$, at most one of $G_i^{k,-}$ and $G_i^{k,+}$ is non-empty. If $G_i^{k,-} \ne \emptyset$, then $G_i^{k,-} = \{\sigma(q) \mid p_{k-1} + 1 \le q \le q^*\}$ where $q^* = \max\{q | p_{k-1}+1 \le q \le p_k \text{ and } \langle A_{i\bullet}, v_{\sigma(q)}\rangle = -1\}$. On the other hand, if $G_i^{k,+} \ne \emptyset$, then $G_i^{k,+} = \{\sigma(q) | p_{k-1}+1 \le q \le q^*\}$ where $q^* = \max\{q | p_{k-1}+1 \le q \le p_k \text{ and } \langle A_{i\bullet}, v_{\sigma(q)}\rangle = 1\}$. Furthermore, in either case, for $q^* < q \le p_k$, we have $\langle A_{i\bullet}, v_{\sigma(q)}\rangle = 0$.

[8] In particular, $\{G^1, \ldots, G^s\}$ is a finite collection of non-empty disjoint sets of distinct numbers, the union of which is $\{1, \ldots, n\}$.

The proof for Theorem S.2 can be found in Section S.3.1. With Theorem S.2 in place, we can extend Theorems 3.4 and 3.5 by adding an additional alternative condition (iv): Assumption S.1 holds, and conditions (i) and (ii) in Theorem S.2 are satisfied. If this is satisfied instead of one of (i) – (iii) in Theorems 3.4 and 3.5, then the conclusions of these theorems about (mean) first passage times and stationary distributions will still hold.

S.3.1 Proof of Theorem S.2

We initially assume that $\sup_{x\in\mathcal{X}} \Upsilon_j(x) < \infty$ and $\sup_{x\in\mathcal{X}} \breve{\Upsilon}_j(x) < \infty$ for every $1 \le j \le n$, and let $\lambda > 0$ such that (5.2) holds. We shall relax these assumptions later. Further suppose that Assumption S.1 and condition (i) of Theorem S.2 both hold. For $x \in \mathcal{X}$, define $I^k(x)$, $I^k_q(x)$, Ψ_λ, $\breve{I}^k(x)$, $\breve{I}^k_q(x)$ and $\breve{\Psi}_\lambda$ in the same manner as in the proof of Theorem 3.3 (see (5.32) – (5.34)), with $\{G^k \mid 1 \le k \le s\}$, $\{p_k \mid 0 \le k \le s\}$ and σ as in Assumption S.1. Our proof of Theorem S.2 has some elements that are the same as those for the proof of Theorem 3.3. However, some additional elements are needed. We give the details for completeness. As for Theorem 3.3, $\Psi_\lambda(\cdot,\cdot)$ and $\breve{\Psi}_\lambda(\cdot,\cdot)$ are well-defined as $\mathcal{X}$-valued functions.

Lemma S.1. *Suppose that $x, y \in \mathcal{X}$ are such that $x \preccurlyeq_A y$ and the following hold: whenever $y \in \partial_i(K_A + x) \cap \mathcal{X}$ for some $1 \le i \le m$, we have that for each $1 \le k \le s$,*

$$\sum_{j\in G_i^{k,-}} \breve{\Upsilon}_j(y) \le \sum_{j\in G_i^{k,-}} \Upsilon_j(x), \qquad \textit{where } G_i^{k,-} = \{j \in G^k \mid \langle A_{i\bullet}, v_j\rangle = -1\}, \tag{S.6}$$

and

$$\sum_{j\in G_i^{k,+}} \breve{\Upsilon}_j(y) \ge \sum_{j\in G_i^{k,+}} \Upsilon_j(x), \qquad \textit{where } G_i^{k,+} = \{j \in G^k \mid \langle A_{i\bullet}, v_j\rangle = 1\}. \tag{S.7}$$

Then, for each $u \in [0,1]$:

$$\Psi_\lambda(x,u) \preccurlyeq_A \breve{\Psi}_\lambda(y,u). \tag{S.8}$$

Proof. First, we note that $\Psi_\lambda, \breve{\Psi}_\lambda$ have the following properties: for every $u \in [0,1]$, $1 \le k \le s$, $j \in G^k$,

$$\text{if } \Psi_\lambda(x,u) = x + v_j, \text{ then } \breve{\Psi}_\lambda(y,u) \in \{y + v_\ell : \ell \in G^k\} \cup \{y\}, \tag{S.9}$$

since $I^k_{\sigma^{-1}(j)}(x), \breve{I}^k_{\sigma^{-1}(\ell)}(y) \subseteq [\frac{p_{k-1}}{n}, \frac{p_k}{n})$ for $\ell \in G^k$. Similarly,

$$\text{if } \breve{\Psi}_\lambda(y,u) = y + v_j, \text{ then } \Psi_\lambda(x,u) \in \{x + v_\ell : \ell \in G^k\} \cup \{x\}. \tag{S.10}$$

Furthermore, for $1 \le k \le s$, $1 \le i \le m$, $j \in G_i^{k,+}$, if $\sum_{\ell\in G_i^{k,+}} \breve{\Upsilon}_\ell(y) \ge \sum_{\ell\in G_i^{k,+}} \Upsilon_\ell(x)$, then

$$\Psi_\lambda(x,u) = x + v_j \text{ implies that } \breve{\Psi}_\lambda(y,u) = y + v_\ell \;\text{ for some }\; \ell \in G_i^{k,+}, \tag{S.11}$$

since under the condition, we have $\cup_{q=p_{k-1}+1}^{q^*} I^k_q(x) \subseteq \cup_{q=p_{k-1}+1}^{q^*} \breve{I}^k_q(y)$ where $q^* = \max\{q | p_{k-1}+1 \le q \le p_k \text{ and } \langle A_{i\bullet}, v_{\sigma(q)}\rangle = 1\}$ and, by Assumption S.1, $G_i^{k,+} = \{\sigma(q)\,|\,p_{k-1}+1 \le q \le q^*\}$. Similarly, for $1 \le k \le s$, $1 \le i \le m$, $j \in G_i^{k,-}$, if $\sum_{\ell\in G_i^{k,-}} \breve{\Upsilon}_\ell(y) \le \sum_{\ell\in G_i^{k,-}} \Upsilon_\ell(x)$, then

$$\breve{\Psi}_\lambda(y,u) = y + v_j \text{ implies that } \Psi_\lambda(x,u) = x + v_\ell \;\text{ for some }\; \ell \in G_i^{k,-}. \tag{S.12}$$

We also have that, for $1 \le k \le s$ and $j \in G^k$, $x \preccurlyeq_A y + v_j$ if and only if

$$\langle A_{i\bullet}, y - x\rangle + \langle A_{i\bullet}, v_j\rangle \ge 0, \qquad \text{for every } 1 \le i \le m. \tag{S.13}$$

Similarly, $x + v_j \preccurlyeq_A y$ if and only if

$$\langle A_{i\bullet}, y - x\rangle - \langle A_{i\bullet}, v_j\rangle \ge 0, \qquad \text{for every } 1 \le i \le m. \tag{S.14}$$

Furthermore, for $1 \le k \le s$ and $j, \ell \in G^k$, $x + v_\ell \preccurlyeq_A y + v_j$ if and only if

$$\langle A_{i\bullet}, y - x\rangle + \langle A_{i\bullet}, v_j - v_\ell\rangle \ge 0, \qquad \text{for every } 1 \le i \le m. \tag{S.15}$$

To prove (S.8), we first consider the situation where $y \in \text{int}(K_A + x) = \{w \in \mathbb{R}^d \mid Ax < Aw\}$. Then, for each $1 \le i \le m$, $\langle A_{i\bullet}, y - x\rangle > 0$ and since $A \in \mathbb{Z}^{m\times d}$ and $y - x \in \mathbb{Z}^d$, we have $\langle A_{i\bullet}, y - x\rangle \ge 1$. This implies that for each $1 \le k \le s$ and $j \in G^k$,

$$\langle A_{i\bullet}, y - x\rangle + \langle A_{i\bullet}, v_j\rangle \ge 1 + \langle A_{i\bullet}, v_j\rangle \ge 0, \qquad \text{for every } 1 \le i \le m, \tag{S.16}$$

since $\langle A_{i\bullet}, v_j\rangle \in \{-1, 0, 1\}$ by condition (i) of Theorem S.2. Similarly, for each $1 \le k \le s$ and $j \in G^k$,

$$\langle A_{i\bullet}, y - x\rangle - \langle A_{i\bullet}, v_j\rangle \ge 1 - \langle A_{i\bullet}, v_j\rangle \ge 0, \qquad \text{for every } 1 \le i \le m. \tag{S.17}$$

In addition, for $1 \le k \le s$ and $j, \ell \in G^k$,

$$\langle A_{i\bullet}, y - x\rangle + \langle A_{i\bullet}, v_j - v_\ell\rangle \ge 1 + \langle A_{i\bullet}, v_j - v_\ell\rangle \ge 0, \qquad \text{for every } 1 \le i \le m, \tag{S.18}$$

since, by Assumption S.1, if $\langle A_{i\bullet}, v_j\rangle \ne 0$, then either $\langle A_{i\bullet}, v_\ell\rangle = \langle A_{i\bullet}, v_j\rangle$ or $\langle A_{i\bullet}, v_\ell\rangle = 0$. It follows from (S.16) – (S.18) that if $y \in \text{int}(K_A + x) \cap \mathcal{X}$, then for any $1 \le k \le s$ and $j, \ell \in G^k$:

$$x \preccurlyeq_A y + v_j,\ x + v_j \preccurlyeq_A y \ \text{ and } \ x + v_\ell \preccurlyeq_A y + v_j. \tag{S.19}$$

We also have, by assumption, that $x \preccurlyeq_A y$. It follows that if $y \in \text{int}(K_A + x) \cap \mathcal{X}$, then $\{x, x + v_\ell \mid \ell \in G^k\} \preccurlyeq_A \{y, y + v_j \mid j \in G^k\}$ for $1 \le k \le s$ and consequently (S.8) holds for all $u \in [0, 1]$.

Now, we turn to the other situation where $y \in \partial_i(K_A + x) \cap \mathcal{X}$ for some $1 \le i \le m$. Then $\mathbf{K}_y := \{i \mid \langle A_{i\bullet}, y\rangle = \langle A_{i\bullet}, x\rangle, 1 \le i \le m\}$ is non-empty. Let $u \in [0, 1]$. We consider two cases.

Case 1: $\breve{\Psi}_\lambda(y, u) = y + v_j$ for some $1 \le j \le n$.

Fix such an index j. Consider the unique $1 \le k \le s$ such that $j \in G^k$. Then, by (S.10), either $\Psi_\lambda(x, u) = x + v_\ell$ for some $\ell \in G^k$, or $\Psi_\lambda(x, u) = x$.

a) Suppose $\Psi_\lambda(x, u) = x + v_\ell$ for some $\ell \in G^k$. Observe that for every $i \notin \mathbf{K}_y$, $\langle A_{i\bullet}, y - x\rangle > 0$ and as for (S.18), $\langle A_{i\bullet}, (y + v_j) - (x + v_\ell)\rangle \ge 0$, while for $i \in \mathbf{K}_y$, $\langle A_{i\bullet}, (y + v_j) - (x + v_\ell)\rangle = \langle A_{i\bullet}, v_j\rangle - \langle A_{i\bullet}, v_\ell\rangle$. For each $i \in \mathbf{K}_y$,

   i) if $\langle A_{i\bullet}, v_j\rangle = -1$ and $\langle A_{i\bullet}, v_\ell\rangle = 0$, then $j \in G_i^{k,-}$ and $\ell \notin G_i^{k,-}$. By (S.6), we would then have $\sum_{r\in G_i^{k,-}} \breve{\Upsilon}_r(y) \le \sum_{r\in G_i^{k,-}} \Upsilon_r(x)$, which would imply by (S.12) that $\Psi_\lambda(x, u) = x + v_r$ for some $r \in G_i^{k,-}$. Since we are assuming that $\Psi_\lambda(x, u) = x + v_\ell$ and we know the vectors $v_1, \ldots, v_n$ are distinct, we obtain that $\ell = r \in G_i^{k,-}$. This contradicts $\ell \notin G_i^{k,-}$.

ii) if $\langle A_{i\bullet}, v_j\rangle = 0$ and $\langle A_{i\bullet}, v_\ell\rangle = 1$, then $j \notin G_i^{k,+}$ and $\ell \in G_i^{k,+}$. By (S.7), we would then have $\sum_{r\in G_i^{k,+}} \breve{\Upsilon}_r(y) \geq \sum_{r\in G_i^{k,+}} \Upsilon_r(x)$, which would imply by (S.11) that $j \in G_i^{k,+}$. This contradicts $j \notin G_i^{k,+}$.

iii) in all the other cases, that is when

$$(\langle A_{i\bullet}, v_j\rangle, \langle A_{i\bullet}, v_\ell\rangle) \in \{(1,0),(0,-1),(1,1),(0,0),(-1,-1)\},$$

we have $\langle A_{i\bullet}, (y+v_j)-(x+v_\ell)\rangle \geq 0$.

Combining the above for case a), we see that $\langle A_{i\bullet}, (y+v_j)-(x+v_\ell)\rangle \geq 0$ for each $1 \leq i \leq m$, which implies that $\Psi_\lambda(x,u) = x + v_\ell \preccurlyeq_A y + v_j = \breve{\Psi}_\lambda(y,u)$.

b) Suppose $\Psi_\lambda(x,u) = x$. We claim that $y + v_j \in K_A + x$. To see this, observe that for every $i \notin \mathbf{K}_y$, $\langle A_{i\bullet}, y-x\rangle > 0$ and as for (S.16), $\langle A_{i\bullet}, (y+v_j)-x\rangle \geq 0$, while for $i \in \mathbf{K}_y$, $\langle A_{i\bullet}, (y+v_j)-x\rangle = \langle A_{i\bullet}, v_j\rangle \in \{-1,0,1\}$. For each $i \in \mathbf{K}_y$, if $\langle A_{i\bullet}, v_j\rangle = -1$, which means $j \in G_i^{k,-}$, then by (S.6) we would have $\sum_{\ell\in G_i^{k,-}} \breve{\Upsilon}_\ell(y) \leq \sum_{\ell\in G_i^{k,-}} \Upsilon_\ell(x)$, which would imply by (S.12) that $\Psi_\lambda(x,u) = x + v_\ell$ for some $\ell \in G_i^{k,-}$, but this contradicts the assumption that $\Psi_\lambda(x,u) = x$. So we must have $\langle A_{i\bullet}, v_j\rangle \geq 0$ and hence $\langle A_{i\bullet}, (y+v_j)-x\rangle \geq 0$ for all $i \in \mathbf{K}_y$. Thus, $y + v_j \in K_A + x$ and so $\Psi_\lambda(x,u) = x \preccurlyeq_A y + v_j = \breve{\Psi}_\lambda(y,u)$.

Case 2: $\breve{\Psi}_\lambda(y,u) = y$. Again, we consider two subcases.

a) If $\Psi_\lambda(x,u) = x$, then (S.8) holds, because $x \preccurlyeq_A y$.

b) If $\Psi_\lambda(x,u) = x + v_j$ for some $1 \leq j \leq n$, we claim that $y \in K_A + x + v_j$ for the corresponding value of j. To see this, fix the value of j for which $\Psi_\lambda(x,u) = x + v_j$, let $1 \leq k \leq s$ be such that $j \in G^k$, and observe that for every $i \notin \mathbf{K}_y$, $\langle A_{i\bullet}, y-x\rangle > 0$ and as for (S.17), $\langle A_{i\bullet}, y-(x+v_j)\rangle \geq 0$, while for $i \in \mathbf{K}_y$, $\langle A_{i\bullet}, y-(x+v_j)\rangle = -\langle A_{i\bullet}, v_j\rangle \in \{-1,0,1\}$. For each $i \in \mathbf{K}_y$, if $\langle A_{i\bullet}, v_j\rangle = 1$, which means $j \in G_i^{k,+}$, then by (S.7), we would have $\sum_{\ell\in G_i^{k,+}} \breve{\Upsilon}_\ell(y) \geq \sum_{\ell\in G_i^{k,+}} \Upsilon_\ell(x)$, which would imply by (S.11) that $\breve{\Psi}_\lambda(y,u) = y + v_\ell$ for some $\ell \in G_i^{k,+}$, but this contradicts the assumption that $\breve{\Psi}_\lambda(y,u) = y$. So we must have $\langle A_{i\bullet}, v_j\rangle \leq 0$ and hence $\langle A_{i\bullet}, y-(x+v_j)\rangle \geq 0$ for all $i \in \mathbf{K}_y$. Thus, we have $y \in K_A + x + v_j$ and then $\Psi_\lambda(x,u) = x + v_j \preccurlyeq_A y = \breve{\Psi}_\lambda(y,u)$.

□

In order to prove Theorem S.2, from here on we can follow a similar procedure to the one used in the proof of Theorem 3.1 after Lemma 5.1 was proved there. For the case where (5.1) holds, we define two discrete-time processes, $Y = (Y_k)_{k\geq 0}$ and $\breve{Y} = (\breve{Y}_k)_{k\geq 0}$, by defining $Y_0 := x^\circ$, $\breve{Y}_0 := \breve{x}^\circ$, and for $k \geq 0$,

$$Y_{k+1} := \Psi_\lambda(Y_k, U_{k+1}), \qquad \breve{Y}_{k+1} := \breve{\Psi}_\lambda(\breve{Y}_k, U_{k+1}), \tag{S.20}$$

and define X and $\breve{X}$ using these and an independent Poisson process N as in (5.13). For the case where (5.1) does not hold, we can use a truncation procedure similar to that for Theorem 3.1. In both cases, we use Lemma S.1 instead of Lemma 5.1.

S.3.2 Two other A matrices for Example 4.3

Let

$$A = \begin{bmatrix} -1 & 0 & 0 & 0 \\ 0 & 0 & -1 & 0 \\ 0 & -1 & -1 & 0 \end{bmatrix}. \tag{S.21}$$

For $x \in \mathcal{X}$, consider infinitesimal transition rates $\breve{\Upsilon}_1(x), \breve{\Upsilon}_2(x), \breve{\Upsilon}_3(x), \breve{\Upsilon}_4(x)$ and $\breve{\Upsilon}_5(x)$ defined as for $\Upsilon_1(x), \Upsilon_2(x), \Upsilon_3(x), \Upsilon_4(x)$ and $\Upsilon_5(x)$ in (4.7), but with $\breve{\kappa}_i$ in place of κ_i where $\breve{\kappa}_i = \kappa_i$, for $i = 1,2,3,4$, and $\breve{\kappa}_5 \leq \kappa_5$. Suppose that $\kappa_2 > \kappa_4$. Now, let us verify that the assumptions of Theorem S.2 hold. Condition (i) holds since $Av_1 = (1,0,-1)^T$, $Av_2 = (0,0,1)^T$, $Av_3 = (1,-1,-1)^T$, $Av_4 = (0,1,1)^T$ and $Av_5 = (0,-1,0)^T$. Assumption S.1 holds with $G^1 = \{3,1\}$, $G^2 = \{4,2\}$, $G^3 = \{5\}$ and $\sigma(1) = 3$, $\sigma(2) = 1$, $\sigma(3) = 4$, $\sigma(4) = 2$, $\sigma(5) = 5$. To verify that condition (ii) of Theorem S.2 holds, fix $x \in \mathcal{X}$ and first consider $y \in \partial_1(K_A + x) \cap \mathcal{X}$, where $\partial_1(K_A + x) \cap \mathcal{X} = \{w \in \mathcal{X} \mid x_1 = w_1, x_3 \geq w_3, x_2 + x_3 \geq w_2 + w_3, x_4 \leq w_4\}$. Given that $\langle A_{1\bullet}, v_1\rangle = \langle A_{1\bullet}, v_3\rangle = 1$, we need to check that $\Upsilon_1(x)+\Upsilon_3(x) \leq \breve{\Upsilon}_1(y)+\breve{\Upsilon}_3(y)$. Since $y \in \partial_1(K_A+x)\cap\mathcal{X}$, then $\Upsilon_1(x) = \kappa_1 x_1 = \kappa_1 y_1 = \breve{\kappa}_1 y_1 = \breve{\Upsilon}_1(y)$ and $\Upsilon_3(x) = \kappa_3 x_1 = \kappa_3 y_1 = \breve{\kappa}_3 y_1 = \breve{\Upsilon}_3(y)$, and so the desired inequality holds with equality. Secondly, consider $y \in \partial_2(K_A + x) \cap \mathcal{X} = \{w \in \mathcal{X} \mid x_1 \geq w_1, x_3 = w_3, x_2 \geq w_2, x_4 \leq w_4\}$. Given that $\langle A_{2\bullet}, v_3\rangle = \langle A_{2\bullet}, v_5\rangle = -1$ and $\langle A_{2\bullet}, v_4\rangle = 1$, we need to check that $\Upsilon_3(x) \geq \breve{\Upsilon}_3(y)$, $\Upsilon_4(x) \leq \breve{\Upsilon}_4(y)$ and $\Upsilon_5(x) \geq \breve{\Upsilon}_5(y)$. Since $y \in \partial_2(K_A + x) \cap \mathcal{X}$, then $\Upsilon_3(x) = \kappa_3 x_1 \geq \kappa_3 y_1 = \breve{\kappa}_3 y_1 = \breve{\Upsilon}_3(y)$, $\Upsilon_4(x) = \kappa_4 x_3 = \kappa_4 y_3 = \breve{\kappa}_4 y_3 = \breve{\Upsilon}_4(y)$ and $\Upsilon_5(x) = \kappa_5 x_2 \geq \kappa_5 y_2 \geq \breve{\kappa}_5 y_2 = \breve{\Upsilon}_5(y)$, and so the desired inequality holds. Lastly, consider $y \in \partial_3(K_A + x) \cap \mathcal{X} = \{w \in \mathcal{X} \mid x_1 \geq w_1, x_3 \geq w_3, x_2 + x_3 = w_2 + w_3, x_4 \leq w_4\}$. Given that $\langle A_{1\bullet}, v_1\rangle = \langle A_{1\bullet}, v_3\rangle = -1$ and $\langle A_{1\bullet}, v_2\rangle = \langle A_{1\bullet}, v_4\rangle = 1$, we need to check that $\Upsilon_1(x) + \Upsilon_3(x) \geq \breve{\Upsilon}_1(y) + \breve{\Upsilon}_3(y)$ and $\Upsilon_2(x) + \Upsilon_4(x) \leq \breve{\Upsilon}_2(y) + \breve{\Upsilon}_4(y)$. For $y \in \partial_3(K_A + x) \cap \mathcal{X}$, since $\kappa_2 > \kappa_4$ was assumed, we have that $\Upsilon_2(x) + \Upsilon_4(x) = \kappa_2 x_2 + \kappa_4 x_3 = (\kappa_2 - \kappa_4)x_2 + \kappa_4(x_2 + x_3) \leq (\kappa_2 - \kappa_4)y_2 + \kappa_4(y_2 + y_3) = \breve{\kappa}_2 y_2 + \breve{\kappa}_4 y_3 = \breve{\Upsilon}_2(y) + \breve{\Upsilon}_4(y)$ and $\Upsilon_1(x) = \kappa_1 x_1 \geq \kappa_1 y_1 = \breve{\kappa}_1 y_1 = \breve{\Upsilon}_1(y)$, $\Upsilon_3(x) = \kappa_3 x_1 \geq \kappa_3 y_1 = \breve{\kappa}_3 y_1 = \breve{\Upsilon}_3(y)$. Thus, the conditions of Theorem S.2 are satisfied and so the conclusion of that theorem holds.

Let $\Gamma = \{(0,0,0,\mathrm{S}_{\mathrm{tot}})\}$. This is an increasing set in $\mathcal{X}$ with respect to the relation $\preccurlyeq_A$. Let $T_{(0,0,0,\mathrm{S}_{\mathrm{tot}})}$, respectively $\breve{T}_{(0,0,0,\mathrm{S}_{\mathrm{tot}})}$ be the first time that the Markov chain X, respectively $\breve{X}$, reaches the set Γ. Then, by the generalization of Theorem 3.4, if $X(0) = \breve{X}(0) = (\mathrm{S}_{\mathrm{tot}}, 0, 0, 0)$, we have that $\breve{T}_{(0,0,0,\mathrm{S}_{\mathrm{tot}})} \preccurlyeq_{st} T_{(0,0,0,\mathrm{S}_{\mathrm{tot}})}$. It follows that increasing κ_5 will increase the mean first passage time from $(\mathrm{S}_{\mathrm{tot}}, 0, 0, 0)$ to $(0,0,0,\mathrm{S}_{\mathrm{tot}})$ when $\kappa_2 > \kappa_4$ (See Figure S.1). Indeed, when $\kappa_2 > \kappa_4$, it takes a longer time to get to $(0,0,0,\mathrm{S}_{\mathrm{tot}})$ from $(\mathrm{S}_{\mathrm{tot}}, 0, 0, 0)$ if reaction ⑤ is added to the system without that reaction.

On the other hand, suppose

$$A = \begin{bmatrix} -1 & 0 & 0 & 0 \\ 0 & -1 & 0 & 0 \\ 0 & -1 & -1 & 0 \end{bmatrix}$$

and infinitesimal transition rates $\breve{\Upsilon}_1(x), \breve{\Upsilon}_2(x), \breve{\Upsilon}_3(x), \breve{\Upsilon}_4(x)$ and $\breve{\Upsilon}_5(x)$ are defined as for $\Upsilon_1(x)$,$\Upsilon_2(x)$, $\Upsilon_3(x)$,$\Upsilon_4(x)$ and $\Upsilon_5(x)$ in (4.7), but with $\breve{\kappa}_i$ in place of κ_i where $\breve{\kappa}_i = \kappa_i$, for $i = 1,2,3,4$, $\breve{\kappa}_5 \geq \kappa_5$, and $\kappa_2 < \kappa_4$. We can verify that the assumptions of Theorem S.2 hold, as follows. Condition (i) holds since $Av_1 = (1,-1,-1)^T$, $Av_2 = (0,1,1)^T$, $Av_3 = (1,0,-1)^T$, $Av_4 = (0,0,1)^T$ and $Av_5 = (0,1,0)^T$. Assumption S.1 holds with $G^1 = \{1,3\}$, $G^2 = \{2,4\}$,

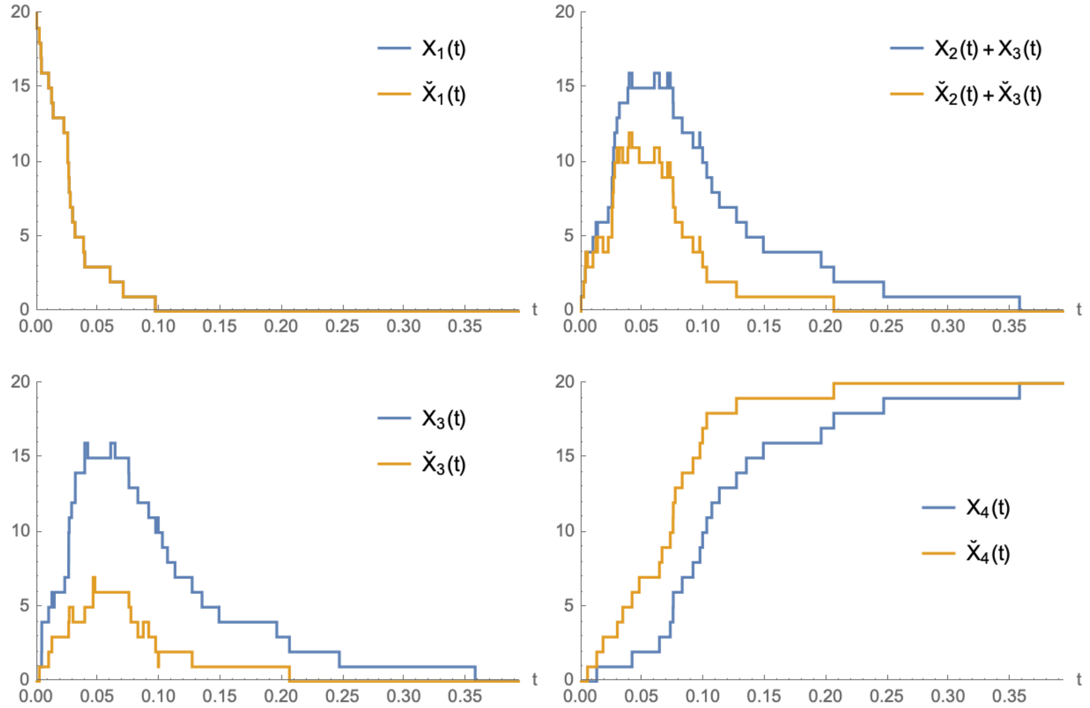

Figure S.1: **A typical coupled realization of sample paths for Example 4.3.** Here $\mathrm{S_{tot}} = 20$, $\kappa_1 = \breve{\kappa}_1 = 30$, $\kappa_2 = \breve{\kappa}_2 = 50$, $\kappa_3 = \breve{\kappa}_3 = 10$, $\kappa_4 = \breve{\kappa}_4 = 10$, $\kappa_5 = 1000$ and $\breve{\kappa}_5 = 10$. Both processes X and $\breve{X}$ start at $(\mathrm{S_{tot}}, 0, 0, 0)$. As shown in Section S.3.2, for these parameters, we have, almost surely, $X(t) \preccurlyeq_A \breve{X}(t)$ for every $t \geq 0$ where the matrix A is given in (S.21). A coupled realization using the algorithm described in Section S.4 is plotted to illustrate this result. In particular, we see in this sample that $X_1(t) \geq \breve{X}_1(t)$, $X_2(t) + X_3(t) \geq \breve{X}_2(t) + \breve{X}_3(t)$ and $X_3(t) \geq \breve{X}_3(t)$ for all times t. Moreover, the first passage time to $(0, 0, 0, \mathrm{S_{tot}})$ for X (which is equivalent to the first time to get to the state where $X_4 = \mathrm{S_{tot}}$) is larger than for $\breve{X}$. Since this first passage time result is true for all coupled samples, we can conclude that the mean first passage time from $(\mathrm{S_{tot}}, 0, 0, 0)$ to $(0, 0, 0, \mathrm{S_{tot}})$ is larger for X than for $\breve{X}$.

$G^3 = \{5\}$ and $\sigma(1) = 1$, $\sigma(2) = 3$, $\sigma(3) = 2$, $\sigma(4) = 4$, $\sigma(5) = 5$. To verify that condition (ii) of Theorem S.2 holds, let $x \in \mathcal{X}$, and first consider $y \in \partial_1(K_A + x) \cap \mathcal{X}$, where $\partial_1(K_A + x) \cap \mathcal{X} = \{w \in \mathcal{X} \mid x_1 = w_1, x_2 \geq w_2, x_2 + x_3 \geq w_2 + w_3, x_4 \leq w_4\}$. Given that $\langle A_{1\bullet}, v_1\rangle = \langle A_{1\bullet}, v_3\rangle = 1$, we need to check that $\Upsilon_1(x) + \Upsilon_3(x) \leq \breve{\Upsilon}_1(y) + \breve{\Upsilon}_3(y)$. Since $y \in \partial_1(K_A + x) \cap \mathcal{X}$, then $\Upsilon_1(x) = \kappa_1 x_1 = \kappa_1 y_1 = \breve{\kappa}_1 y_1 = \breve{\Upsilon}_1(y)$ and $\Upsilon_3(x) = \kappa_3 x_1 = \kappa_3 y_1 = \breve{\kappa}_3 y_1 = \breve{\Upsilon}_3(y)$, and so the desired inequality holds with equality. Secondly, consider $y \in \partial_2(K_A + x) \cap \mathcal{X} = \{w \in \mathcal{X} \mid x_1 \geq w_1, x_2 = w_2, x_2 + x_3 \geq w_2 + w_3, x_4 \leq w_4\}$. Given that $\langle A_{2\bullet}, v_2\rangle = \langle A_{2\bullet}, v_5\rangle = 1$ and $\langle A_{2\bullet}, v_1\rangle = -1$, we need to check that $\Upsilon_1(x) \geq \breve{\Upsilon}_1(y)$, $\Upsilon_2(x) \leq \breve{\Upsilon}_2(y)$ and $\Upsilon_5(x) \leq \breve{\Upsilon}_5(y)$. Since $y \in \partial_2(K_A + x) \cap \mathcal{X}$, then $\Upsilon_1(x) = \kappa_1 x_1 \geq \kappa_1 y_1 = \breve{\kappa}_1 y_1 = \breve{\Upsilon}_1(y)$, $\Upsilon_2(x) = \kappa_2 x_2 = \kappa_2 y_2 = \breve{\kappa}_2 y_2 = \breve{\Upsilon}_2(y)$ and $\Upsilon_5(x) = \kappa_5 x_2 = \kappa_5 y_2 \leq \breve{\kappa}_5 y_2 = \breve{\Upsilon}_5(y)$, and so the desired inequality holds. Lastly, consider $y \in \partial_3(K_A + x) \cap \mathcal{X} = \{w \in \mathcal{X} \mid x_1 \geq w_1, x_2 \geq w_2, x_2 + x_3 = w_2 + w_3, x_4 \leq w_4\}$. Given that $\langle A_{1\bullet}, v_1\rangle = \langle A_{1\bullet}, v_3\rangle = -1$ and $\langle A_{1\bullet}, v_2\rangle = \langle A_{1\bullet}, v_4\rangle = 1$, we need to check that $\Upsilon_1(x) + \Upsilon_3(x) \geq \breve{\Upsilon}_1(y) + \breve{\Upsilon}_3(y)$ and $\Upsilon_2(x) + \Upsilon_4(x) \leq \breve{\Upsilon}_2(y) + \breve{\Upsilon}_4(y)$. For $y \in \partial_3(K_A + x) \cap \mathcal{X}$, since $\kappa_2 < \kappa_4$, we have that $\Upsilon_1(x) + \Upsilon_3(x) = (\kappa_1 + \kappa_3)x_1 \geq (\kappa_1 + \kappa_3)y_1 = (\breve{\kappa}_1 + \breve{\kappa}_3)y_1 = \breve{\Upsilon}_1(y) + \breve{\Upsilon}_3(y)$ and

$\Upsilon_2(x) + \Upsilon_4(x) = \kappa_2 x_2 + \kappa_4 x_3 = (\kappa_4 - \kappa_2)x_3 + \kappa_2(x_2 + x_3) \le (\kappa_4 - \kappa_2)y_3 + \kappa_2(y_2 + y_3) = \breve{\kappa}_2 y_2 + \breve{\kappa}_4 y_3 = \breve{\Upsilon}_2(y) + \breve{\Upsilon}_4(y)$. Thus, the conditions of Theorem S.2 are satisfied. In particular, we can conclude when $\kappa_2 < \kappa_4$, that it takes less time to get to $(0,0,0,\mathrm{S}_{\mathrm{tot}})$ from $(\mathrm{S}_{\mathrm{tot}},0,0,0)$ if reaction ⑤ is added to the system without that reaction[9].

S.4 An algorithm for coupled stochastic simulation

We now provide an algorithm for stochastic simulation of the coupled continuous-time Markov chains X and $\breve{X}$ under the conditions of Theorems 3.1, 3.2, 3.3 or S.2, when the transitions rates are bounded on the state space, i.e., when (5.1) holds.

Algorithm: Stochastic simulation for coupled continuous-time Markov chains X and $\breve{X}$.

Data: Integer $n \ge 1$, real $T > 0$, set $\mathcal{X} \subseteq \mathbb{Z}^d_+$, vectors $v_1, \dots, v_n$ in $\mathbb{Z}^d \setminus \{0\}$, $x^\circ, \breve{x}^\circ$ in $\mathcal{X}$, functions $\Upsilon = (\Upsilon_1, \dots, \Upsilon_n)$ and $\breve{\Upsilon} = (\breve{\Upsilon}_1, \dots, \breve{\Upsilon}_n)$ and integer $a \in \{1,2,3,4\}$ to indicate which theorem is invoked (3.1, 3.2, 3.3 or S.2).

Result: Sample of initial time and subsequent potential jump times $T_0, T_1, \dots, T_N$ and associated states $X(T_0), X(T_1), \dots, X(T_N)$ and $\breve{X}(T_0), \breve{X}(T_1), \dots, \breve{X}(T_N)$ for the continuous-time Markov chains X and $\breve{X}$ in the time interval $[0,T]$.

```
λ ← 1 + n max { sup_{x∈𝒳} Σ_{j=1}^n Υ_j(x), sup_{x∈𝒳} Σ_{j=1}^n Ῠ_j(x) } ;
K ← 0;
T_0 ← 0;
while T_K ≤ T do
    T_{K+1} ← T_K + Exponential(λ);
    K ← K + 1;
end
Y_0, Y̆_0 ← x°, x̆°;
X(T_0), X̆(T_0) ← x°, x̆°;
N ← K − 1;
if N ≥ 1 then
    for k ← 0 to N − 1 do
        U ← Uniform([0,1]);
        Y_{k+1}, Y̆_{k+1} ← TransitionDTMC(Υ, λ, Y_k, U, a),
         TransitionDTMC(Ῠ, λ, Y̆_k, U, a);
        X(T_{k+1}), X̆(T_{k+1}) ← Y_{k+1}, Y̆_{k+1};
    end
end
```

The random variables $T_1, \dots, T_N$ are called potential jump times because it could be that $X(T_k) = X(T_{k+1})$ or $\breve{X}(T_k) = \breve{X}(T_{k+1})$ for some $0 \le k \le N-1$. Letting $T_{N+1} := T$, the trajectories of X are given by $X(t) = X(T_k)$ for $T_k \le t < T_{k+1}$, $0 \le k \le N$, and similarly for the trajectories of $\breve{X}$.

[9] The system without reaction ⑤ can be obtained from the system with reaction ⑤ by setting $\kappa_5 = 0$. While strictly speaking a zero rate constant is not within our definition of mass action kinetics, our theory does cover propensity functions with such a zero rate constant.

The function `TransitionDTMC` can be found below. This function is meant to replicate $\Phi_\lambda(x,u)$ in (5.4) for the case of Theorems 3.1 and 3.2, and $\Psi_\lambda(x,u)$ in (5.34) for the case of Theorems 3.3 and S.2.

Function `TransitionDTMC(`$\Upsilon, \lambda, x, u, a$`)`

Data: Integer $n \geq 1$, set $\mathcal{X} \subseteq \mathbb{Z}^d_+$, vectors $v_1, \ldots, v_n$ in $\mathbb{Z}^d \setminus \{0\}$.

Input: Function $\Upsilon = (\Upsilon_1, \ldots, \Upsilon_n)$, $\lambda > 0, x \in \mathcal{X}, u \in [0,1]$. Integer $a \in \{1,2,3,4\}$ to indicate which theorem is invoked (3.1, 3.2, 3.3 or S.2). For the case of Theorem 3.3 or S.2, include partition $\{G^1, \ldots, G^s\}$ and bijection σ.

Output: State $x + v \in \mathcal{X}$.

$v \leftarrow 0$;
if $a \in \{1,2\}$ **then** `// The case of Theorem 3.1 or 3.2.`
  for $j \leftarrow 1$ **to** n **do**
    if $\frac{j-1}{n} \leq u < \frac{j-1}{n} + \frac{\Upsilon_j(x)}{\lambda}$ **then**
      $v \leftarrow v_j$;
    end
  end
end
else `// The case of Theorem 3.3 or S.2.`
  $p_0 \leftarrow 0$;
  for $k \leftarrow 1$ **to** s **do**
    $p_k \leftarrow p_{k-1} + |G^k|$;
    for $q \leftarrow p_{k-1}+1$ **to** p_k **do**
      if $\frac{p_{k-1}}{n} + \sum_{\ell=p_{k-1}+1}^{q-1} \frac{\Upsilon_{\sigma(\ell)}(x)}{\lambda} \leq u < \frac{p_{k-1}}{n} + \sum_{\ell=p_{k-1}+1}^{q} \frac{\Upsilon_{\sigma(\ell)}(x)}{\lambda}$ **then**
        $v \leftarrow v_{\sigma(q)}$;
      end
    end
  end
end
return x+v

Remark S.3. The above algorithm can be adapted to provide simultaneous stochastic simulation for X and $\check{X}$ when transition rates are not bounded on the state space, by applying the algorithm on a sequence of bounded sets, which expand to the whole state space. This employs a sequence of successively defined stopping times $\tau_0 = 0$, $\tau_\ell = \inf\{t \geq \tau_{\ell-1} \mid X(t) \notin C_\ell \text{ or } \check{X}(t) \notin C_\ell\}$, $\ell = 1, 2, \ldots$, where the C_ℓ are compact, $C_\ell \subseteq C_{\ell+1}$ for $\ell = 1, 2, \ldots$ and $\cup_{\ell=1}^\infty C_\ell = \mathcal{X}$. The simulation of the pair $(X, \check{X})(t)$ for $\tau_\ell \leq t < \tau_{\ell+1}$ uses the above algorithm on C_ℓ for $\ell = 1, 2, \ldots$.

References

[1] Meyn, S. P., and Tweedie, R. L.(1993). Stability of Markovian processes iii: Foster–Lyapunov criteria for continuous-time processes. *Advances in Applied Probability*, 25(3):518—548.

[2] Sharpe, M. (1988). *General Theory of Markov Processes*. Academic Press.
